# Chromosome organization by a conserved condensin-ParB system in the actinobacterium *Corynebacterium glutamicum*

**DOI:** 10.1038/s41467-020-15238-4

**Published:** 2020-03-20

**Authors:** Kati Böhm, Giacomo Giacomelli, Andreas Schmidt, Axel Imhof, Romain Koszul, Martial Marbouty, Marc Bramkamp

**Affiliations:** 1 Ludwig-Maximilians-Universität München, Fakultät Biologie, 82152 Planegg-Martinsried, Germany; 2 Christian-Albrechts-Universität zu Kiel, Institut für allgemeine Mikrobiologie, 24118 Kiel, Germany; 3 Biomedical Center, Protein Analysis Unit, Faculty of Medicine, Ludwig-Maximilians-Universität München, 82152 Planegg-Martinsried, Germany; 4 Institut Pasteur, Unité Régulation Spatiales des Génomes, UMR3525, CNRS, F-75015 Paris, France; 5 Institut Pasteur, Center of Bioinformatics, Biostatistics and Integrative Biology (C3BI), Paris, France

**Keywords:** Chromosome segregation, Bacteria, Cellular microbiology

## Abstract

Higher-order chromosome folding and segregation are tightly regulated in all domains of life. In bacteria, details on nucleoid organization regulatory mechanisms and function remain poorly characterized, especially in non-model species. Here, we investigate the role of DNA-partitioning protein ParB and SMC condensin complexes in the actinobacterium *Corynebacterium glutamicum*. Chromosome conformation capture reveals SMC-mediated long-range interactions around ten centromere-like *parS* sites clustered at the replication origin (*oriC*). At least one *oriC*-proximal *parS* site is necessary for reliable chromosome segregation. We use chromatin immunoprecipitation and photoactivated single-molecule localization microscopy to show the formation of distinct, *parS*-dependent ParB-nucleoprotein subclusters. We further show that SMC/ScpAB complexes, loaded via ParB at *parS* sites, mediate chromosomal inter-arm contacts (as previously shown in *Bacillus subtilis*). However, the MukBEF-like SMC complex MksBEFG does not contribute to chromosomal DNA-folding; instead, this complex is involved in plasmid maintenance and interacts with the polar *oriC*-tethering factor DivIVA. Our results complement current models of ParB-SMC/ScpAB crosstalk and show that some condensin complexes evolved functions that are apparently uncoupled from chromosome folding.

## Abstract (web summary)

The regulation of higher-order chromosome folding and segregation in bacteria is poorly understood. Here, Böhm et al. provide insights into the roles of DNA partitioning protein ParB and SMC condensin complexes in *Corynebacterium glutamicum*.

## Introduction

Each organism must complete genome replication and separation in the course of one cell cycle prior to cell division in concert with transcriptional processes. To this end, chromosomes are highly organized structures in terms of segregation and overall folding patterns^1^. The functional organization of bacterial genomes, structured into the nucleoid, has been predominantly investigated in a limited number of model species, e.g., *Escherichia coli*, *Vibrio cholerae*, *Bacillus subtilis*, or *Caulobacter crescentus*, revealing diverse levels of compaction and segregation strategies^2–4^.

ParABS systems and condensins are two (nearly) ubiquitous bacterial enzyme machineries that contribute to chromosome homeostasis. With a few exceptions among γ-proteobacteria, including *E. coli*, all branches of bacteria and several Archaea harbor *parS* sites that recruit partitioning protein ParB^5^. The ParABS system contains one or several *parS* sites usually in the vicinity to the chromosomal origin of replication (*oriC*). ParB proteins bind to these sequence-specific motives and form large nucleocomplexes by spreading and three-dimensional (3D)-bridging between ParB dimers^6–9^, resulting in large chromosome interaction domains promoting encompassing the *oriC*, which have been revealed by chromosome conformation capture coupled to deep sequencing (Hi-C) for *B. subtilis*^10^. In an alternative model termed nucleation and caging, ParB nucleation at *parS* is stabilized by dynamic ParB dimer–dimer interactions and weak interactions with nonspecific DNA generating a scaffold for locally high ParB concentrations confined around *parS*^11^. The ParB segregation is driven by a ParA ATPase, which binds nonspecifically to the nucleoid and is released from DNA upon ATP hydrolysis triggered by transient ParB interactions^12,13^. In the course of chromosome replication, ParB-*oriC* complexes act in combination with ParA as Brownian ratchets along dynamic DNA loci: slow ParA-DNA rebinding rates generate ParA gradients, which serve as tracks for directed movement of partition complexes away from their sisters^14–17^. Perturbation of the system by placing *parS* sites at ectopic, *oriC*-distal regions can cause severe DNA-segregation phenotypes^18,19^. To date, only few studies investigated the impact of chromosomal *parS* localization on DNA segregation and folding^18–22^.

In addition to ParABS systems, most bacteria harbor condensin complexes, members of the structural maintenance of chromosomes (SMCs) family of proteins found in all kingdoms of life^23^. In standard model organisms, condensins are equally essential for faithful chromosome segregation by compacting DNA into separate nucleoids^24–26^. The SMC/ScpAB complex is well-studied in *B. subtilis*, where it consists of two large SMC subunits and the kleisin ScpA associated with dimeric accessory protein ScpB that assemble into a ring-like structure^27^. A recent study suggests progressive extrusion of condensin-encircled DNA loops upon conformational changes in the SMC subunit, which leads to a gradual size increase of trapped DNA molecules^28^. The active process(es) driving DNA extrusion^29,30^ allow(s) for translocation along the chromosome with velocities of around 50 kb/min (ref. ^31^) and depend(s) on the ATPase activity of SMC^32,33^. To be loaded on *parS* sites, SMC/ScpAB complexes necessitate ParB^20,22,34,35^. They redistribute to distant chromosomal regions, promoting the co-alignment of the right and left replichores^10,21,22,31,36^. In sharp contrast with SMC/ScpAB, the *E. coli* condensin MukBEF does not promote the co-alignment of chromosomal arms^37,38^, but facilitate *cis*-structuration by establishing long-range contacts between loci belonging to the same replichores from stochastically positioned chromosomal loci (except for the *ter* region containing the replication terminus)^38,39^. Despite the importance of condensins in chromosome organization, the role of SMC homologs besides the model species *B. subtilis*, *C. crescentus*, and *E. coli* remain largely unexplored. These species all contain a single condensin complex, yet a broad range of bacteria possesses combinations of SMC/ScpAB and MksBEFG (MukB-like SMC), for which functional characterizations are non-existent to date^40^. Current work in bacteria and in eukaryotes convey the general assumption that all SMCs are likely to play role(s) in chromosome organization. In bacteria, it remains unknown why some species harbor more than one type of condensin, and whether and how they would work in concert with each other and coordinate with systems such as ParABS.

In this work, we used a combination of high-resolution microscopy and genomic chromosome conformation capture (3C/Hi-C)^36^ to unveil the global organization of the diploid *Corynebacterium glutamicum* genome. *C. glutamicum* is a polar growing actinobacterium, whose genome encodes both SMC/ScpAB and MksBEFG. In this species, the two *oriC*s are continuously associated with the polar scaffold protein DivIVA, whereas newly replicated sister *oriC*s segregate towards division septa via the ParABS system^41–43^. In contrast to *B. subtilis*, *C. glutamicum* ParAB are by themselves crucially important drivers of reliable nucleoid separation prior to cell division, where ParAB deletions yield in 20% of anucleate cells^44–46^. Here, analyses of chromosomal ParB-binding patterns evince ten redundant *parS* sites, which mediate ParB subcluster formation at *oriC*. A single *parS* site maintains ParB propagation over 32 kb neighboring regions and is sufficient to promote the SMC-dependent alignment of the two chromosomal arms. Hi-C also reveals SMC-dependent long-range contacts surrounding *oriC*. In contrast to SMC, we show that the polar positioned MksBEFG condensin acts mostly on plasmid transmission to daughter cells, without obvious influence on nucleoid architecture.

## Results

### Chromosome segregation is governed by ten *oriC*-proximal *parS* sites

**Fig. 1:**
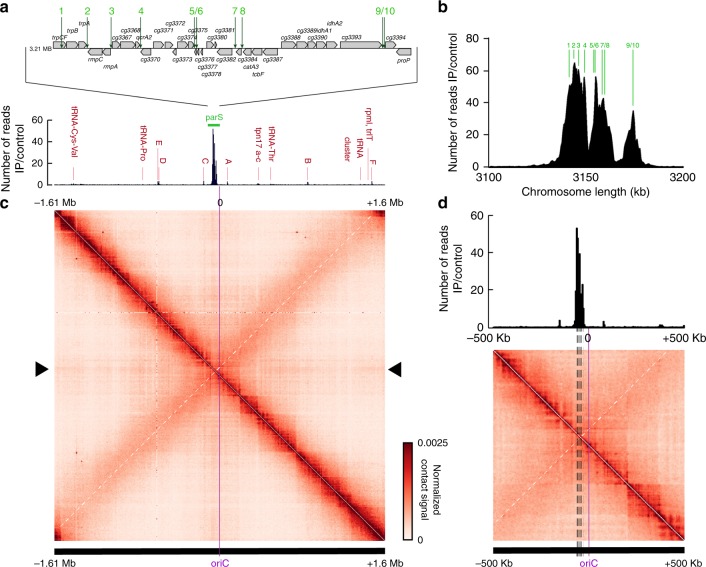
Chromosome organization hub at *oriC* domain in *C. glutamicum*. **a** Top: genomic region including ten *parS* sites of *C. glutamicum* with 16 bp consensus sequences. Below: ChIP-seq data on ParB-mCherry DNA-binding protein confirm *parS* sites shown above. Exponentially growing *C. glutamicum parB::parB-mCherry* cells (CBK006) were used for in vivo anti-mCherry ChIP-seq experiments. Shown is the ratio of ChIP signal relative to the input (fold-enrichment IP/control) in 5 Kb bins in linear scale along the chromosome with an *x*-axis centered at *oriC*. Red labels indicate minor enrichment signals at highly transcribed regions, such as rRNA operons (letters A–F). **b** ParB-ChIP-seq enrichment encompassing 3.1–3.2 Mb genomic region; *parS* sites 1–10 are indicated (green lines). **c** Normalized genomic contact map derived from asynchronously grown cells (fast growth, growth rate (*µ*) ≥ 0.6 h^−1^, exponential phase). *X*- and *Y*-axes indicate chromosomal coordinates binned in 5 Kb; *oriC*-centered (purple bar—coordinate 0). Color scales, indicated beside the contact map, reflect contact frequency between two genomic loci from white to red (rare to frequent contacts). White dashed line on the contact matrix indicate the mean signal of the secondary diagonal and black triangles on the side of the contact matrix indicate the “cross like” signal. **d** Structural chromosome organization of the *oriC* region. Magnification of contacts within 500 Kb surrounding *oriC*; *oriC* is indicated as a purple line and *parS* sites are indicated by dashed lines. ParB-enrichment zones at *parS* are shown above the contact map (ChIP signal relative to the input in 5 Kb bins). White dashed line on the contact matrix indicate the mean signal of the secondary diagonal.

Previous studies on *C. glutamicum* chromosome partitioning have revealed two stable ParB-*oriC* clusters at each cell pole, whereas newly replicated origins are segregated towards a division septum formed at midcell^41^. In *B. subtilis*, *C. crescentus* and *Pseudomonas aeruginosa* ParAB-mediated chromosome segregation and folding depends on *parS* sites^18,19,21^. In *C. glutamicum*, *parS* positions have not been characterized yet. Four to eight *parS* sites were predicted earlier in *Corynebacterineae*^5^. A BLAST analysis pointed at ten *B. subtilis*-like 16 bp consensus sequences in *C. glutamicum*, localized in one cluster within a 35 Kb region at 73 Kb from *oriC* (1% of the 3.21 Mb chromosome; Fig. 1a). Out of the ten *parS* sites, only the furthest from *oriC* (*parS1*) lies within a coding sequence (*trpCF*). All other *parS* sequences (labeled *parS 2-10*) were positioned within intergenic regions. Degenerated *parS* sequences with at least three base-pair mismatches were also identified further away from *oriC*, e.g., 5′ of *cg0146* or within the *fusA* and *cg1994* coding region. To test whether these putative *parS* were responsible for the recruitment of ParB, chromatin immunoprecipitation (ChIP) of ParB was performed with a strain harboring a mCherry-tagged version of the native ParB (note that all mutant strains used in this study derive from clean allelic replacements and have, unless otherwise noted, a wild-type-like phenotype). Distinct and very reproducible enrichment signals were obtained at the ten *parS* sites close to *oriC* (*parS*1-10 at 3.16 MB) (Fig. 1a), whereas the degenerated *parS* sequences failed to recruit ParB. Additional smaller peaks were identified at highly transcribed DNA regions, in particular at ribosomal genes, transfer RNA gene clusters, and at all of the ribosomal RNA operons (Fig. 1a). Magnification of the *oriC* region reveals three distinct ParB propagation zones overlapping with *parS*1-4, *parS*5-8, and *parS*9-10, respectively (Fig. 1b). Remarkably, those three regions seem to recruit decreasing amounts of ParB, from *parS1-4* (most enriched) to *parS9-10* (less enriched). As all *parS* are identical in sequence, differences in ParB recruitment might result from the number and distance of *parS* sequences in the context of the overall nucleoid folding patterns at the *oriC* region.

### Higher-order organization of the *C. glutamicum* chromosome

In *B. subtilis*, SMC-mediated chromosome folding initiates at ParB-*parS* clusters surrounding the *oriC*, bridging the two replichores with each other^10,21^. To characterize whether *C. glutamicum parS* sites play a similar role in the overall organization of the chromosome, we applied a Hi-C-like approach^10,47^ to exponentially growing wild-type cells (Methods). The genome-wide contact map, displaying the average contact frequencies between all 5 Kb segments of wild-type chromosomes (Fig. 1c) displayed the following 3D features. First, a strong and broad diagonal reflecting frequent local contacts between adjacent loci and observed in all Hi-C experiments. Second, chromatin interaction domains (CIDs), i.e., self-interacting regions previously described in *C. crescentus* and other species^10,21,36,38,48^ (Fig. 1c and Supplementary Fig. 1) (11 domains detected at a 200 Kb resolution). In *C. glutamicum*, 6 out of 11 boundaries are associated with high transcriptional activity or gene lengths (Supplementary Fig. 1A). The Hi-C signal did not present overall a clear correlation with transcriptional activity (Supplementary Fig. 1B). Other roadblocks like nucleoid-associated proteins might play a major role in confining chromosomal interaction domains. Third, a secondary diagonal perpendicular to the main one and extending from the *ori*-proximal, 35 Kb *parS* cluster (Fig. 1d, white dashed line) down to the replication terminus. This structure shows that the two replichores are bridged over their entire length, as in *B. subtilis*^10,21,36^. Interestingly, this secondary diagonal also displays discrete, long-range contact enrichments (Fig. 1c), which may reflect bridging of the two chromosomal arms at specific locations. Finally, the contact map also displays a faint, cross-shaped signal corresponding to contacts between the *ori* region and the rest of the chromosome (Fig. 1c, dark triangle on the sides of the contact matrix), as in *B. subtilis*^21^. These contacts might be due to the segregation and translocation of the ParB-*oriC* complex along the nucleoid during segregation when *oriCs* reposition at midcell. This signal is also maximal at the *parS* cluster and not at *oriC* locus (Fig. 1c, d). This observation reinforces the fact that the *parS* cluster is at the tip of *Corynebacterium* chromosome fold and is one of the main actors of chromosome segregation. A similarity matrix between the different constructed Hi-C matrices was calculated (Supplementary Fig. 2A) and flow cytometry was performed with all samples used for Hi-C analyses to control for chromosome number and potential replication differences (Supplementary Fig. 2B–D).

### A single *parS* site is sufficient to maintain chromosome architecture

**Fig. 2:**
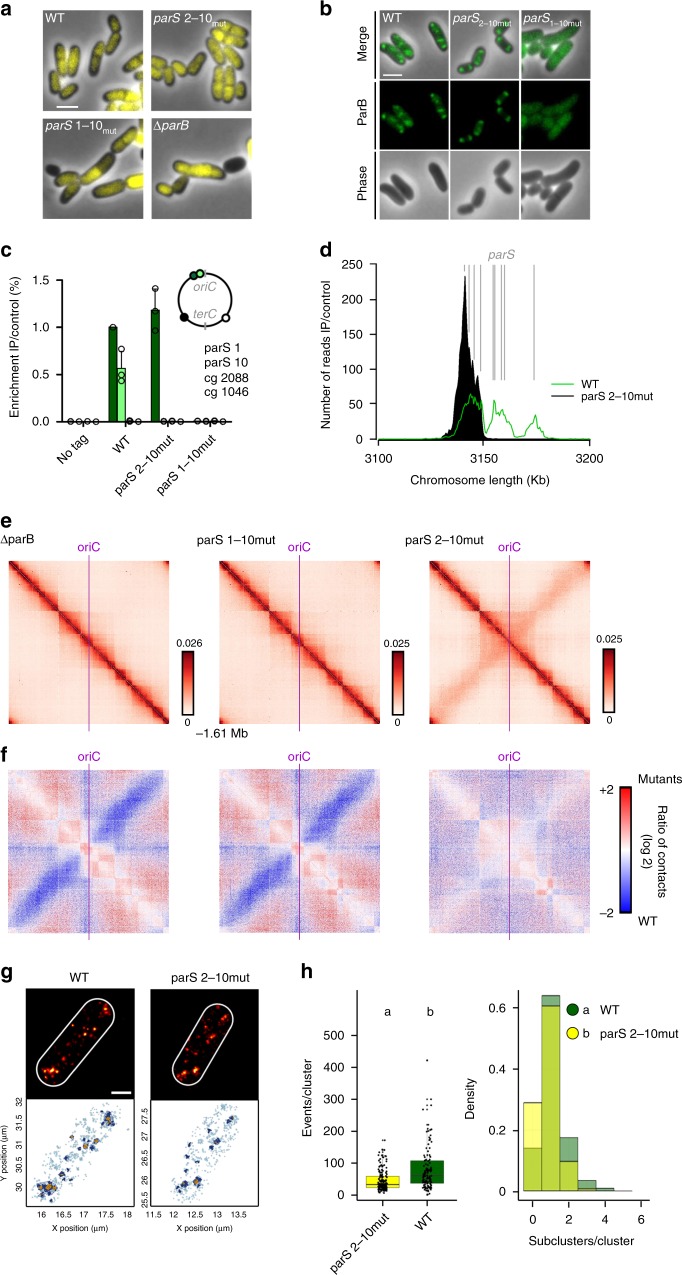
A single *parS* site mediates chromosome folding. **a** One *parS* site is necessary and sufficient for wild type-like morphology and nucleoid segregation. Phase-contrast images of exponentially grown cells harboring either all (WT), one (*parS*_2-10mut_, CBK023), or none (*parS*_1-10mut_, CBK024) *parS* site(s), or lacking *parB* (Δ*parB*, CDC003) are shown. DNA is stained with Hoechst (yellow). Scale bar, 2 µm. **b** Fluorescence microscopy analysis of *parB*∷*parB-mCherry* (shown in green) in wild type (CBK006), *parS*_2-10mut_ (CBK027), and *parS*_1-10mut_ backgrounds (CBK028). Absence of *parS* leads to diffuse cellular ParB localizations. Scale bar, 2 µm. **c** ChIP-qPCR for strains described before, normalized to wild-type *parS*1 signal (mean + SD, *n* = 3). **d** ChIP-seq of *C. glutamicum parB*∷*parB-mCherry parS*_2-10mut_ (black) at a 3.1–3.2 Mb chromosomal range. Wild-type-like propagation (green) of ParB protein around *parS1-4*; 0.5 Kb bin size. Location of *parS* sites present in wild type or mutant sequences are indicated (gray lines). **e** Normalized contact maps of Δ*parB*, *parS*_1-10mut_, and *parS*_2-10mut_ mutants centered at *oriC* (CDC003, CBK024, and CBK023). Color codes as in Fig. 1. **f** Differential maps correspond to the log2 of the ratio (wild-type norm/mutant norm); color scales indicate contact enrichment in mutant (blue) or wild type (red) (white indicates no differences between the two conditions). **g** Single-molecule localization microscopy of representative wild-type and *parS*_2-10mut_ cells (CBK009 and CBK029). Top: Gaussian rendering of ParB-PAmCherry signals (0.71 PSF, 1 px = 10 nm), below: color-coded representation of ParB-PAmCherry events within corresponding cells^49^; all events (light blue), macroclusters (dark blue) and subclusters (yellow) are indicated. Scale bar, 0.5 µm. See Methods and Supplementary Fig. 7 for details. **h** Comparison of ParB-PAmCherry cluster properties. Only the two biggest clusters per cell were taken into account for analyses; significant differences between conditions are indicated by small letters above datasets. Left: events per macrocluster, medians are indicated as solid lines, and whiskers mark 1.5 IQRs (interquartile ranges); clusters_wild type_: *n* = 130, clusters_*parS*2-10mut_: *n* = 143. Right: subcluster numbers per macrocluster shown as overlay bar chart for both strains. Number of subcluster per macrocluster (two-tailed Kruskal–Wallis rank-sum test: *χ*^2^ = 12.284, df = 1, *p* = 0.0004569) and macroclusters size (two-tailed Kruskal–Wallis rank-sum test: *χ*^2^ = 27.582, df = 1, *p* = 1.506e − 07) differ significantly between. Source data are provided as a Source Data file.

As all *parS* sites are in close proximity on the *C. glutamicum* chromosome, we tested the importance of ParB-*parS* complex titration for the overall chromosome organization. Cells with chromosomes carrying the single *parS1* site (for *parS* mutations, see Supplementary Fig. 3A) grow and divide like wild-type cells (Supplementary Figs. 2A and 3B). However, the removal of all ten *parS* sites resulted in a cell length phenotype (Supplementary Fig. 3B) and 29% DNA-free mini-cells, hinting to a nucleoid segregation defect similar to the Δ*parB* phenotype (Fig. 2a and Supplementary Table 1). We further analyzed ParB localization in mutant strains carrying either a single or no *parS* site. First, if only *parS1* is present, cellular localization of fluorescent ParB-mCherry foci is similar to wild type, positioning at cell poles and migrating to the newly formed septa^41^ (Fig. 2b). Interestingly, the combination of a single *parS* site with ParB-eYFP resulted in 7% anucleate mini-cells (Supplementary Fig. 3C and Supplementary Table 1), reflecting functional constraints of the ParB-eYFP fusion in the presence of only one *parS* site. Therefore, the high number of chromosomal *parS* sites likely evolved to improve the robustness of the segregation machinery. ParB ChIP-quantitative PCR (qPCR) signals of locus *parS1* were similar in both wild-type and mutant strains (Fig. 2c). ParB spreading around the single *parS* site was characterized through ChIP-seq analysis (Fig. 2d and Supplementary Fig. 4), where ParB binding was maximum within 2 Kb windows on both sides of *parS*, while extending up to 16 Kb on either side. However, redundancy of *parS* sites is not restricted to *parS*1 in *C. glutamicum*, as exemplified by the analysis of the single *parS*10, which was equally sufficient for wild-type-like growth and morphology (Supplementary Fig. 3D–E). A single *parS*10 site recruits ParB exclusively within the third nucleation zone encompassing 26 Kb (Supplementary Fig. 3A, F). We next investigated the role of *parS* sites and ParB in the overall chromosome folding by performing Hi-C in mutants (Fig. 2e, f). The absence of either ParB or of all *parS* sites led to the disappearance of the secondary diagonal. In addition, the cross-shaped pattern reflecting contacts between the *ori* and the whole chromosome disappears in those mutants, also illustrated by the ratio between wild-type and mutant contact maps (Fig. 2f). This result shows that *parS* sites and ParB are two major structural components of chromosome organization and act in the same pathway to recruit downstream factors that fold the chromosome emanating from the *parS* cluster, and bridge the two chromosomal arms together down to the replication terminus region. The contact map of the strain deleted for *parS2-10*, but carrying a single *parS1*, maintains a secondary diagonal, showing that *parS1* alone is sufficient to ensure the loading of ParB and the overall folding of the chromosome (Fig. 2e, f). However, some differences appeared between the wild type and the single *parS1* site contact maps. In the mutant, the large domain surrounding the *oriC* shows minor differences in the contact maps compared with wild type, suggesting that a single *parS* site is not sufficient to fully restore the complexity of *Corynebacterium* chromosome *ori* folding (Fig. 2e, f).

The single *parS* site was then repositioned at different genomic regions. Cells harboring an ectopic, single *parS* site at 9.5°, 90°, 180°, or 270° positions were viable (Supplementary Fig. 5A–C). Unlike cells harboring *parS*1 at its original position, ParB-*parS* complexes distribute virtually randomly along the longitudinal cell axis in all of these mutants (Supplementary Fig. 5C), resulting in ~25% anucleate cells (Supplementary Table 1). Therefore, *parS* shifts result in nucleoid segregation defects. The number of ParB foci nevertheless correlates well with cell length (Supplementary Fig. 5D), excluding replication initiation deficiencies. ParB binding to a *parS* sequence positioned at the 90° chromosomal position (locus *cg0904*, strain CBK042) was identified in a 9 Kb range on either side of *parS* (Supplementary Figs. 4A, 5E), approximately half the ParB-propagation distance determined for cells harboring one *parS* at its native locus. We also analyzed the mutant harboring *parS* at 90° chromosomal position by Hi-C (CBK037). The contact map of this mutant displays a “bow shape” or a hairpin motif at the position of the aberrant *parS* sequence (Supplementary Fig. 6), reminiscent to the one observed in *B. subtilis* at the level of the ori-distal *parS* site and pointing a local folding of the chromosome (Supplementary Fig. 6A). Collectively, these results show a redundancy of *parS* sites, with an optimal function confined to the *oriC*-proximal region.

### PALM identifies ParB subclusters

To directly characterize *oriC* domain compaction via ParB, we applied photoactivated localization microscopy (PALM) to visualize individual ParB-PAmCherry molecules with nanometer resolution (~20 nm localization precision). PALM revealed distinct ParB-dense regions at cell poles and quarter position regions, similar to foci observed via diffraction-limited epifluorescence microscopy (Fig. 2g). These ParB-enriched regions (macroclusters) display heterogeneous densities, with a variable number of higher density zones within subclusters. Macro- and subclusters have been identified via the OPTICS algorithm that orders data points according to their spatially closest neighbors for identification of clustering structures^49,50^ (see Methods and Supplementary Fig. 7A) and analyzed in strains harboring a single, two, or all the *parS* sites (Fig. 2g and Supplementary Fig. 7B). We define a macrocluster as 32 individual events being localized within a maximum distance of 50 nm for macroclusters and 35 nm for subclusters, yielding in cluster numbers that are in line with ParB epifluorescence data and *oriC* numbers determined by flow cytometry (Supplementary Table 2). It is noteworthy that high chromosome numbers promote inter-molecular *oriC* colocalization in fast-growing cells. For more accurate cluster estimations, PALM analysis was performed using slow-growing cells resulting in significantly fewer ParB macroclusters per cell (Supplementary Fig. 7C)^41^. As segregation of *oriC* complexes might alter their DNA compaction, we focused on the two largest macroclusters per cell, stably tethered at cell poles. Although this is not a direct measurement of the number of ParB nucleation points (*parS*), a strain with a higher number of *parS* sites can be expected to result in higher ParB density variability when compared with one which contain a single or no nucleation point. The amount of ParB contained within each macrocluster in wild type is significantly higher than in cells containing the single *parS1* site (Fig. 2h), in agreement with the ParB deposition observed via ChIP-seq. A parallel between PALM and ChIP-seq can also be drawn with respect to the number of subclusters per macrocluster, with a higher number of subclusters in the wild type that accordingly harbors three ParB nucleation zones along the *parS* cluster compared with the single *parS* site forming only one zone (Fig. 2h). Absence of all *parS* sites likewise results in a significant reduction of ParB macrocluster size and subcluster numbers compared with wild type (Supplementary Fig. 7D). These differences were not observed when comparing cells harboring all or two *parS* sites (*parS*1,10), which harbors two distinct ParB nucleation regions surrounding *parS*1 and *parS*10 (Supplementary Figs. 4A,  5F, and 7E). These observations could explain the differences observed between contact matrices of wild type and ∆*parS* 2–10 strains, and the higher structuring of the *oriC* domain when only one *parS1* site is present. We therefore conclude that the architecture of the *C. glutamicum* partition complex is dependent on *parS*, and that ParB-*parS* nucleoprotein complexes are visible as individual subclusters.

### *C*. *glutamicum* harbors two paralogs of condensin complexes

**Fig. 3:**
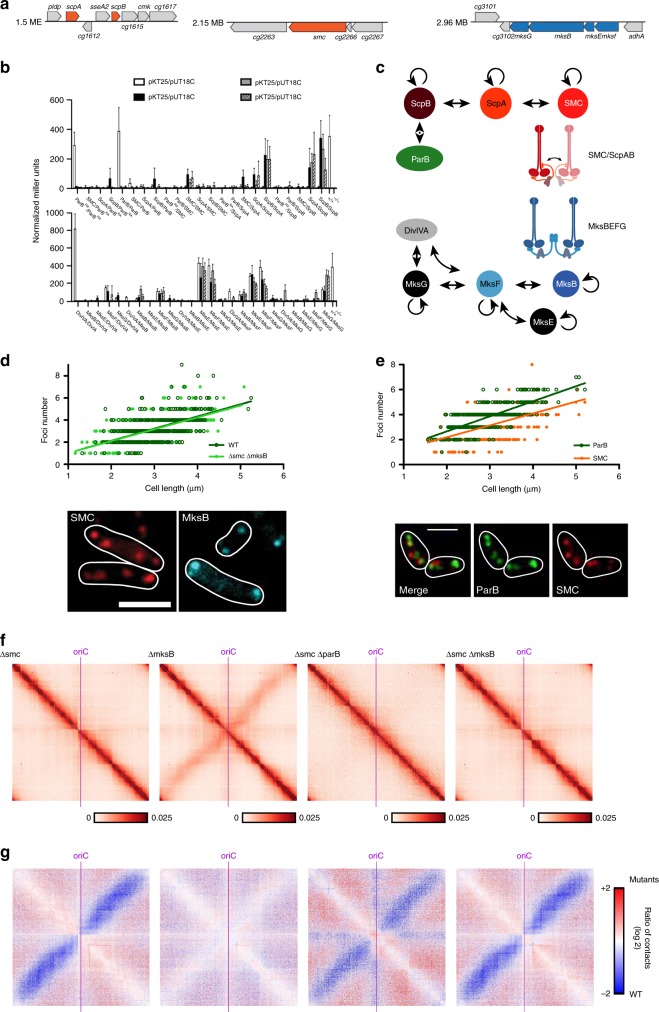
Functional characterization of two SMC-like complexes in *C. glutamicum*. **a** Sections of the *C. glutamicum* genome map indicating localizations of condensin subunit genes. **b** Confirmation of protein–protein interactions via bacterial two-hybrid screen. Interactions were quantified by β-galactosidase assays in all combinations of hybrid proteins: C/C- (18C/T25), N/C- (18/T25), C/N- (18C/NT25), and N/N- (18/NT25) terminal fusions of adenylate cyclase fragments, ParB^RA^: ParB mutant R175A (mean ± SD, *n* = 3). **c** Illustration of SMC/ScpAB and MksBEFG subunit interactions based on bacterial two-hybrid data; cartoons indicate condensin complex formations. **d** Top: dependence of ParB foci numbers on cell length in *C. glutamicum* wild type (WT) and Δ*smc* Δ*mksB* (ΔΔ, CBK011) cells grown in BHI (*n* > 350). Linear regression lines are shown *r*(WT) = 0.57, *r*(ΔΔ) = 0.62; slopes and intercepts are equal (ANCOVA, F(1, 770) = 0.059, *p* = 0.808; ANCOVA, F(1, 771) = 0.60, *p* = 0.4391). Below: cellular localization of condensin subunits in *C. glutamicum smc*∷*smc-mCherry* and *mksB*∷*mksB-mCherry* cells (CBK012, CBK015). Microscopy images exemplify cellular mCherry fluorescence of SMC (left) and MksB (right); white lines indicate cell outlines. Scale bar, 2 µm. **e** Top: SMC and ParB foci numbers positively correlate with cell length in double labeled strain *smc*∷*smc-mCherry parB*∷*parB-mNeonGreen* (CBK013), *r*(ParB) = 0.74, *r*(SMC) = 0.53 (*n* > 350). Below: subcellular localization of ParB and SMC is exemplified in representative cells shown in overlays between mNeonGreen and mCherry fluorescence, and in separate channels. Scale bar, 2 µm. **f** Normalized contact maps of Δ*smc*, Δ*mksB*, Δ*parB*/Δ*smc*, and Δ*smc*/Δ*mksB* mutants (CDC026, CBK001, CBK002, and CBK004), displayed as in Fig. 1. **g** Corresponding differential maps between WT and mutant contact maps, indicating the log of the ratio (wild-type norm/mutant norm) are presented as in Fig. 2. Source data are provided as a Source Data file.

In bacteria, the condensin paralog complexes SMC/ScpAB and, in *E. coli* and other enterobacteria, MukBEF, are key factors of chromosome folding^10,21,36,38^. MksBEF (for MukBEF-like SMC) is another condensin occasionally found in bacterial genomes^40^, whose role(s) remain(s) obscure. A sequence homology search of the *C. glutamicum* genome pointed at the presence of both SMC/ScpAB and MksBEF. The SMC/kleisin is encoded by genes *cg2265* (*smc*), *cg1611* (*scpA*), and *cg1614* (*scpB*) (Fig. 3a), whereas the Mks complex is encoded on a widely conserved operon^40^ and comprises genes *cg3103*–*cg3106* (*mksGBEF*) (Fig. 3a), including MksG, which was being suggested to act in complex with MksBEF^40^.

To characterize condensin complex formation in vivo, mass spectrometry of anti-mCherry pulldown experiments using SMC-mCherry and MksB-mCherry as baits of whole-cell lysates were performed. Wild-type-like growth of corresponding strains and stability of SMC and MksB fluorescent fusions were confirmed (Supplementary Fig. 8A, C). Kleisin subunit ScpA and ScpB co-precipitated significantly with SMC compared with the negative control containing free mCherry, whereas subunits MksF and MksE, but not MksG, were substantially enriched in the MksB pulldown experiments (Supplementary Fig. 8D). ParB, which mediates SMC loading onto DNA in *B. subtilis* and *S. pneumonia*^20,34,35^, was not immunoprecipitated with SMC in any of the experiments. Bacterial two-hybrid analyses confirmed mass spectrometry results, pointing at the formation of SMC/ScpAB and MksBEF complex (Fig. 3b, c). No significant interactions between SMC/ScpAB and ParB were detected, and we observed ScpA–ScpA self-interaction signals well above background. Moreover, MksG connects to the MksBEF complex via interaction with MksF, whereas the condensin complex subunits MksF and MksG further interact with the *C. glutamicum* polar scaffold protein DivIVA. Proteomics analysis failed to identify DivIVA in any immunoprecipitation. A possible reason for this is the high degree of DivIVA oligomerization by molecular bridging at cell membranes^51,52^.

### SMC-mediated cohesion of chromosomal arms

We aimed to characterize *C. glutamicum* condensin SMC/ScpAB. Mutation of the SMC/ScpAB complex causes a conditionally lethal phenotype due to chromosome mis-segregation in *B. subtilis*^25^. In sharp contrast, a *smc* deletion in *C. glutamicum* did not result in either growth defects, DNA-segregation defects, or aberrant cell length distributions and morphologies compared with the wild type in minimal or complex media (Supplementary Fig. 9A, B and Supplementary Table 1). Nonetheless, the combination of genetic backgrounds *parB∷parB-eYFP* and Δ*smc* yield a minor fraction of anucleate cells (4–5%) (Supplementary Fig. 9A, C and Supplementary Table 1), indicating that SMC and ParB function in the same pathway and with ParB being epistatic to SMC. Hence, a functional interaction of SMC and ParB proteins regulating chromosome organization is likely. To further determine cellular localization of SMC/ScpAB complexes, a strain harboring a fluorescently tagged version of core subunit SMC was imaged, revealing the formation of SMC clusters along the entire longitudinal axis of the cell (Fig. 3d). Clusters of SMC and ParB investigated in a strain carrying both labeled complexes (*parB∷parB-mNeonGreen smc∷smc-mCherry*) are often proximal but do not always colocalize, whereas the foci numbers correlate with cell length (Fig. 3e). Up to eight SMC-mCherry foci were counted per cell. On average, cells contained fewer SMC-foci than ParB nucleoprotein complexes (Supplementary Fig. 8B). To further characterize the role of SMC, we generated Hi-C contact maps of the mutant (Fig. 3f). Deletion of *smc* abolishes the secondary diagonal in the maps (Fig. 3f), indicating that SMC and ParB function in the same pathway and have a synthetic phenotype concerning the cohesion of the two chromosomal arms. The combination of *smc* and *parB* mutations mimics a *parB* phenotype (Supplementary Fig. S9A, D), again resulting in the loss of contacts between chromosomal arms and further in the loss of the segregation signal described before (Fig. 3f, g). However, ∆*parB* and ∆*smc* contact maps display different patterns along the main diagonal, suggesting that those two proteins affect differently chromosome architecture of *C. glutamicum* (Supplementary Fig. 9E). Therefore, it appears that an interplay of SMC/ScpAB with ParB is responsible for replichore cohesion in *C. glutamicum*, similar to *B. subtilis* and *C. crescentus* each harboring only one type of condensin complex^10,21,22,36^.

### ParB-dependent SMC recruitment to chromosomal loading sites

**Fig. 4:**
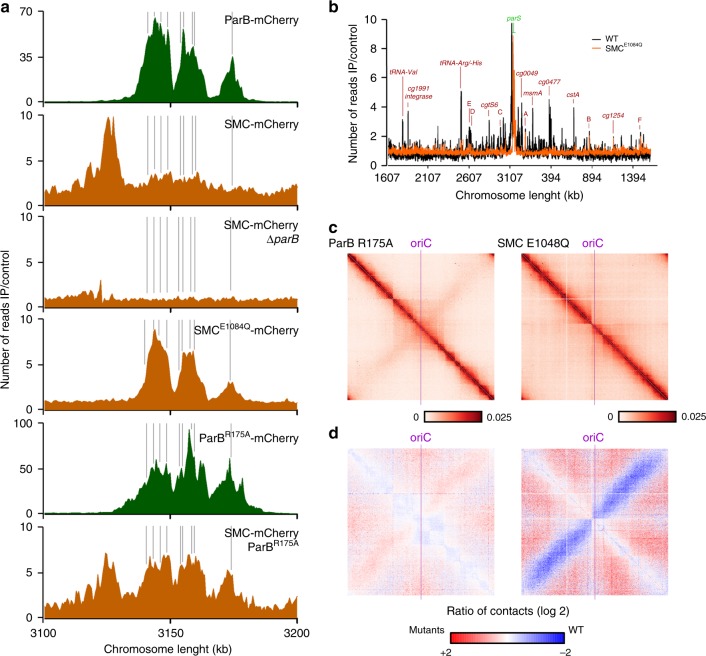
Chromosomal SMC loading is mediated by ParB at *parS* sites. **a** SMC enrichment at *parS* sites (gray) is ParB-dependent. ChIP-seq of ParB-mCherry (green; CBK006 and CBK047) and SMC-mCherry (orange; CBK012, CBK014, CBK051, and CBK049) in strain backgrounds as indicated. Depicted are chromosomal ranges of 3.1–3.2 Mb, bin size 0.5 Kb. **b** Whole-genome ChIP-seq data of strains harboring SMC-mCherry wild type (gray, CBK012) or E1084Q mutant (orange, CBK050). SMC enrichment at *parS* sites and at other loci (red letters), in particular tRNA gene clusters and at rRNA genes (**a**–**f**) is illustrated in 0.5 Kb bins in linear scale along the chromosome with an *x*-axis centered at *oriC*. **c** Normalized contact map of mutant strains *parB*∷*parB*^R175A^ (CBK047) and **d** the corresponding differential map indicating the log of the ratio (wild-type norm/mutant norm) as in Fig. 2.

As cellular SMC-mCherry signal hinted to distinct agglomeration clusters along the *C. glutamicum* chromosome, we investigated its putative binding sites via ChIP-seq. A small enrichment in SMC deposition was detected at and around the *parS*1-10 cluster (Fig. 4a), which disappears upon *parB* or *parS* deletion (Fig. 4a and Supplementary Figs. 4 and 10A, B). In addition, comparably minor enrichment signals are present throughout the chromosome, which partially coincide with genomic loci of high transcriptional activity. Distinct SMC-mCherry foci are less frequent in the absence of ParB or *parS* (Supplementary Fig. 10C). These findings suggest that ParB promote condensin loading onto DNA at *oriC*-proximal *parS* sites. In addition, ChIP-seq revealed that SMC concentrates at a 13 Kb region upstream *parS*1 (Fig. 4a). SMC enrichment in this region was lost following a partial deletion of this locus and its reinsertion at another genomic position (Supplementary Fig. 10D-F) or following its substitution by a random DNA sequence (Supplementary Fig. 10D, G). Therefore, the accumulation of SMC at the 13 Kb region in the vicinity of *parS* sites points at roadblocks that trap SMC rather than specific SMC binding. This hypothesis is further supported by the study of the contact map of wild type cells (Fig. 1c, d and Supplementary Figs. 1 and 11). Indeed, the SMC enrichment region is clearly delimited by a strong border on its left (Supplementary Fig. 1, Directional Index at 100 Kb resolution and Supplementary Fig. 11, red dashed line). In the absence of ParB or SMC (Supplementary Fig. 11), the strong border observed in Hi-C maps is shifted towards *parS* sites. Therefore, this border originates from a combination of multiple processes.

SMC is also recruited to *parS* inserted in ectopic positions, e.g., the 90° *parS*-insertion (Supplementary Figs. 5, 6). Indeed, in the absence of SMC (Supplementary Fig. 6), the bow-shape motif is no longer present at the ectopic *parS* site, demonstrating that chromosomal arm cohesion is SMC-dependent, and that artificial loading of SMC at non-native positions is not sufficient to fold the entire chromosome. We further assayed chromosomal SMC-loading sites by making use of a well-characterized SMC ATP-hydrolysis mutant E1084Q^32,53–55^. SMC^E1084Q^ mutant strongly accumulates at *parS* sites in *C. glutamicum*, mimicking a ParB-enrichment pattern (Fig. 4a). Decreased ChIP-enrichment signals throughout the rest of the chromosome hint to an impaired SMC migration along the DNA (Fig. 4b). Moreover, the Hi-C contact map of SMC^E1084Q^ clearly demonstrates that this mutant is no longer able to bridge chromosomal arms (Fig. 4c, d). Conclusively, we confirm specific SMC loading by ParB to an *oriC*-proximal region on the *C. glutamicum* chromosome.

Interestingly, ChIP analysis of a *C. glutamicum* ParB^R175A^ mutation, which leads to a loss of dimer–dimer interactions in the corresponding *B. subtilis* ParB^R79A^ mutation^8^, results in increased SMC binding at ParB^R175A^ propagation zones (Fig. 4a). Changes in in-vitro double-stranded DNA-binding affinities compared with wild-type ParB could not be verified (Supplementary Fig. 12), neither enhanced binding affinity for SMC/ScpAB by bacterial two-hybrid analyses (Fig. 3b). The mutation results in large fractions of DNA-free cells, and growth rates and ParB^R175A^ cluster formation are particularly affected in cells harboring a single *parS* site (Supplementary Table 1 and Supplementary Fig. 12). ChIP-data indicate broadened and less distinct enrichment signals compared with wild-type ParB in the presence of all or one *parS* sites (Fig. 4a and Supplementary Figs. 4 and 12D, E). Therefore, ParB^R175A^ is still capable of building up weak nucleoprotein complexes around *parS* sites. Hi-C data of the corresponding mutant show the same tendency with a conservation of the overall chromosome architecture with the presence of a secondary diagonal and the conservation of the origin domain folding (Fig. 4c and Supplementary Fig. 11). However, the signal emanating from the secondary diagonal is weak compared with the wild-type one as shown by the ratio matrix (Fig. 4c, d). Consequently, SMC translocation along the DNA appears only partially impaired in this mutant (Fig. 4a and Supplementary Fig. 4). The ParB^R175A^ mutation either locks the translocation ability of SMC/ScpAB by a direct interaction or alterations of ParB^R175A^ nucleoprotein complex properties, namely an incorrect folding of the *oriC* domain leads to SMC trapping along DNA loops at *parS*. Altogether, these analyses confirm that the *C. glutamicum* SMC/ScpAB complex is a *Bacillus*-like condensin that loads and redistributes to distant chromosomal regions via an explicit ParB interaction at *parS*.

### MksB impacts on plasmid maintenance in *C. glutamicum*

**Fig. 5:**
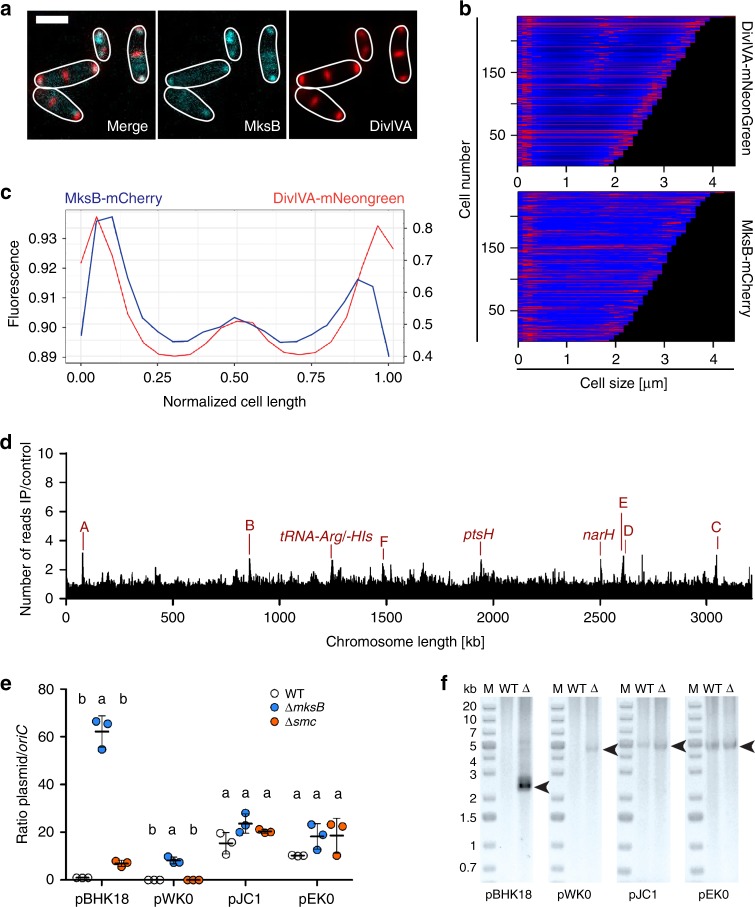
MksB localizes with DivIVA and impacts on plasmid copy numbers. **a** Epifluorescence microscopy images of CBK092 cells; MksB-mCherry (cyan) and DivIVA-mNeonGreen fluorescence (red) are shown as overlay and in separate channels; cell outlines are indicated by white lines. Scale bar, 2 µm. **b** Demographs show fluorescence profiles of DivIVA and MksB in strain CBK092 along cell axes sorted by length. Fluorescence intensities are illustrated relative to maximal intensity values per cell by a color gradient ranging from dark blue (low intensities) to red (high intensities). **c** Averaged fluorescence (a.u.) of MksB-mCherry (blue) and DivIVA-mNeonGreen (red) along the longitudinal cell axis of CBK092 cells (*n* > 200 cells). Fluorescence values of profiles were normalized in length and fluorescence intensity per cell. The resulting values were then binned (bin = 0.05 µm). **d** Anti-mCherry-ChIP-seq analysis of *mksB*∷*mksB-mCherry* strain CBK015 as described in Supplementary Fig. 4. **e** Plasmid copy numbers of low-copy (pBHK18 and pWK0) and high-copy number vectors (pJC1 and pEC0) relative to *oriC* numbers per cell, assayed by qPCR. Ratios were compared between *C. glutamicum* wild type, Δ*mksB*, and Δ*smc* mutant cells grown in BHI medium without addition of plasmid selection antibiotic after overnight pre-incubation with antibiotic (mean ± SD, *n* = 3). One-way ANOVAs yielded significant variations among strains harboring pBHK18 (F(2, 6) = 233.3, *p* = 2.05e − 06) and pWK0 (F(2, 6) = 98.66, *p* = 2.57e − 05), but not among strains harboring pEK0 (F(2, 6) = 2.496, *p* = 0.163) and pJC1 plasmids (F(2, 6) = 51.75, *p* = 0.0739). Letters indicate significant differences between data sets determined by post-hoc Bonferroni analysis at *p* < .05. **f** Plasmids named in **a** were extracted from *C. glutamicum* wild type and *mksB* deletion strains grown in BHI medium including selection antibiotic, visualization of extracted DNA on 1% agarose gels (corresponds to yield from ~1 × 10^9^ cells each). Arrows indicate size of plasmid DNA. Source data are provided as a Source Data file.

To test whether both *C. glutamicum* condensins SMC and MksB are redundant in function, we generated mutants lacking the condensin core subunit Δ*mksB* or both Δ*smc* Δ*mksB*. Similar to Δ*smc*, no growth and morphology phenotypes could be detected for both mutants (Supplementary Fig. 9A, D and Supplementary Table 1). A triple mutation Δ*parB* Δ*smc* Δ*mksB* did not aggravate the Δ*parB* phenotype, excluding redundancy of condensin functions in chromosome segregation (Supplementary Fig. 9D). Further, *oriC*-ParB foci numbers (Fig. 3d) and their spatiotemporal localization (Supplementary Fig. 9A) remain largely unaffected upon deletion of *smc* and *mksB*. MksB fluorescence was mainly detected at the cell poles (Fig. 3d), further supporting an interaction with the polar protein DivIVA. To test cellular MksB-DivIVA colocalization in more detail, we constructed a dual-reporter strain harboring MksB-mCherry in combination with DivIVA-mNeonGreen, which grows and divides in comparasion with the wild type (Fig. 5a, Supplementary Fig. 13A, and Supplementary Table 1). Individual protein fluorescence patterns of MksB and DivIVA are displayed in large-scale demograph analyses (Fig. 5b). Averaged fluorescence profiles along longitudinal cell axes extracted from still microscopy images show colocalization of MksB and DivIVA at cell poles and division septa prior to cytokinesis in long cells (Fig. 5b, c) even if cellular MksB fluorescence intensities are low compared with DivIVA. The relative localization of MksB and DivIVA has also been observed via PALM microscopy. Here we can see that the MksB foci composed of the highest number of localizations typically localize at the poles and are surrounded by DivIVA itself (Supplementary Fig. 13B). Although no quantitative analysis has been performed, the number of visible foci in the imaged cells does not differ with what has been already observed via conventional fluorescence microscopy (Fig. 5a). Moreover, we applied Hi-C to characterize the role of MksB in genome folding in the different mutants (Fig. 3f). In contrast to *smc*, deletion of *mksB* had no effect at large scale on chromosome organization, as shown by the ratio map between the wild type and the mutant (Fig. 3g). Moreover, ∆*smc* and ∆*smc*∆*mksB* contact maps were nearly identical (Fig. 3f), showing that MksB and SMC are most likely not involved in the same process(es). Finally, we applied the software HiCRep on our various Hi-C map, a framework for assessing the reproducibility of Hi-C data^56^ (Supplementary Fig. 2A). Strain backgrounds ∆*parB*, ∆*parS*, and ∆*parB*∆*smc* Hi-C maps appear to form a first cluster; ∆*smc* and ∆*smc*∆*mksB* appear to form a second cluster; finally, wild type, ∆*parS*2-10, and ∆*mksB* form a third cluster. This result strongly suggests that MksB does not significantly affect chromosome architecture in *C. glutamicum*. ChIP-seq of MksB failed to detect specific loading sites along the *C. glutamicum* chromosome (Fig. 5d), supporting the hypothesis that MksB, unlike other bacterial condensins studied so far, plays no direct or indirect role in *C. glutamicum* chromosome organization. Therefore, we analyzed its impact on the maintenance of extrachromosomal DNA. The MksBEFG complex appears involved in plasmid maintenance, as shown by the qPCR copy number analysis of two low-copy number (pBHK18 and pWK0) and two high-copy number (pJC1 and pEK0) *E. coli*–*C. glutamicum* shuttle vectors sized 3.5–6 Kb. High-copy number plasmids derive from cryptic *C. glutamicum* plasmids^57,58^, whereas replicons of both low-copy number plasmids originate from a plasmid isolated from the closely related *Corynebacterium diphteriae*^59,60^. In Δ*mksB* mutants, both low-copy number plasmids pBHK18 and pWK0 were enriched 60- and 10-fold compared with wild type, when grown in the absence of selection marker (Fig. 5e). On the contrary, the amount of high-copy number vectors per cell was hardly affected. A Δ*smc* control did not result in a significant increase of plasmid levels compared with wild type (Fig. 5e). We confirmed these findings by plasmid extractions from *C. glutamicum* cells lacking MksB that yielded exceptionally large quantities of pBHK18 and pWK0, turning them into high-copy number plasmids under these conditions (Fig. 5f). By contrast, amounts of pJC1 and pEK0 did not differ notably compared with control strains. These analyses show a MksB-dependent decrease in plasmid level, specifically of low-copy number plasmids. Notably, we observed emerging susceptibility of cells towards the pBHK18 selection antibiotic in the absence of MksB when testing its stability in plating assays and, therefore, cannot exclude side effects of MksB on the expression of antibiotic resistance. Subcellular MksB-mCherry localization was further assessed in the absence and presence of pEK0 or pWK0. The presence of plasmids does not have an impact on cell growth and morphology (Supplementary Fig. 13C) and wild-type-like MksB foci numbers per cell were detected (Supplementary Fig. 13D). Cellular MksB fluorescence profiles were further extracted (Supplementary Fig. 13E) and sorted by cell length in demographs showing MksB localization at cell poles and frequently at midcell prior to cytokinesis for all conditions (Supplementary Fig. 13E, F). However, polar MksB fluorescence tends to be more defined in cell populations harboring plasmids (Supplementary Fig. 13E, F).

Altogether, our data show that the two condensins in *C. glutamicum* evolved very different functions: whereas SMC/ScpAB act with ParB to promote replichore pairing and origin domain organization, MksBEFG does not organize chromosome architecture and seems involved in plasmid maintenance through a mechanism that remains to be characterized.

## Discussion

Condensins are widely conserved enzyme machineries, which have been implicated in chromosome organization of pro- and eukaryotes^61^. For long, it was considered that bacterial genomes encode one condensin complex that would either be of the Smc/ScpAB type as found in *B. subtilis* and *C. crescentus* or the MukBEF complex encoded in *E. coli* and related proteobacteria^23^. However, recent reports suggested the existence of two or even multiple condensin systems in a single species^40^. Yet, the underlying mechanisms and the precise function of these two condensin systems remained largely untested. We report here that the Gram-positive actinobacterium *C. glutamicum* also contains SMC/ScpAB and the Muk-like MksBEFG complexes. We set out to address the individual functions of the two condensin systems. Surprisingly our data provide clear evidence that the class of MksBEFG proteins do not work as chromosomal interactors; thus, the function of bacterial condensins in promoting DNA segregation to daughter cells is not generally conserved. A recent bioinformatics study predicted a role for MksBEFG complexes (termed Wadjet system) in plasmid-related defense, where heterologous complex expression conveyed protection against the uptake of a high-copy number plasmid^62^. However, function of MksBEFG in its native host had not been addressed before. We could show that the Mks system is indeed involved in the control of plasmid copy numbers, and that there is no involvement of this system in chromosome organization. As low-copy number vectors used in this study harbor replicons of a related *Corynebacterium* genus, the impact of MksBEFG on plasmids may be based on adaptations of plasmid-specific characteristics to the host species, such as the structural organization of the replication origin or replication-associated proteins^63–65^. Our findings share a fascinating similarity to specific eukaryotic condensing homologs such as Rad50, being the closest eukaryotic relative to MukB/MksB^23,40^. It was recently shown that Rad50–CARD9 complexes sense foreign cytoplasmic DNA in mammalian cells acting in innate immune responses against viral DNA^66^. In addition, the more distantly related eukaryotic SMC5/6 complex had been shown to act in a defense mechanism against circular hepatitis B virus DNA, resembling the specific effect of prokaryotic MksBEFG on plasmids^67^. Together, our data lend support to the notion that condensins’ function in innate immunity is an ancient mechanism. However, notably, we provide evidence that the MksBEFG complex is the only known condensin amongst pro- and eukaryotes to date that exclusively impacts on non-chromosomal DNA. For MksBEF systems, it has been proposed that a fourth subunit, MksG, is important for function in plasmid maintenance^62^. We could verify that MksG is part of the MksBEF complex of *C. glutamicum*. This assumption is in line with divergent functions observed between *C. glutamicum* MksBEFG and the structurally related *P. aeruginosa* MksBEF complex that is assumed to act in chromosome organization due to a synthetic DNA-segregation phenotype in combination with SMC/ScpAB^40^. The direct interaction of a Mks complex with a polar scaffold protein such as the *C. glutamicum* DivIVA has not been described before. A challenging question for the future will be to determine the detailed mechanism of MksBEFG in plasmid defense and the putative role of the DivIVA–Mks interaction in this process.

We further describe here that SMC/ScpAB is indeed the major factor of replichore cohesion and chromosome organization in *C. glutamicum*. Like in *B. subtilis*, SMC is preferentially loaded onto the chromosome by a ParB/*parS* loading complex before spreading to the entire chromosome. The mild DNA-partitioning defects of a *smc* deletion in combination with a ParB-eYFP modification (Supplementary Table 1) strongly suggest a supportive role of SMC/ScpAB in the process of nucleoid separation, yet the *smc* phenotype appears to be entirely compensated by ParB. Therefore, our data demonstrate that the conserved role for SMC in chromosome organization^10,20–22,35,36^ is also maintained in *C. glutamicum*. Moreover, bacterial two-hybrid analyses of SMC/ScpAB subunits evidence a self-interaction of *C. glutamicum* kleisin ScpA (Fig. 3), which has not been described in other organisms before. Based on this result, we speculate that *C. glutamicum* SMC/ScpAB might form dimers via kleisin subunits similar to *E. coli* MukBEF complex^68,69^. These data point to a handcuffing model, where two SMC/ScpAB complexes are physically coupled together and translocate in pairs along the chromosome, similar as suggested for *B. subtilis*^31^. We further describe a new phenotype for a ParB^R175A^ point mutation in *C. glutamicum* that decreases SMC recruitment or blocks SMC release from its loading site. Building on this, we observe a weak interaction signal of ParB^R175A^ with ScpB in bacterial two-hybrid analyses. Alternatively, SMC/ScpAB remains indirectly entrapped in higher-order ParB^R175A^ nucleocomplexes, which possess altered DNA-folding properties. In either case, this mutant underlines the crosstalk between SMC/ScpAB and ParB nucleoprotein complexes in bacterial nucleoid organization.

Analysis of ParB complexes using two-dimensional (2D) PALM reveals ParB-dense regions within clusters that correlate to the number of ParB-enrichment zones along adjacent *parS* sites. In line with a current study on ParB cluster-assembly in *V. cholerae*^70^, we suggest that these subclusters derive from independent nucleation and caging events, which merge into one ParB-macrocomplex per *oriC* in *C. glutamicum*. Presence of a single *parS* site leads to formation of almost globular ParB densities. Using Hi-C approaches, we further show that *parS* sites and ParB are major factors of chromosome folding in *C. glutamicum* as previously shown in other organisms^10,21,22,36^. *C. glutamicum* chromosome adopts a global folding with a strong cohesion between the two chromosomal arms as expected from a bacterium harboring a longitudinal chromosomal organization similar to *B. subtilis* and, to a lesser extent, *C. crescentus* (Fig. 1). Our analysis also suggests the existence of a chromosomal domain at *parS* sites in *C. glutamicum* as previously observed in *B. subtilis*, but with important differences: *parS* sites in *C. glutamicum* are only found on one side of the *oriC* locus and appeared to be at the edge of the nucleoid structure as observed in *C. crescentus*. A hairpin structure as it was observed in *B. subtilis* is absent in *C. glutamicum*^10^. Contact maps of a strain with an ectopic *parS* site feature a bow-shaped structure reflecting an asymmetry in arm interaction, which has been shown before in *B. subtilis* and *C. crescentus*^21,22^. Zipping of the chromosome is not complete and the ectopic *parS* site does not reorient the entire chromosome. Therefore, additional factors are involved in chromosome localization that supplement polar ParB-*parS* binding to DivIVA.

Importantly, we describe ParB/*parS*-dependent DNA contacts of the *parS* region with the entire nucleoid, indicating that *oriC* segregation occurs across the entire nucleoid. This is in accord with the *ori-ter* configuration of the nucleoid in *C. glutamicum*. Different from *C. glutamicum*, *B. subtilis* SMC is required for segregation signals that do not spread along the whole chromosomal length^21^. Based on our data, we propose the following model shown in Supplementary Fig. 14: organisms with polarly localized *oriC*s and a longitudinal chromosome organization rely on ParAB for *oriC* segregation, as they can use the DNA scaffold as a track. By contrast, species with a central replication factory cannot efficiently use ParAB. *B. subtilis* is an exception, since here a longitudinal chromosome orientation is present during sporulation and, hence, *parAB* (*spo0J*/*soj*) phenotypes are only obvious during spore formation. Segregation of otherwise transversally arranged *B. subtilis* chromosomes during vegetative growth rely on an initial SMC-driven *oriC* segregation along a limited fraction of the nucleoid instead^21^. Consequently, SMC/ScpAB-mediated replichore cohesion is likely dispensable for *oriC* segregation in bacteria with a strict longitudinal chromosome arrangement that allows for efficient ParABS-driven chromosome partitioning.

## Methods

### Bacterial strains, plasmids, and oligonucleotides

Primers, plasmids, and strains used in this study are listed in Supplementary Data 1 and 2.

For protein–protein interaction screens, genes of interest were amplified via PCR, digested with respective enzymes, and ligated into bacterial two-hybrid vectors^71^. *E. coli* DH5α were utilized for plasmid cloning. Genes *divIVA* and *parB*/*parB*^R175A^ were amplified using primer pairs DivIVA-XbaI-F/DivIVA-BamHI-R and ParB-XbaI-F/ParB-BamHI-R from genomic DNA or pK19mobsacB-ParBR175A, and resulting fragments were digested with XbaI/BamHI. For amplification of *scpA*, *scpB*, *mksE*, *mksF*, and *mksG*, primer pairs ScpA-XbaI-F/ScpA-XmaI-R, ScpB-XbaI-F/ScpB-XmaI-R, MksE-XbaI-F/MksE-XmaI-R, MksF-XbaI-F/MksF-XmaI-R, and MksG-XbaI-F/MksG-XmaI-R were utilized, followed by restriction digests with XmaI/XbaI. Primer pairs SMC-XbaI-F/SMC-KpnI-R and MksB-XmaI-F/MksB-KpnI-R were used for PCR amplification of genes *smc* and *mksB*, which were subsequently digested with XbaI/KpnI or XmaI/KpnI. To increase the distance of XmaI and KpnI restriction sites, a short sequence was inserted in between these sites by overhang PCRs using pUT18C-mcs-HindIII-F, pUT18-mcs-PvuII-F, pKNT25-mcs-NheI-F, or pKT25-mcs-HindIII-F in combination with pUT18(C)/pK(N)T25-mcs-KpnI-R for plasmids pUT18C, pUT18, pKT25, and pKNT25, respectively. Resulting fragments and corresponding vectors were digested with HindIII/KpnI, PvuII/KpnI, or NheI/KpnI and subsequently ligated, resulting in plasmids pUT18_mcs, pUT18C_mcs, pKNT25_mcs, and pKT25_mcs. All digested gene fragments mentioned above were ligated into pUT18, pUT18C, pKNT25, and pKT25 or pUT18_mcs, pUT18C_mcs, pKNT25_mcs, and pKT25_mcs, respectively.

Derivatives of the suicide integration vector pK19mobsacB were used for clean allelic replacements in *C. glutamicum*, containing the modified genomic region of interest including its 500 bp up- and downstream homologous flanking sequences. Plasmid cloning was performed using *E. coli* DH5α.

To construct pK19mobsacB-Δsmc 500 bp upstream and downstream of *smc* were PCR amplified using primer pairs Δsmc-BamHI-up-F/Δsmc-up-R and Δsmc-D-F/ Δsmc-EcoRI-D-R, respectively. Both fragments served as templates in an overhang PCR, yielding a 1000 bp fragment, which was digested with BamHI and EcoRI and subsequently ligated into pK19mobsacB. pK19mobsacB-ΔSMCload was constructed accordingly, using primer pairs ΔSMCload-HindIII-up-F/ΔSMCload-up-R and ΔSMCload-D-F/ΔSMCload-SalI-D-R, and HindIII in combination with SalI for restriction digest. For construction of pK19mobsacB-ΔmksB up-/and downstream regions of *mksB* were PCR amplified using primers ΔmksB-HindIII-up-F/ΔmksB-PstI-up-R and ΔmksB-PstI-D-F/ΔmksB-XbaI-D-R. Resulting 500 bp fragments were digested with HindII/ PstI and PstI/ XbaI and consecutively ligated into pK19mobsacB.

Fluorescent C-terminal fusions of ParB protein with PAmCherry or mNeongreen were obtained by utilizing plasmids pK19mobsacB-parB-mNeonGreen and pK19mobsacB-parB-PAmCherry. To this end, the *eYFP* sequence of plasmid pK19mobsacB-parB-eYFP^41^ was replaced by respective fluorophore sequences, which were amplified via PCR using PAmCherry-SalI-F/PAmCherry-XbaI-R primers and digested with SalI and XbaI.

For fluorescent versions of SMC and MksB proteins plasmids pK19mobsacB-smc-mCherry, pK19mobsacB-mksB-mCherry and pK19mobsacB-mksB-PAmCherry were constructed. At first, 500 bp regions up- and downstream of the 3′-end of *smc* or *mksB* were amplified using primer pairs SMC-HindIII-up-F/SMC-SphI-up-R and SMC-BamHI-D-F/SMC-EcoRI-D-R or MksB-HindIII-up-F/MksB-Sph-up-R and MksB-BamHI-D-F/MksB-EcoRI-D-R. Fluorophore sequences were amplified with primers PAmCherry-SalI-F/mCherry-BamHI-R for SMC-mCherry and MksB-PAmCherry fusion or with primers PAmCherry-SalI-F/mCherry-XbaI-R for the MksB-mCherry fusion construct. Up- and downstream fragments were digested via HindIII/SphI and BamHI/EcoRI, whereas enzymes SalI/BamHI or SalI/XbaI were utilized for restriction digest of fluorophore sequences fused to *smc* or *mksB*, respectively. Fragments were subsequently ligated into the pK19mobsacB plasmid, starting with the corresponding downstream region, followed by the fluorophore sequence and finally the upstream region.

To place part of a putative SMC binding site upstream of the *parS* cluster into an intergenic region 3′ of cg0177 (Supplementary Fig. 10), genomic sequences 500 bp up- and downstream of the insertion site were amplified using primer pairs cg0177-HindIII-up-F/cg0177-SalI-up-R and cg0177-XmaI-D-F/cg0177-EcoRI-D-R; part of the genomic SMC binding site (1.1 Kb) was amplified using primers SMCload-SalI-F and SMCload-XmaI-R. Resulting fragments were digested with HindII/SalI, SalI/XmaI, and XmaI/EcoRI, and consecutively ligated into pK19mobsacB, obtaining the plasmid pK19mobsacB-SMCload-cg0177.

Plasmid pK19mobsacB-SMCload-r was constructed for the partial replacement of the SMC binding site (1.1 Kb) with a *B. subtilis* genomic region of identical size. For amplification of up- and downstream 500 bp regions, primer pairs ΔSMCload-HindIII-up-F/SMCload-SphI-up-R and SMCload-PstI-D-F/ΔSMCload-SalI-D-R were utilized, whereas the replacement sequence was amplified from *B. subtilis* genomic DNA via SMCloadr-SphI-F/SMCloadr-PstI-R. After digestion with enzymes HindIII/SphI, PstI/SalI, or SphI/PstI, fragments were successively ligated into pK19mobsacB.

Further, all *parS* sites were mutated comprising new XmaI or SalI restriction sites (see Supplementary Fig. 2). For mutation of *parS1* primer pairs, parS1mut-HindIII-up-F/parS1mut-XmaI-up-R and parS1mut-XmaI-D-F/parS1mut-EcoRI-D-R were utilized to mutate *parS1* and to amplify sequences 500 bp up- and downstream of *parS1*. Restriction digest was performed with both fragments using HindIII/XmaI or XmaI/EcoRI, respectively. Subsequent ligation into pK19mobsacB yielded plasmid pK19mobsacB-parS1mut. To mutate *parS2*, *parS3*, *parS4*, *parS7*, and *parS8*, plasmid construction was performed in the same way using primers parS2mut-HindIII-up-F/parS2mut-XmaI-up-R and parS2mut-XmaI-D-F/parS2mut-EcoRI-D-R, parS3mut-HindIII-up-F/parS3mut-XmaI-up-R and parS3mut-XmaI-D-F/parS3mut-EcoRI-D-R, parS4mut-HindIII-up-F/parS4mut-XmaI-up-R and parS4mut-XmaI-D-F/parS4mut-EcoRI-D-R, parS7mut-HindIII-up-F/parS7mut-XmaI-up-R and parS7mut-XmaI-D-F/parS7mut-EcoRI-D-R, or parS8mut-HindIII-up-F/parS8mut-XmaI-up-R and parS8mut-XmaI-D-F/parS8mut-EcoRI-D-R for amplification of fragments up- and downstream of the respective *parS* site. Matching fragments were each digested and ligated into pK19mobsacB, as exemplified for pK19mobsacB-parS1mut construction, resulting in plasmids pK19mobsacB-parS2mut, pK19mobsacB-parS3mut, pK19mobsacB-parS4mut, pK19mobsacB-parS7mut, and pK19mobsacB-parS8mut.

As *parS5* and *parS6*, as well as *parS9* and *parS10*, are localized in close proximity on the genome (<100 bp distance), their deletions were accomplished using in each case one plasmid for both *parS* sites. For construction of pK19mobsacB-parS5_6mut genomic region upstream of *parS5*, downstream of *parS6*, and in between, both sides were PCR amplified using parS5mut-HindIII-up-F/parS5mut-SalI-up-R, parS6mut-XmaI-D-F/parS6mut-EcoRI-D-R, and parS5mut-SalI-D-F/parS6mut-XmaI-up-R, and fragments were digested with HindIII/SalI, XmaI/EcoRI, or SalI/XmaI, respectively, and ligated into pK19mobsacB. Construction of pK19mobsacB-parS9_10mut was performed accordingly, using primer pairs parS9mut-HindIII-up-F/parS9mut-SalI-up-R, parS10mut-XmaI-D-F/parS10mut-EcoRI-D-R, and parS9mut-SalI-D-F/parS10mut-XmaI-up-R for fragment amplification.

Insertion of *parS* 3′ of *cg0108*, *cg0904*, and *cg2563* (9.5°, 90°, and 270° chromosomal positions) were achieved via plasmids pK19mobsacB-parS-cg0108, pK19mobsacB-parS-cg0904, and pK19mobsacB-parS-cg02563. Primers containing *parS* sites were used to amplify regions 500 bp up- and downstream of the corresponding *parS* insertion site, namely parS-cg0108-SalI-up-F/parS-cg0108-up-R and parS-cg0108-D-F/parS-cg0108-XmaI-D-R, parS-cg0904-HindIII-up-F/parS-cg0904-up-R and parS-cg0904-D-F/parS-cg0904-NheI-D-R, or parS-cg2563-HindIII-up-F/parS-cg2563-up-R and parS-cg2563-D-F/parS-cg2563-NheI-D-R, respectively. Each fragment pair served as template in an overhang PCR, yielding 1000 bp sequences with central *parS* sites. After restriction digest with SalI/XmaI or HindIII/NheI, each fragment was ligated into pK19mobsacB. Plasmid pK19mobsacB-parS-Δint for *parS* insertion at *terC* was constructed in the same way, however by replacing an entire gene (*cg1752*, 180° chromosomal position). Regions 500 bp N- and C-terminally of *cg1752* were amplified using parS-Δint-HindIII-up-F/parS-Δint-up-R and parS-Δint-D-F/parS-Δint-NheI-D-R.

For construction of pK19mobsacB-parBR175A, primer pairs ParB-N-ter-HindIII-F/ParB-R175A-R and ParB-R175A-F/ParB-C-ter-SalI-R were used to amplify the N- and C-terminal parts of *parB* surrounding the coding region of ParB^R175^. Primers introduce point mutations into this codon and into a neighboring SacI restriction site, resulting in fragments of 528 bp and 625 bp length. Overhang PCR yielded a full *parB* sequence that was cut with HindIII/SalI and ligated into pK19mobsacB. pK19mobsacB-smcE1084Q was obtained in an analogous manner. Amplification of 500 bp genomic regions surrounding codon SMC^E1084^ were performed using primer pairs E1084Q-HindIII-up-F/E1084Q-up-R and E1084Q-D-F/E1084Q-BamHI-D-R, which further yield in an E1084Q mutation and an additional XbaI restriction site 3′ of the codon sequence.

His-tagged versions of ParB and ParB^R175A^ were generated by applying PCR (ParB-NdeI-F/ParB-XhoI-R) following a restriction digest (NdeI/XhoI) of the respective DNA fragment and ligation into pET-16b expression vector yielding pET-16b-ParB and pET-16b-ParBR175A.

For construction of the *E. coli*–*C. glutamicum* shuttle expression vector pEKEx2-mCherry the mCherry sequence was amplified via PCR using mCherry-SacI-F/ mCherry-EcoRI-R, digested with corresponding restriction enzymes, and ligated into the empty pEKEx2.

Vectors were transformed via electroporation into *C. glutamicum* cells^72^. Genomic integration of pK19mobsacB plasmids were selected on kanamycin, whereas the second crossover event was confirmed by growth on 10% sucrose. Screening of allelic replacements in *C. glutamicum* Δ*smc*, Δ*mksB*, and Δ*parB* was performed by colony PCR using primer pairs Δsmc-seq-700up-F/Δsmc-seq-700D-R, ΔmksB-seq-700up-F/ΔmksB-seq-700D-R, and ParB-seq-800up-F/ParB-seq-800D-R. Fluorescent fusions of ParB, SMC, and MksB were confirmed via primer pairs ParB-N-ter-SalI-F/ParB-seq-800D-R, SMC-seq-1589bp-F/Δsmc-seq-700D-R, and MksB-seq-1595bp-F/ΔmksB-seq-700D-R, respectively. Insertions of the partial *smc loading site* in an intergenic region 3′ of *cg0177* were screened using primer pairs cg0177-seq-700up-F/cg0177-seq-700D-R. To identify genomic *parS* mutations, respective regions were amplified using upstream-forward and downstream-reverse primers as used for plasmid construction and digested with either XmaI or SalI. Sequencing of *parS* loci was performed for further verification. For verification of *parS* insertions 3′ of *cg0108*, *cg0904*, and *cg2563* or for replacement of *cg1752* by *parS* genomic loci were amplified with primers cg0108-seq-400up-F/cg0108-seq-200D-R, cg0904-seq-100up-F/cg0904-seq-100D-R, cg2563-seq-200up-F/cg2563-seq-300D-R, or Δint-seq-700up-F/Δint-seq-700D-R, respectively, followed by a control restriction digest using PmlI. Screening for *parB*^*R175A*^ was performed by amplification of *parB* including 800 bp up- and downstream regions via primers ParB-seq-800up-F/ParB-seq-800D-R. A control digest was conducted with the resulting fragment using SacI. Integration of the point mutation smc^E1084Q^ was verified by amplification of the respective genomic region (E1084Q-HindIII-up-F/mCherry-EcoRI-R), followed by restriction digests using XbaI.

Assembly strategies of multiple consecutive allelic replacements are explained hereafter. *C. glutamicum* strains CBK002, CBK004, and CBK010 were obtained via transformation of pK19mobsacB-ΔparB, pK19mobsacB-ΔmksB, or pK19mobsacB-parB-eYFP into strain CDC026 lacking *smc* and strain CBK003 (Δ*mksB* Δ*parB*) was constructed using the genetic background of CBK001 (Δ*mksB*). Further, CBK004 served as parent strain for construction of CBK005 and CBK011 harboring additional mutations Δ*parB* and *parB∷parB-eYFP*, respectively. The dual-reporter strain CBK013, expressing ParB-mNeonGreen in combination with SMC-mCherry, was constructed via transformations of pK19mobsacB-smc-mCherry into CBK008; strain CBK014 derives from CBK012 transformed with pK19mobsacB-ΔparB. The complete loss of *parS* sites in strain CBK024 was accomplished via successive allelic replacements of *parS* by mutated sequences: the mutation of *parS2* (CBK017) followed the mutation of *parS3* (CBK016); thereupon, *parS4* (CBK018) was mutated followed by *parS5* and *parS6* (CBK019). Next, *parS7* (CBK020) mutation, *parS8* mutation (CBK021), *parS9* mutation (CBK022), *parS10* mutation (CBK023), and *parS1* mutation (CBK024) were accomplished consecutively. CBK090 is a derivative of CBK022. CBK025, CBK027, and CBK029 derive from strain CBK023, which was transformed with pK19mobsacB plasmids coding for *parB-eYFP*, *parB-mCherry2*, and *parB-PAmCherry*, respectively. Accordingly, stains CBK026, CBK28, CBK032, and CBK087 are CBK024 derivatives harboring either endogenous *parB-eYFP*, *parB-mCherry2*, *smc-mCherry*, or *parB-PAmCherry*, whereas strains CBK030 and CBK031 obtained from CBK022 via transformation of pK19mobsacB-parB-mCherry2 or pK19mobsacB-parB-PAmCherry. CBK091 was obtained by transformation of CBK090 with pK19mobsacB-parB-mCherry2. CBK033 and CBK035 were generated by transformation of strain CBK012 expressing SMC-mCherry with plasmids pK19mobsacB-ΔSMCload or pK19mobsacB-SMCload-r; a further transformation of CBK033 with pK19mobsacB-SMCload-cg0177 yielded CBK034. To introduce *parS* sites at different regions within the *C. glutamicum* genome CBK024 served as parental strain: *parS*-insertions at chromosomal 9.5°, 90°, 270°, and 180° positions were achieved via transformation of either pK19mobsacB-parS-cg0108 (CBK036), pK19mobsacB-parS-cg0904 (CBK037), pK19mobsacB-parS-cg2563 (CBK038), or pK19mobsacB-parS-Δint (CBK039). Additional allelic replacements of *parB* or *smc* with fluorophore-coupled versions *parB-eYFP* or *parB-mCherry2* and *smc-mCherry* in the above-named strains resulted in CBK040-CBK045. Second, *parS*-insertion in CBK037 was combined with a *smc* deletion by transformation of pK19mobsacB-Δsmc yielding CBK046. Lastly, strains CBK047–CBK051, which express mutant ParB^R175A^ or SMC^E1084Q^ proteins derive from CBK006, CBK027, and CBK012 transformed with plasmid pK19mobsacB-parBR175A or pK19mobsacB-smcE1084Q, respectively. GGCB1C8 was used for subsequent construction of strains CBK092 and CBK093 by transformation with either pK19mobsacB-mksB-mCherry or pK19mobsacB-mksB-PAmCherry.

### Plasmid extraction from *C. glutamicum* cells

*C. glutamicum* cells were grown in 10 ml BHI medium to exponential growth phases in presence of selection antibiotic, following incubation with 20 mg/ml lysozyme in P1 buffer (NucleoSpin® Plasmid Kit, Macherey-Nagel) overnight at 30 °C. Subsequently, plasmids were extracted by using the plasmid kit according to manufacturer’s instruction.

### Growth conditions and media

*E. coli* cells were grown at 37 °C in Lysogeny Broth (LB) medium supplemented with 50 µg/ml kanamycin when appropriate. Growth experiments of *C. glutamicum* cells were performed using brain heart infusion medium (BHI, Oxoid^TM^) or CGXII medium^73^ supplemented with 4% glucose or 120 mM acetate at 30 °C. Cells were always preinoculated in BHI overnight; for growth in minimal media cells were first inoculated in BHI and rediluted in the corresponding growth media overnight for pre-cultivation. Finally, cell cultures were adjusted to an OD_600_ of 0.5 for BHI and to an OD_600_ of 1 for growth in CGXII medium. Kanamycin (25 μg/ml) was added where applicable.

### Protein identification via immunoprecipitation and mass spectrometry

Immunoprecipitation of SMC and MksB interaction partners was performed with strains CBK012 and CBK015, further including strain CBK052 as negative control. Lysate of exponentially grown cells was used for immunoprecipitation via magnetic RFP-Trap® agarose beads. For proteomic analysis samples were further processed and analyzed by liquid chromatography tandem mass spectrometry (LC-MS/MS) to identify and quantify proteins in all samples.

For immunoprecipitation of interacting proteins, strains CBK012, CBK015, and CBK052 were cultivated in BHI medium using culture flasks pretreated with 0.5% sodium hypochlorite. CBK052 was induced at OD600 ~1 with 0.5 mM isopropyl β-d-1-thiogalactopyranoside (IPTG). Exponentially growing cells (OD600 = 3, 10 ml) were collected, washed once in 10 ml washing buffer (Tris-HCl pH 7.5 10 mM; NaCl 150 mM; EDTA 0.5 mM) and resuspended in 1.5 ml washing buffer supplemented with 1 mM phenylmethylsulfonyl fluoride in EtOH. All following steps were performed at 4 °C. After cell disruption via FastPrep®-24 (MP Biomedicals) at 10 × 6.5 m/s, 30 s cell debris was removed by centrifugation at 18,000 × *g*. Immunoprecipitation was performed with 25 μl magnetic RFP-Trap® agarose beads (Chromotek) incubated in 1 ml Lysate for 1 h. Thereupon, beads were washed three times in washing buffer and again washed three times in 100 mM ammonium bicarbonate prior to storage at −20 °C.

For proteomic analysis of interacting proteins, the magnetic beads were first washed with 50 µl of 100 mM TRIS pH 7.6. Subsequently, 50 µl of 100 mM TRIS pH 7.6 containing 4 M urea, 5 mM dithiothreitol for reduction of disulfide bond, and 0.2 µg of LysC for predigestion of proteins were added to each sample. After incubation of 3 h, 100 µl of 100 mM TRIS pH 7.6 and 10 mM iodoacetamide were added for blocking of free cysteine side chains and samples were incubated in the dark for 5 min. Samples were diluted with 100 µl TRIS pH 7.6 to reduce the urea concentration and 1 µg of trypsin was added to each sample. The samples were incubated for 14 h to complete protein digestion and subsequently trifluoroacetic acid was added to a final concentration of 0.5% to acidify the samples. Peptide mixture were separated from the magnetic beads before the desalting step. The beads were washed 2× with 75 µl of 0.1% formic acid (FA) and the wash solvent was combined with the peptide mixtures. For sample desalting, three discs were stamped from C18 discs (Empore C18, 3 M) and placed into a 200 µl pipette tip. Following binding of peptides, stage tips were washed 2× with 60 µl of 0.1% FA and peptides were eluted with 40% acetonitrile containing 30% methanol and 0.1% FA. Samples were dried in a speedvac and resuspended in 10 µl of 0.1% FA. Peptide mixtures were analyzed by LC-MS/MS to identify and quantify proteins in all samples. First, peptides were separated by nano-reversed phase chromatography using a linear gradient from 2 to 35% acetonitrile over 50 min in 0.1% FA on an in-house-packed chromatography column in a nano-electrospray emitter tip. Eluting peptides were directly infused into the mass spectrometer (QExactive, Thermo Fisher) and detected in positive ionization mode. The operating cycle was programmed to detect peptides in the range from 300 to 1600 m/z and up to 10 precursors were selected for MSMS analysis by CID fragmentation. Precursor ions required a charge state between +2 and +6 and a minimal signal intensity of 6 × 10e4.

Protein mapping and quantitative analysis raw LC-MS/MS data were searched against a C. glutamicum database retrieved from Uniprot (vs. 03/2017, 3093 protein entries) using a forward/reversed search by the Andromeda algorithm within the MaxQuant software suite. Peptides hits were searched with 17 p.p.m. precursor mass deviation in the first search and 3 p.p.m. for the main search. For MS/MS spectra, a mass accuracy of 25 p.p.m. was set. As variable modifications, acetylation of the protein N-terminus, STY-phosphorylation, and methionine oxidation were selected. Carbamidomethylation of cysteine was the only fixed modification. Peptide match results were sorted by their probability score and filtered for 2% reversed peptide hits and 5% reversed protein hits.

To calculate protein enrichments and significance values, reversed protein hits and proteins with less than three quantitative values in any of the three sample types (control, mksB IP, and smc IP) were filtered out. The iBAQ-values were log2 transformed and median normalized. In case of one missing value in the triplicate measurements, the value was imputed using a closest neighbor method; for more missing data points, a random value from a standard distribution downshifted by a factor of 1.8 from the sample distribution and width of 0.3 was selected. Samples were compared using a Student’s *t*-test, which was false discovery rate controlled by sample permutation.

### Bacterial two-hybrid screening

Protein interactions obtained by mass spectrometry were confirmed via bacterial two-hybrid assays^71^, using compatible vectors expressing adenylate cyclase subunits T25 and T18 (pKT25/ pKNT25 and pUT18/ pUT18C). *E. coli* BTH101 co-transformed with respective vectors were plated on indicator medium LB/X-Gal (5-bromo-4-chloro-3-indolyl-β-D-galactopyranoside, 40 μg/ml) supplemented with IPTG (0.5 mM) and antibiotics kanamycin (50 μg/ml), carbenicillin (100 μg/ml), and streptomycin (100 μg/ml), and incubated at 30 °C for 24 h. Interacting hybrid proteins were identified by blue–white screening and β-galactosidase assays in a 96-well plate format as previously described^74^. In brief, 0.1 ml of overnight cultures of co-transformants were transferred to 96-well plates. Cells were pelleted and re-suspend in 80 µl Z-buffer (60 mM Na_2_HPO_4_, 40 mM NaH_2_PO_4_, 10 mM KCl, 1 mM MgSO_4_) including 50 mM β-Mercaptoethanol and 10 µl of chloroform and SDS (0.1%) were added per well prior to gentle mixing. After centrifugation at 1000 × *g* for 10 min, clear lysates were transferred to a clean 96-well plate and 20 µl of 2-Nitrophenyl β-d-galactopyranoside (4 mg/ml in in Z-buffer) were added to each well at 30 °C. The reaction time of β-galactosidase activity was recorded until the addition of 30 µl of Na_2_CO_3_ (1 M). Absorbance was determined at OD420 using a Tecan plate reader. Co-transformants harboring empty plasmids or pUT18C-zip/ pKT25-zip plasmids served as positive and negative controls. Miller units of negative controls served as reference and were set to zero. Miller units of any other sample were normalized accordingly. All C- and N-terminal combinations of hybrid proteins were assayed and positive signals were confirmed through at least three replicates.

### Fluorescence microscopy

Fluorescence microscopy was performed with exponentially grown cells mounted on agarose coated slides (1% agarose). Images were acquired on an Axio-Imager M1 fluorescence microscope (Carl Zeiss) with an EC Plan Neofluar ×100/1.3 oil Ph3 objective and a 2.5x optovar. Fluorescence of protein fusions with eYFP (enhanced yellow fluorescent protein) and mCherry/mCherry2 or DNA stained via Hoechst 33342 (1 µg/ml, Thermo Scientific) were detected using filter sets 46 HE YFP (EX BP 500/25, BS FT 515, and EM BP 535/30), 43 HE Cy 3 shift free (EX BP 550/25, BS FT 570, and EM BP 605/70), and 49 DAPI shift free (EX G 365, BS FT 395, and EM BP 445/50). Live-cell imaging and detection of fluorescently labeled condensin subunits were carried out using a Delta Vision Elite microscope (GE Healthcare, Applied Precision) with a standard four color InsightSSI module, a ×100/1.4 oil PSF U-Plan S-Apo objective and the YFP (EX BP 513/17 and EM BP 548/22) and mCherry (EX BP 575/25 and EM BP 625/45) specific filter sets. To conduct time-lapse experiments, exponentially grown cells were diluted to an OD_600_ of 0.01 in BHI and loaded in a microfluidic chamber (B04A CellASIC®, Onix); the environmental chamber was heated to 30 °C and 0.75 psi were applied for nutrient supply throughout the experiment. Images were taken in 5 min intervals. For display of cellular fluorescence profiles sorted by cell length, FIJI and R software were utilized^50,75^.^75,76^.

### ChIP combined with sequencing

Briefly, cells were crosslinked (1% formaldehyde) for 30 min at room temperature (RT) and lysed. DNA was sheared by sonication, incubated with α-mCherry antibody for 2 h at 4 °C, washed subsequently, and crosslinks were reverted at 65 °C. DNA purification was followed by library preparation and sequencing using an Illumina MiSeq system. Reads were aligned to the *C. glutamicum* ATCC 13032 genome sequence (GeneBankID: BX927147.1). Further data analysis was performed using online tools^77^. More in details, in vivo ChIP experiments with *C. glutamicum* ParB, SMC, or MksB proteins were conducted using strains with allelic replacements of respective proteins with mCherry-tagged versions. Exponentially growing cells were crosslinked in 1% formaldehyde for 30 min at RT; for SMC- and MksB-mCherry ChIP experiments, cells were treated with Crosslink Gold (Diagenode) for 30 min at RT and washed twice in phosphate-buffered saline (PBS; 137 mM NaCl, 10 mM Na_2_HPO_4_, 1.8 mM KH_2_PO_4_, 2.7 mM KCl pH 7.4.) prior to formaldehyde crosslinking. Fixed cells were subsequently washed in PBS and suspended in protoplast buffer (50 mM Tris pH 7.4, 50 mM NaCl, 10 mM EDTA, 0.5 M sucrose, EDTA-free protease inhibitor cocktail) supplemented with 20 mg/ml of lysozyme for 2 h at 37 °C. After washing in protoplast buffer, pellets were resuspended in buffer L (50 mM HEPES-KOH pH 7.55, 40 mM NaCl, 1 mM EDTA, 1% Triton X-100, 0.1% deoxycholate, 0.1 mg/ml RNaseA; EDTA-free protease inhibitor cocktail) and DNA was sheared into fragments of around 800 bp length by sonication using an ultrasonic cell disruptor (Branson Ultrasonics Sonifier™; 20% amplitude, pulse 0.5 s on/off, 6 × 20 s). Following removal of cell debris (20,000 × *g*, 10 min, 4 °C). Aliquots of cell extracts were stored for later use. Dynabeads™ Protein G (Thermo Fisher Scientific) were bound to an α-mCherry Fantibody (BioVision, Inc.) in buffer L for 1.5 h at 4 °C, washed in buffer L, and subsequently incubated with cell extract for 2 h at 4 °C. Thereafter, beads were washed in buffer L, in buffer L5 (50 mM HEPES-KOH pH 7.55, 500 mM NaCl, 1 mM EDTA, 1% Triton X-100, 0.1% deoxycholate), in buffer W (10 mM Tris-HCl pH 8, 250 mM LiCl, 0.5% NP-40, 0.5% deoxycholate, 1 mM EDTA), and TE buffer (10 mM Tris-HCl pH 8, 1 mM EDTA) consecutively and finally resuspended in TES buffer (10 mM Tris-HCl pH 8, 10 mM EDTA, 1% SDS). Extract samples were also supplemented with TES buffer and SDS to a final concentration of 1% SDS; crosslinks were reverted at 65 °C overnight. Phenol–chloroform extraction yielded DNA pellets, which were further purified using a DNA purification kit (QIAquick®, Qiagen). qPCR was applied to confirm protein enrichment at specific chromosomal loci. Immunoprecipitation and extract samples were diluted 1:10 and 1:100 in water, yielding concentrations of ~0.2–0.4 ng/μl.

For sequencing analyses, libraries of ChIP samples were prepared followed by sequencing utilizing an Illumina MiSeq system. Reads were aligned to the C*. glutamicum* ATCC 13032 genome sequence (GeneBankID: BX927147.1), where RES167-specific genome deletions were manually cut using CLC Genomics Workbench. Data of extract and corresponding ChIP sample were each normalized based on read counts and the ratio of the number of reads per 0.5 Kb bin were determined via the Galaxy web platform^77,78^.

### Real-time PCR

DNA amplification was performed using a 2× qPCR Mastermix (KAPA SYBR®FAST, Peqlab) according to the manufacturer’s instruction, where reaction volumes of 10 µl contained 200 nM oligonucleotides and 4 μl of diluted DNA, respectively. Samples were measured in technical duplicates via an iQ5 multicolor real-time PCR detection system (Bio-Rad) and CT-values were determined via the Bio Rad-IQ™5 software version 2.1. Primer efficiencies were estimated by calibration dilution curves and slope calculation^79^; data were analyzed via the 2^-∆CT^ method^80^ accounting for dilution factors and sample volumes used for DNA purification. qPCR data of ChIP samples were normalized according to the ParB-mCherry2 signal obtained at locus *parS1* in the wild-type background, serving as reference in each experiment.

### Protein purification

ParB protein production was performed in *E. coli* BL21 pLysS via the pET-16b vector-based system. Cells were grown in LB at 37 °C; gene expression was induced adding 1 mM IPTG following growth for 12 h at 18 °C. Subsequently, cells were suspended in washing buffer (50 mM Tris-HCl pH 7.4; 100 mM NaCl; 5 mM; MgCl_2_; 1 mM dithiothreitol) containing EDTA-free proteinase inhibitor (cOmplete^TM^, Sigma) and DNAseI, and lysed using a high-pressure cell homogenizer. Cell debris and membranes were removed by centrifugation at 4 °C, 1700 g for 20 min, and 150,000 × *g* for 45 min, respectively. Thereupon, batch purifications of His-tagged protein were performed under native conditions using Ni-NTA agarose (Protino®, Macherey-Nagel) according to manufacturer’s instruction. In brief, the equilibrated gel was incubated with clarified lysate for 60 min at 4 °C under gentle agitation and washed twice in washing buffer containing 80 mM imidazole. Proteins were eluted in three steps using washing buffer with an imidazole concentration of 300 mM, concentrated via Amicon filter units (Merck) and further purified by applying size exclusion chromatography using an ÄKTApurifier system with a Superdex^TM^ 200 gel filtration column (GE Healthcare Life Sciences).

### Electrophoretic mobility shift assay

DNA-ParB binding was assayed using purified protein and double-stranded DNA fragments of ~1100 bp length with or without two *parS* sites. Fragments were generated by PCRs of a *C. glutamicum* genomic locus surrounding *parS9* and *parS10* using primer pairs parS9mut-HindIII-up-F/parS10mut-EcoRI-D-R. ParB concentrations of 0.05–25 µM were incubated with 100 ng DNA for 30 min at 30 °C, following sample separation in native gels (3–12% polyacrylamide, ServaGel^TM^). DNA was stained using SYBR® Green I (Invitrogen).

### Photoactivated localization microscopy

*C. glutamicum* cells were fixed with 3% of formaldehyde prior to super-resolution imaging using a Zeiss ELYRA P.1 microscope with laser lines HR diode 50 mW 405 nm and HR DPSS 200 mW 561 nm, and an Andor EMCCD iXon DU 897 camera. Cellular ParB-PAmCherry signals were further analyzed using Fiji software^75^ and identification of distinct protein clusters was carried out by applying the OPTICS algorithm in R^49,50^. For sample preparation, *C. glutamicum* cells expressing ParB-PAmCherry were collected in exponential growth phases, washed twice in PBS, and fixated in PBS + 3% formaldehyde solution (36.5–38% in H_2_O + 10–15% methanol, Sigma Aldrich) for 30 min at 30 °C. Excess formaldehyde was subsequently quenched by adding 10 mM glycine, cells were sedimented at 5000 × *g* for 1 min, resuspended in PBS containing 10 mM glycine, and incubated for 5 min at RT. This quenching step was repeated three times; cells were finally diluted in buffer containing 50 mM Tris pH 7.4, 50 mM NaCl, 10 mM EDTA, and 0.5 M sucrose (TSEMS). Cells expressing MksB-PAmCherry and DivIVA-mNeonGreen were not fixed due to the low amount of MksB expressed (formaldehyde fixation renders part of the fluorophore population unable to fluoresce) but simply collected and washed in TSEMS buffer.

Super-resolution imaging was performed on a Zeiss ELYRA P.1 microscope (laser lines HR diode 50 mW 405 nm and HR DPSS 200 mW 561 nm). Cellular PAmCherry-tagged proteins were detected via an Andor EMCCD iXon DU 897 camera as described before, using a long-pass 570 nm filter (LP570) and an alpha Plan-Apochromat ×100/1.46 Oil DIC M27 objective for imaging. Further, 100 nm TetraSpeck microspheres and the implemented drift correction tool served for drift correction; the Z-axis was stabilized via the “definite focus” system. PALM image calculation was performed applying the 2D *x*/*y* Gaussian fit (Zenblack software, Zeiss) using a peak mask size of 9 pixels, where one pixel corresponds to 100 nm and a peak intensity to noise ratio of 6. To exclude background and events resulting from the co-emission of co-localizing molecules, events were filtered for photon numbers between 70 and 350, and PSF (point spread function) width at 1/*e* maximum (70–170 nm) were applied. As a last step, events were grouped according to the following parameters: three on-frames with 0 off-frames allowed and a search radius of 30 nm.

When imaging strains containing ParB-PAmCherry, four imaging series were taken for each field-of view, where each subsequent serie was characterized by a specific 405 nm laser linear gradient intensity range (0.001% to 0.01%, 0.01% to 0.1%, 0.1% to 1%, and 1% to 10%). Every other imaging parameter remained the same in between the time series. The frame count for each collection was 10,000 frames and converted molecules were imaged using the 561 nm laser at 15% (transfer mode) for 50 ms at a 200-fold EMCCD gain. For MksB-PAmCherry, the 405 nm laser linear gradient ranged between 0.005% and 0.5% within 5000 frames, while the other imaging parameters were kept the same as in the case of ParB-PAmCherry. DivIVA-mNeonGreen was imaged for 10,000 frames using the 488 nm laser at 30% (transfer mode) for 50 ms at 200 EMCCD (electron multiplying charge-coupled device) gain. In this case no 405 nm laser was used, as the fluorophore is not photoactivatable. As the 488 nm laser causes activation of PAmCherry, MksB-PAmCherry was imaged prior than DivIVA-mNeongreen.

The workflow of protein cluster analysis is illustrated in Supplementary Fig. 7. The field-of-view in the bright-field channel was correct for illumination unevenness by dividing the field-of-view containing the cells of interest with an empty one (Process–Calculator Plus, Fiji) and enlarged ten times (bicubic interpolation). The resulting image was thresholded (Image–Adjust–Threshold) with default parameters and converted to a binary mask. A Fiji macro was then run on the binary mask to close the mask holes present within cells and to enlarge the cells mask themselves. Cells that were in contact with each other were separated via water shading. The perimeter coordinates corresponding to masks representing cells lying within the focus were extracted and used to exclude events originating from cells lying outside the focal plane and the background. The clustering structures of events within a cell were identified via the OPTICS algorithm in R^49,50^. OPTICS is a clustering algorithm based on two parameters: minimum points (MinPts—in this case, a point is an event) and epsilon (*ε*—maximum search radius). As, in our case, only events within the same cell can belong to the same cluster, *ε* was chosen so that it would include all the events present within each cell (*ε* = 3000 nm). The effect of the MinPts value on the visualization of the cluster-ordering has been previously described^49^. Briefly, although the overall shape of the reachability plot does not differ greatly for different MinPts, a low value makes the reachability plot more jagged, while high values smoothen it (a high value also has the benefit to decrease the chance of “single-link” effects).

As we know from epifluorescence that ParB clusters are relatively big, we chose a MinPts value (32) that would smoothen minor density variability, while being able to identify strong event density variations (Supplementary Fig. 7). The resulting reachability plot showed the presence of multiple density peaks at different reachability distance supporting the idea that within each macrocluster there are subclusters. Reachability distance thresholds were then chosen to highlight such phenomenon. Specifically, 50 and 35 nm were chosen as thresholds, as, in the tested conditions, they were able to consistently identify and separate subclusters from the macroclusters they were lying into (Supplementary Fig. 7). Documentation is available at Github (https://github.com/GiacomoGiacomelli/ParB-clustering-protein-profiling).

### Chromosome conformation capture libraries

3C/Hi-C libraries were generated as previously described by Val et al.^48^ with minor changes. Briefly, cells were grown in 200 ml of BHI medium at 30 °C to an OD_600_ of 3 and rediluted to a final concentration of ~1 × 10^7^ cells/ml. Cells were crosslinked using fresh formaldehyde for 30 min at RT (3% final concentration; Sigma Aldrich Formalin 37%) followed by 30 min at 4 °C. Formaldehyde was quenched using a final concentration of 0.25 M glycine for 20 min at RT. Cells were then collected by centrifugation, frozen in dry ice, and stored at −80 °C until use. Frozen pellets of ~10^9^ cells were thawed on ice and suspended in a final volume of 1.1 mL 1× TE (pH 8) and transferred in a VK01 Precellys Tube (beads beating). Fixed cells were disrupted using the following program on a precellys apparatus: 9 cycles × [20“ − 3500 r.p.m.; 30“ −  pause]. Lysate was transferred to a 1.5 ml tube, SDS 10% was added to the mix to a final concentration of 0.5% and the mix was incubated for 10 min at RT. 1 ml of lysate was then transferred in a 5 ml tube containing 4 ml of digestion mix (1× NEB 3 buffer, 1% Triton X-100, and 1000 U MluCI enzyme). DNA was digested for 3 h at 37 °C under shaking. Insoluble fraction was then recovered through centrifugation (16,000 × *g*, 20 min) and the obtained pellet was resuspended in 1 ml of water and diluted in 15 ml of ligation reaction mix (1× ligation buffer NEB without ATP, 1 mM ATP, 0.1 mg/ml bovine serum albumin, 125 Units of T4 DNA ligase 5 U/ml). Ligation was allowed to proceed for 4 h at 16 °C, followed by incubation overnight at 65 °C in the presence of 250 mg/ml proteinase K, 0.5% SDS, and 5 mM EDTA. Next morning, DNA was precipitated using 1/10th volume of 3 M Na-Acetate (pH 5.2) and one volume of isopropanol. After 1 h at –80 °C, DNA was pelleted, resuspended in 900 µl 1× TE buffer, and extracted with 900 µl phenol–chloroform pH 8.0. DNA was again precipitated using 1/10th volume of 3 M Na-Acetate (pH 5.2) and 2.5 volume of cold Ethanol. Finally, DNA was resuspended in 100 µl 1× TE buffer supplemented with RNAse and incubated 30 min at 37 °C. 3C libraries were then processed as described^48^ and paired-end sequenced on an Illumina NextSeq apparatus (2 × 35 bp). DNA content per cell was further determined by flow cytometry, yielding an average number of six chromosome equivalents per cell for all strains analyzed by Hi-C like approaches (Supplementary Fig. 2B, C and Supplementary Table 2).

### Contact map generation

Contact maps were generated as previously described^38^. Reads were aligned independently (forward and reverse) using Bowtie 2 in local and very sensitive mode and were assigned to a restriction fragment. Non-informative events (self-circularized DNA fragments, or uncut fragments) were discarded by taking into account the pair-reads relative directions and the distribution of the different configurations as described in Cournac et al.^81^. We then bin the genomes into regular units of 5 Kb to generate contact maps and normalized them using the sequential component normalization procedure^81^. Contact maps were then generated using Pyplot library and a saturation threshold at 99.5% of the maximum value.

### Contact map comparison

Ratio between contact maps was computed for each point of the map by dividing the amount of normalized contacts in one condition by the amount of normalized contacts in the other condition and by plotting the Log2 of the ratio. The color code reflects a decrease or increase of contacts in one condition compared to the other (blue or red signal, respectively). No change is represented by a white signal. To further compare the Hi-C data, we applied the HiCRep software^56^ at a resolution of 5 Kb with a smoothing index of 3.

### Identification of domains frontiers using directional index

To quantify the degree of directional preference, we applied on correlation matrices the same procedure as in Marbouty et al.^10^. For each 5 Kb bin, we extracted the vector of interactions from the correlation matrix between the studied bin and bins at regular 5 Kb intervals, up to 250 Kb in left and right directions. The two vectors were then compared with a paired *t*-­test to assess their statistical significant difference (*p* = 0.05). The directional preferences for the bin along the chromosome are represented as a bar plot with positive and negative t­values shown as red and green bars.

### Flow cytometry

DNA contents per cell were verified in *C. glutamicum* strains analyzed by 3C using flow cytometry as described before^41^ Flow cytometry analysis was performed as described before^41^. In short, exponentially growing cultures were treated with 25 µg/ml chloramphenicol for more than 4 h, to induce replication runouts. Cells were fixated in 70% ethanol (1:9 v/v) and washed once in PBS. Cellular DNA was stained using SYBR® Green I (Invitrogen, 1:10,000 dilutions) for 15 min in the dark. Flow cytometry analysis was carried out subsequently using a BD Accuri C6 (BD Biosciences) equipped with a 488 nm laser. At least 200,000 events were collected per sample at a slow flow rate of 10 µl/min measuring <5000 events per second, applying an acquisition threshold of 650 set on the green channel FL1-H. Data analysis was performed using the BD Accuri C6 Plus software (BD Biosciences). At first, events derived from cell aggregates were identified in plots of SSC vs. width and excluded from DNA content analysis. Remaining events were plotted as histograms vs. DNA amount (FL1-A, EM BP 533/30) at log scale, where chromosome numbers were assigned in accordance to calibration standards described before^41^. All experiments were performed in biological triplicates.

### Comparison of contact signals with transcriptional data

RNA-sequencing data for *C. glutamicum* were recovered from ENA (Project PRJEB4788). Only reads with a mapping quality above 30 were conserved. Raw signal was then binned to match the binning of the corresponding contact maps and plotted along the genome. Both contact and transcription signals were smoothed with a Savitzky-Golay filter as previously described in Lioy et al.^38^.

### Statistics and reproducibility

Correlation coefficients, linear regressions and analyses of nearest neighbor distance distributions were calculated using Excel 2019, Graph Pad Prism (GraphPad Software), and R (R-studio v1.1.453, R version v3.5.0)^50^.

Micrographs contained in the main and supplementary figures are exemplary images from three biological replicates. Plasmid extractions in Fig. 5f were repeated three times; control western blottings in Supplementary Fig. 8A were performed once, and gels in Supplementary Fig. 12B are exemplary data from two replicates.

### Reporting summary

Further information on research design is available in the Nature Research Reporting Summary linked to this article.

## Supplementary information

Supplementary Information

Description of Additional Supplementary Files

Supplementary Data 1

Supplementary Data 2

Reporting Summary

## Data Availability

*C. glutamicum* ATCC 13032 genome sequence was obtained from GeneBank (GeneBankID: BX927147.1). RNA-seq data for *C. glutamicum* were recovered from ENA (Project PRJEB4788). Proteomic data are available via ProteomeXchange with the project identifier PXD008916 (ref. ^82^). Genome-wide sequencing reads of ChIP-seq and chromosome conformation capture assays generated in this study are available in the Sequence Read Archive (SRA) under accession numbers PRJNA529385 and PRJNA525583. Flow cytometry raw data results were deposited in the FlowRepository database (accession number FR-FCM-Z2DJ)^80^. The source data underlying Figs. 2c, h, 3b, d, e, and 5b, c, e, f and Supplementary Figs. 2B, C, 3B–D, 5A, D, 7C–E, 8A–C, 9B–D, 10A, B, D, 12A, B, D, and 13A, C are provided as a Source Data file.

## Source Data

Source Data

## Code availability

The custom computer algorithm used in the analysis of PALM data has been deposited in Github (https://github.com/GiacomoGiacomelli/ParB-clustering-protein-profiling).
