# Supplementary material for: Chromosome organization by a conserved condensin-ParB system in the actinobacterium Corynebacterium glutamicum
Source: Nat Commun. 2020 Mar 20;11:1485. doi: 10.1038/s41467-020-15238-4 (PMC7083940; doi:10.1038/s41467-020-15238-4)
Supplement: Supplementary file 1 — Supplementary Information [file 41467_2020_15238_MOESM1_ESM.pdf]

Supplementary Information

Chromosome organization by a conserved condensin-ParB system in the actinobacterium *Corynebacterium glutamicum*

Kati Böhm[1], Giacomo Giacomelli[1,2], Andreas Schmidt[3], Axel Imhof[3], Romain Koszul[4,5], Martial Marbouty[4]*, and Marc Bramkamp[1,2]*

[1] Ludwig-Maximilians-Universität München, Fakultät Biologie, Großhaderner Straße 2-4, 82152 Planegg-Martinsried, Germany

[2] Christian-Albrechts-Universität zu Kiel, Institut für allgemeine Mikrobiologie, Am Botanischen Garten 1-9, 24118 Kiel, Germany

[3] Ludwig-Maximilians-Universität München, Zentrallabor für Proteinanalytik, Biomedizinisches Centrum München, 82152 Planegg-Martinsried, Germany

[4] Institut Pasteur, Unité Régulation Spatiale des Génomes, Paris, France/ CNRS, UMR 3525, Paris, France.

[5] Institut Pasteur, Center of Bioinformatics, Biostatistics and Integrative Biology (C3BI), Paris, France

* Corresponding authors:

Marc Bramkamp: Email: bramkamp@ifam.uni-kiel.de; Phone: +49-(0)431-8804341; Fax: +49(0)431-880-2198

Martial Marbouty: Email: martial.marbouty@pasteur.fr; Phone: +33-144389365

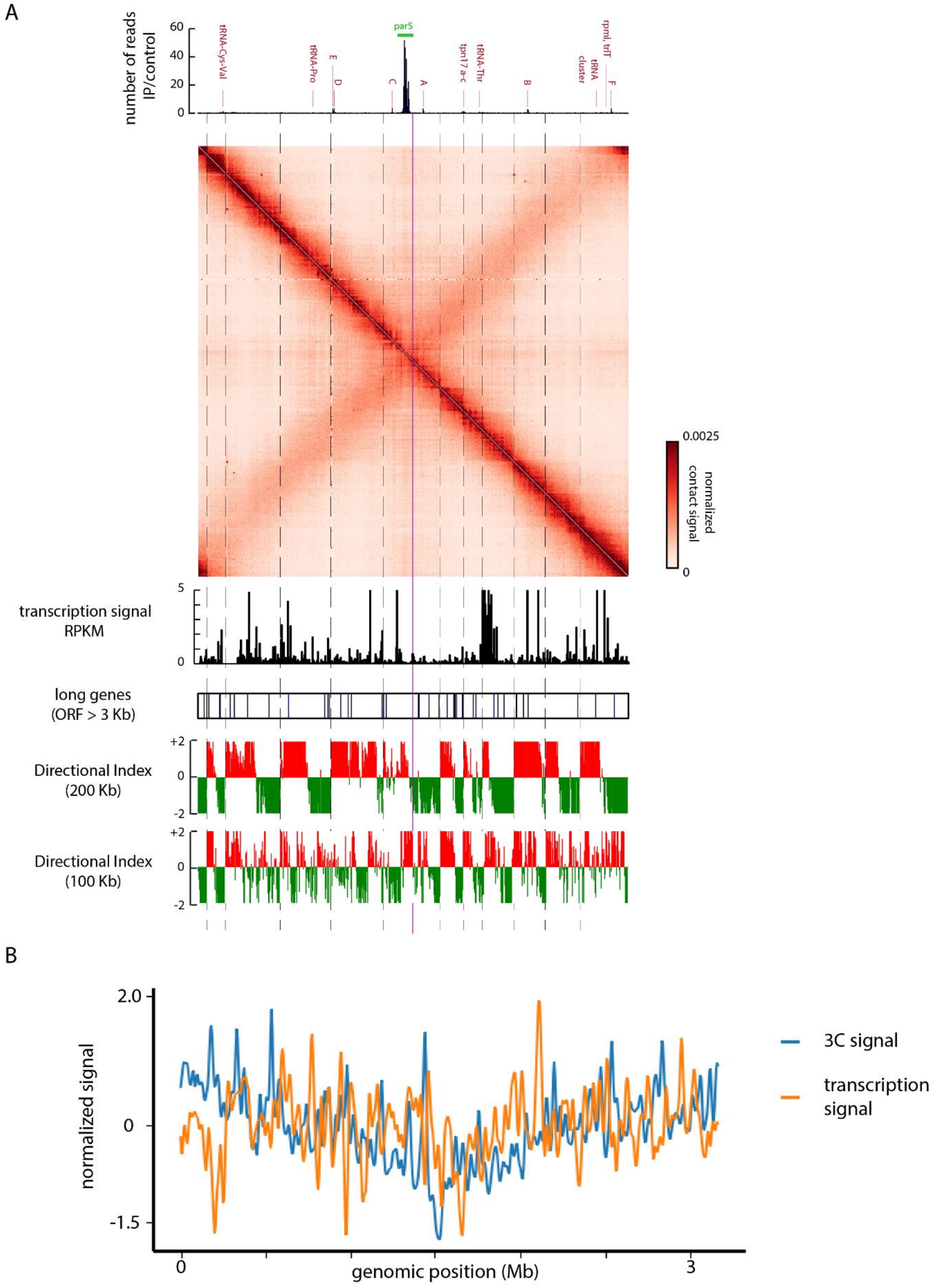
A
number of reads IP/control
60
40
20
0
parS
tRNA-Cys-Val
tRNA-Pro
E
D
C
A
tpn17 a-c
tRNA-Thr
B
tRNA cluster
rpmI, trlT
F
0.0025
normalized contact signal
0
transcription signal RPKM
5
0
long genes (ORF > 3 Kb)
Directional Index (200 Kb)
+2
0
-2
Directional Index (100 Kb)
+2
0
-2
B
normalized signal
2.0
0
-1.5
genomic position (Mb)
0
3
3C signal
transcription signal

Supplementary Figure 1: Chromosome organization and domains analysis in *Corynebacterium glutamicum*.

A) From top to bottom: i) Whole genome anti-mCherry ChIP-seq data of strain CKB006 harboring ParB-mCherry. ii) Normalized genomic contact map derived from asynchronously grown cells (fast growth $\mu \geq 0.6\ h^{-1}$, exponential phase). X- and Y-axes indicate chromosomal coordinates binned in 5 Kb; *oriC*-centered as in Fig. 1. Color scales, indicated above the contact map, reflect contact frequency between two genomic loci from white to red (rare to frequent contacts). iii) Transcription signal using 5Kb bins [1]. iv) long genes (black bars) more than 3 Kb. v) domain signals at 200 Kb resolution (DI analysis) with up- and downstream regions marked in green and red are displayed. vi) domain signals at 100 Kb resolution. **B)** Correlation between transcription and short-range chromosome contacts captured by 3C. Genomic coordinates are indicated on X-axis and contacts between neighboring bins are plotted (blue curve) along the transcription pattern (orange curve) [2].

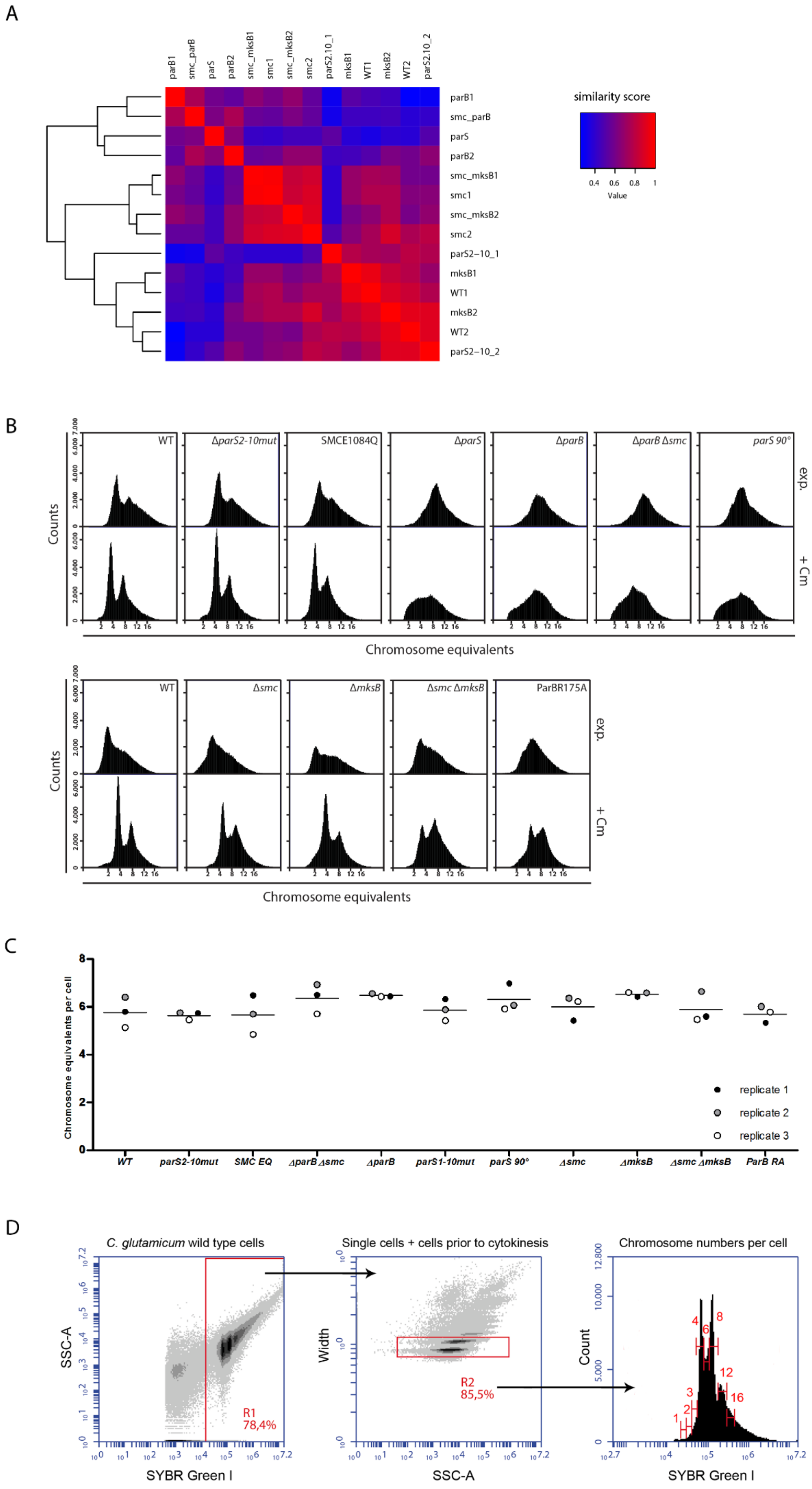
A
parB1
smc_parB
parS
parB2
smc_mksB1
smc1
smc_mksB2
smc2
parS2.10_1
mksB1
WT1
mksB2
WT2
parS2.10_2
parS2−10_1
parS2−10_2
similarity score
0.4 0.6 0.8 1
Value
B
Counts
WT
ΔparS2-10mut
SMCE1084Q
ΔparS
ΔparB
ΔparB Δsmc
parS 90°
exp.
+ Cm
Chromosome equivalents
Δsmc
ΔmksB
Δsmc ΔmksB
ParBR175A
C
Chromosome equivalents per cell
WT
parS2-10mut
SMC EQ
ΔparB Δsmc
ΔparB
parS1-10mut
parS 90°
Δsmc
ΔmksB
Δsmc ΔmksB
ParB RA
replicate 1
replicate 2
replicate 3
D
C. glutamicum wild type cells
Single cells + cells prior to cytokinesis
Chromosome numbers per cell
SSC-A
Width
Count
SYBR Green I
R1
78,4%
R2
85,5%

Supplementary Figure 2. Replicates of chromosomal contact maps in *C. glutamicum* mutants.

A) Similarity matrix between the different constructed libraries. The distance between the different libraries was calculated using HiCRep [3]. Color scale indicates the similarity score between libraries. **B)** Determination of DNA amount per cell of *C. glutamicum* strains used for Hi-C. Distributions of DNA content in wild-type and mutant strains as indicated (top: CBK023, CBK050, CBK024, CDC003, CBK002, CBK037; below: CDC026, CBK001, CBK004, CBK047) Flow cytometry was performed with SYBR Green-stained samples of exponential BHI-grown cultures (exp.) and after > 4 h of incubation with chloramphenicol (+Cm). Chromosome numbers are shown in histograms vs the green channel Fl1-A. **C)** Averaged chromosome numbers per cell determined by flow cytometry after replication runout for above-named strains (mean, n = 3). DNA amount per cell does not significantly differ between wild-type and mutant strains (two sample t test, 5 % significance level). **D)** Gating strategy for *C. glutamicum* cells via flow cytormetry. Gating is exemplified using wild-type *C. glutamicum* cells at replication run-out conditions. Left: 200000 cells were monitored in R1 in order to distinguish DNA-containing cells (SYBR Green I^+) from dust particles and non-stained debris using SYBR Green I versus side scatter (SSC-A) gating. Middle panel: Cell aggregates derived from fixation processes and incomplete separation after cell division were excluded from R1 in SSC-A versus width plots in gate R2. Note that two populations were taken into account in order to include single cells as well as long cells prior to cytokinesis. Right: Histogram showing distribution of SYBR Green I^+ cells (labels represent increasing number of chromosome equivalents) within gate R2. Source data are provided as a Source Data file.

A

parS$_{WT}$	TGTTTCACGTGAAACA	PmlI
parS$_{mut}$		
1	GGTGTCCCGGGAGACT	XmaI
2-4, 6-8, 10	CGAATCCCGGGATACT	
5, 9	CGAATGTCGACATACT	SalI

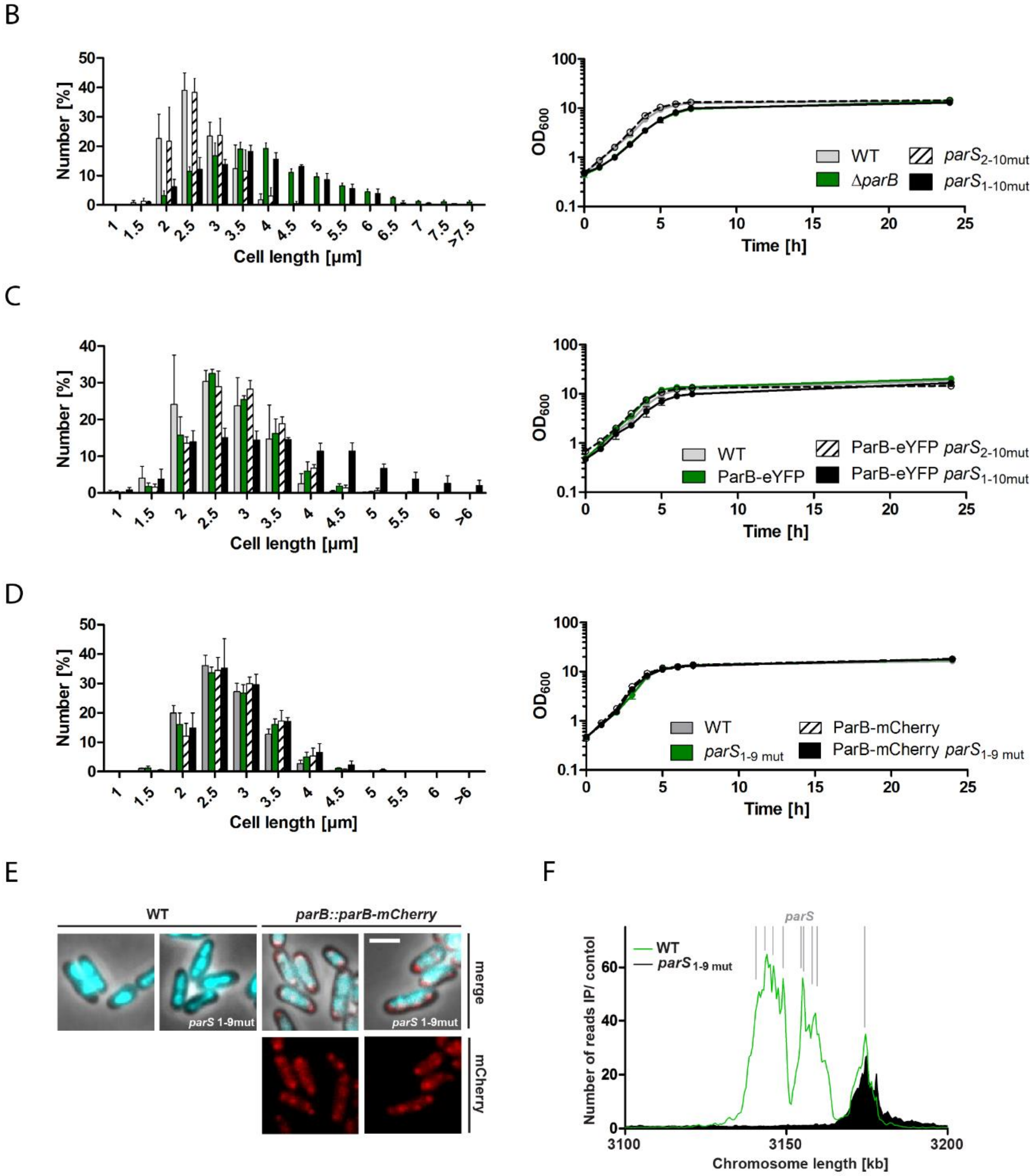

Supplementary Figure 3. Mutation and shifting of *parS* sites affects growth and morphology.

A) Point mutations render native *parS* sequences nonfunctional. Base pair exchanges (red letters) generate new restriction sites (underscored) for mutation screening as indicated. Notably, point mutations of the intragenic *parS* site are silent. **B), C)** Mutation of *parS* sites mimics growth and morphology of *ΔparB* cells. Comparison of cell length distributions (mean +/- s.d., n of each biological replicate listed below) and growth curves (mean +/- s.d., n = 3) of *parS* mutants and *parB* deletion strain grown in BHI. Growth rates derive from triplicates: μ_{WT}=0.65 h^{-1} (n_{b1} = 446, n_{b2} = 387, n_{b3} = 355, n_{c1} = 633, n_{c2} = 381, n_{c3} = 325) , $\mu_{\Delta parB}$ =0.57 h^{-1} (CDC003) (n_1 = 376, n_2 = 628, n_3 = 518), $\mu_{parS\text{2-10mut}}$=0.69 h^{-1} (CBK023) (n_1 = 503, n_2 = 502, n_3 = 952), $\mu_{parS\text{1-10mut}}$=0.57 h^{-1} (CBK024) (n_1 = 383, n_2 = 261, n_3 = 506), $\mu_{\text{ParB-eYFP}}$=0.65 h^{-1} (CBK007) (n_1 = 421, n_2 = 403, n_3 = 487), $\mu_{\text{ParB-eYFP } parS\text{2-10mut}}$=0.59 h^{-1} (CBK025) (n_1 = 710, n_2 = 408, n_3 = 302), $\mu_{\text{ParB-eYFP } parS\text{1-10mut}}$=0.55 h^{-1} (CBK026) (n_1 = 402, n_2 = 209, n_3 = 257). **D)** Growth curves (mean +/- s.d., n = 3) and cell length measurements (mean +/- s.d., n listed below) for *C. glutamicum* wild-type (WT, µ=0.68) (n_1 = 1545, n_2 = 1247, n_3 = 1170), *parS*1-9mut (CBK90, µ=0.69) (n_1 = 2300, n_2 = 975, n_3 = 959), *parB::parB-mCherry* (CBK006, µ=0.68) (n_1 = 1427, n_2 = 1772, n_3 = 884), *parB::parB-mCherry parS*1-9mut (CBK091, µ=0.69) (n_1 = 670, n_2 = 576, n_3 = 734). **E)** Fluorescence microscopy of above-named strains. ParB-mCherry fluorescence (red) is shown in separate channels and as overlays with bright field and Hoechst fluorescence (DNA stain, cyan). Scale bar; 2µm. **F)** ParB-mCherry ChIP-seq in strain CBK0 harboring a single *parS10* site (black) compared to wild-type (green). Enrichment signals are shown in a 0.1 Kb range surrounding the *parS* cluster; chromosomal *parS* positions are indicated by gray lines. Source data are provided as a Source Data file.

A

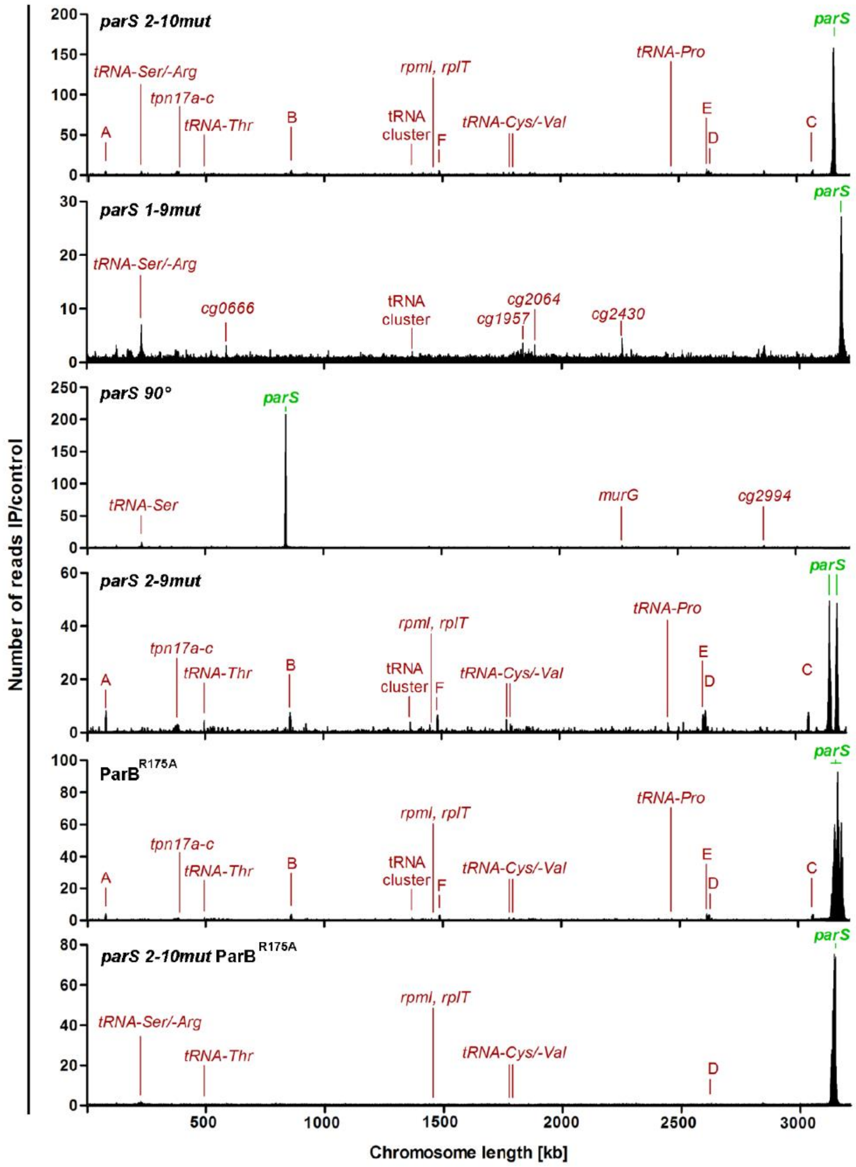

parS 2-10mut
parS 1-9mut
parS 90°
parS 2-9mut
ParB^{R175A}
parS 2-10mut ParB^{R175A}
parS
tRNA-Ser/-Arg
tpn17a-c
tRNA-Thr
tRNA cluster
rpmI, rplT
tRNA-Cys/-Val
tRNA-Pro
cg0666
cg1957
cg2064
cg2430
tRNA-Ser
murG
cg2994
A
B
C
D
E
F
Number of reads IP/control
Chromosome length [kb]

B

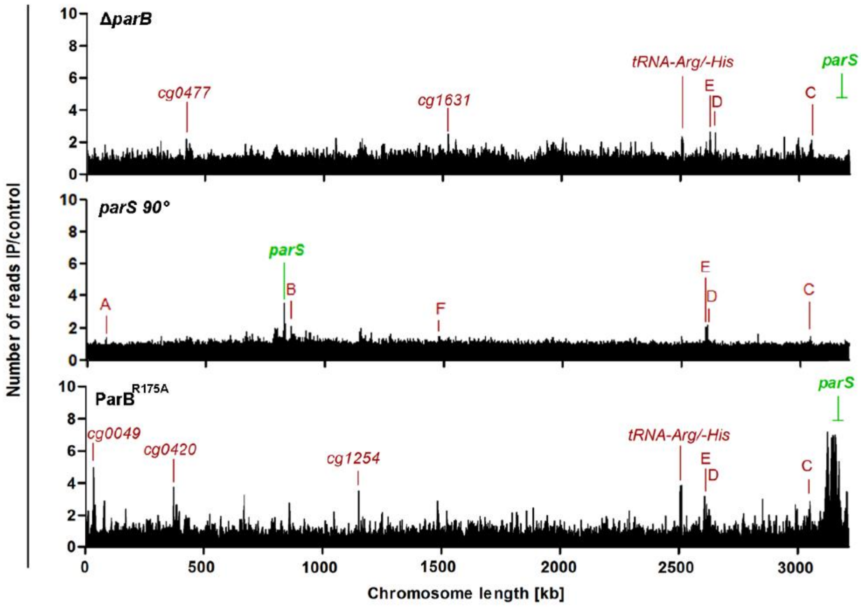

ΔparB
parS 90°
ParB^{R175A}
parS
cg0477
cg1631
tRNA-Arg/-His
cg0049
cg0420
cg1254
A
B
C
D
E
F
Number of reads IP/control
Chromosome length [kb]

Supplementary Figure 4. Whole genome ParB- and SMC-ChIP-seq data.

A) ParB -ChIP-seq analyses in different mutant backgrounds; enrichments at *parS* sites (green) and highly transcribed genes (red) are depicted with a bin size of 0.5 Kb. Fold enrichments of immunoprecipitation relative to extract samples (IP/control) are shown for the entire genome lengths, respectively; x-axes are centered at *terC*. Strains analyzed from top down are: *parB::parB-mCherry parS*$_{2\text{-}10\text{mut}}$ (CBK029), *parB::parB-mCherry parS*$_{1\text{-}9\text{mut}}$ (CBK091), *parB::parB-mCherry parS* at 90° chromosomal position (*3' cg0904*, CBK042), *parB::parB-mCherry parS*$_{2\text{-}9\text{mut}}$ (CBK030), *parB::parB*^{R175A}*-mCherry* (CBK47) and *parB::parB*^{R175A}*-mCherry parS*$_{2\text{-}10\text{mut}}$ (CBK048). **B)** DNA-enrichment patters were determined for SMC via ChIP-seq as described above. Genotypes analyzed are *smc::smc-mCherry* in combination with either Δ*parB* (CBK014), *parS* at 90° chromosomal position (3' of *cg0904*, CBK045) or *parB::parB*^{R175A} (CBK049).

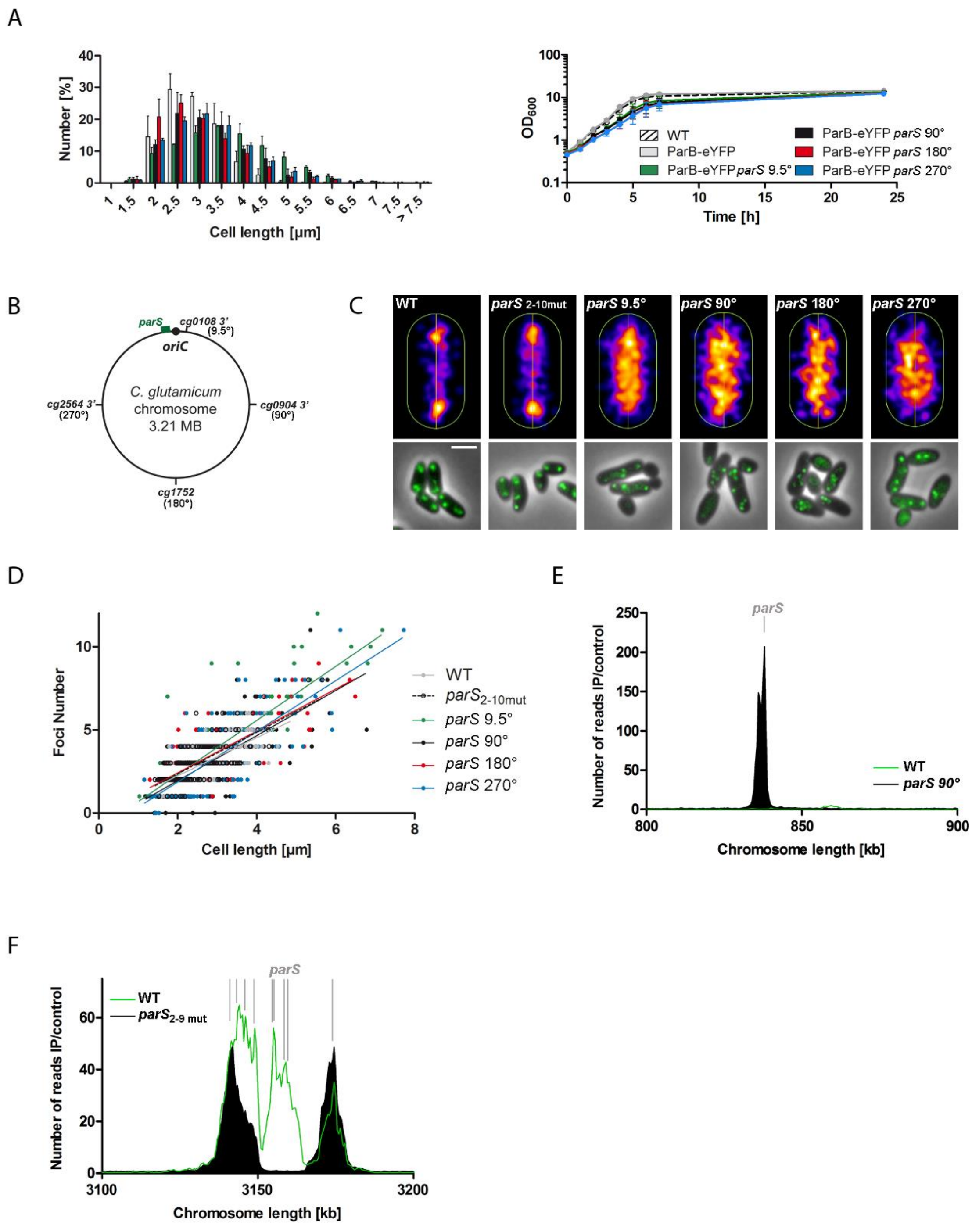

Supplementary Figure 5. Confined chromosomal positioning of *parS* impacts on its function.

A) Cell lengths (mean+/- s.d., n for each replicate listed below) and growth curves (mean+/- s.d., n=3) determined for mutant cells ParB-eYFP in combination with a single misplaced *parS* site at different chromosomal location in degree, growth rates:

μ_{WT}=0.57 h^{-1} (n_1 = 410, n_2 = 276, n_3 = 286), $\mu_{ParB\text{-}eYFP}$=0.61 h^{-1} (CBK007) (n_1 = 1126, n_2 = 1126, n_3 = 378), $\mu_{ParB\text{-}eYFP\ parS9.5°}$ =0.46 h^{-1} (CBK040) (n_1 = 549, n_2 = 468, n_3 = 271), $\mu_{ParB\text{-}eYFP\ parS\ 90°}$ =0.46 h^{-1} (CBK041) (n_1 = 527, n_2 = 527, n_3 = 252), $\mu_{ParB\text{-}eYFP\ parS180°}$ =0.40 h^{-1} (CBK044) (n_1 = 415, n_2 = 498, n_3 = 363), $\mu_{ParB\text{-}eYFP\ parS\ 270°}$ =0.41 h^{-1} (CBK043) (n_1 = 414, n_2 = 409, n_3 = 311).**B)** Scheme of chromosomal *parS* insertions, native *parS* cluster shown in green, *parS* shifted to intergenic regions 3' of *cg0108*, 3' of *cg0904*, 3' of *cg2563* or *cg1702::parS* (CBK040, CBK041, CBK043, CBK044). **C)** ParB recruitment to *parS* sites at any chromosomal position remains intact. Top: average ParB-*parS* cluster localizations of *parB::parB-eYFP* cells with native *parS* cluster (WT, CBK007) or one single *parS* site (*parS* $_{2\text{-}10mut}$, CBK025) and *parS* sequences located at respective chromosomal loci illustrated via MicrobeJ [4] Below: ParB-eYFP clusters (green) of representative mutant cells harboring *parS* sites at above-mentioned chromosomal positions. Scale bar, 2 µm. **D)** ParB foci number in relation to cell length in *parS*-shifted mutant strains (n>150). Linear regression lines are shown, r(WT)=0.71, r(*parS* $_{2\text{-}10mut}$)=0.74, r(*parS 9.5°*)=0.86, r(*parS 90°*)=0.68, r(*parS 180°*)=0.75, r(*parS 270°*)=0.79. **E)** α-mCherry-ChIP-seq of *C. glutamicum parB::parB-mCherry parS* at 90° chromosomal position (black, CBK042) compared to wild-type enrichment signal (green, CBK006) within genomic region 0.8 -0.9 Mb including mutant *parS* location (gray), bin size 0.5 Kb. **F)** ChIP-seq of cells harboring *parS*1 and 10 (*parS* $_{2\text{-}9mut}$, CBK30), performed and compared to the wild-type-signal as in E). Source data are provided as a Source Data file.

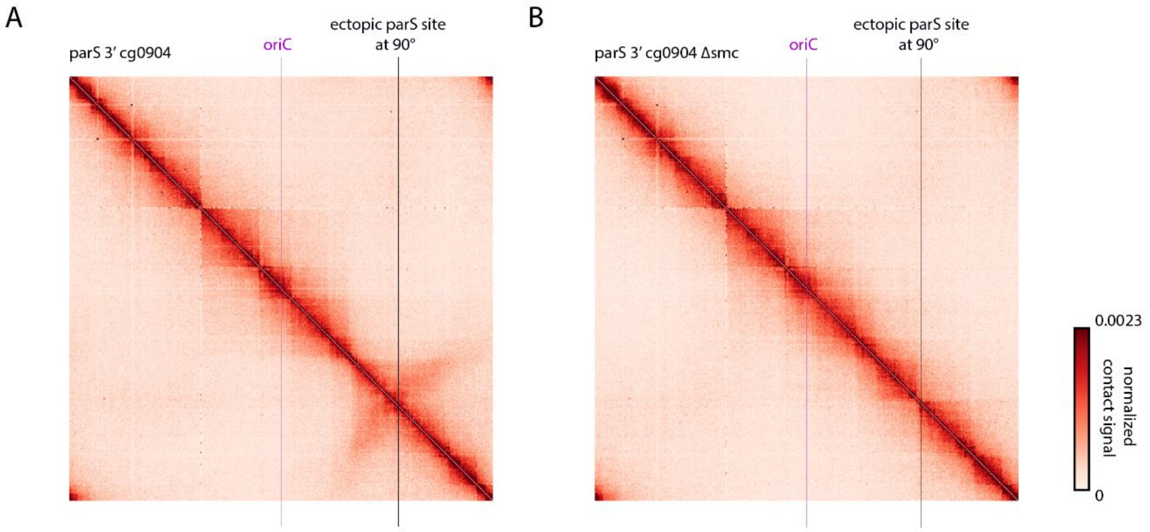

Supplementary Figure 6. Whole chromosome organization of *C. glutamicum* mutants.

Normalized contact maps of mutant strains. **A)** Chromosomal contacts of *parS* at 90° chromosomal position in a wild-type (CBK037). **B)** Chromosomal contacts of *parS* at 90° chromosomal position in a Δ*smc* background (CBK046). Contact maps are *ori* centered (purple lines).

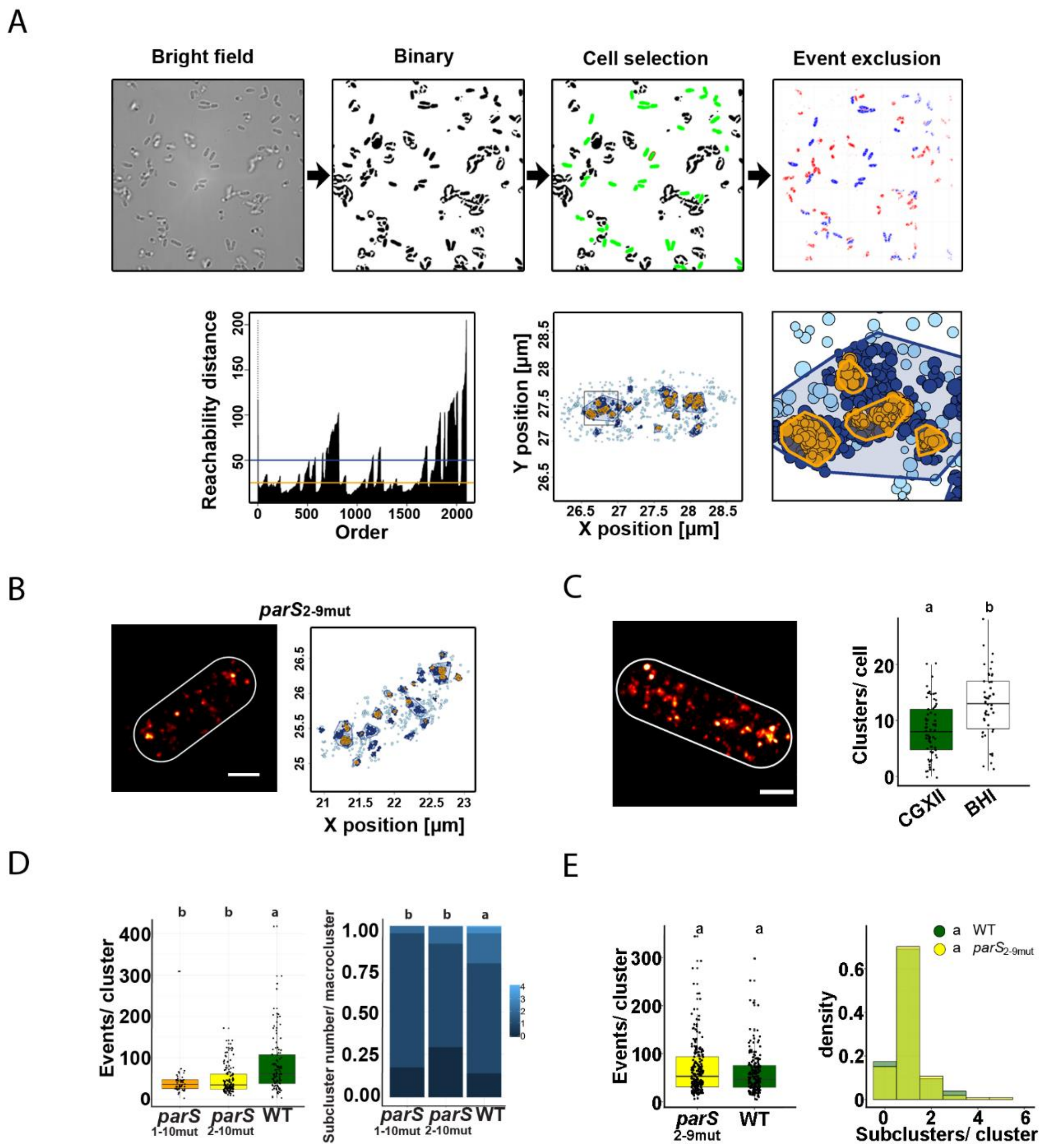

Supplementary Figure 7. PALM analyses on cellular ParB localization.

A) Workflow for protein cluster analysis. **B)** Representative *parS*$_{2\text{-}9mut}$ mutant cell (CBK031) analyzed via single molecule localization microscopy. Left: Gaussian rendering of ParB-PAmCherry signals, right: color-coded representation of ParB-PAmCherry events within corresponding cells as described in Fig. 2G. **C)** Left: Gaussian rendered PALM microscopy image (0.71 PSF, 1 px = 10 nM) exemplifying ParB-PAmCherry localization in fast-grown *C glutamicum* cell, strain CBK009. Scale

bars, 0.5 µm. Right: more ParB-PAmCherry macroclusters are present in fast-growing (BHI medium) compared to slow-growing cells (CGXII medium), (significance by two-tailed Kruskal-Wallis Rank Sum Test: chi-squared = 14.576, df = 1, p = 0.0001346, n_{BHI} = 47, n_{CGXII} = 68). Medians are indicated as solid lines and whiskers mark 1.5 IQR (Inter Quartile Ranges). **D)** Properties of the two biggest ParB-PAmCherry clusters per cell compared between strains harboring all (wild-type, WT), a single *parS* 1 site (1 *parS*) or no *parS* site (wo *parS*, CBK087) ($clusters_{wild\text{-}type}$: n = 114, $clusters_{parS2\text{-}10mut}$: n = 145, $clusters_{parS1\text{-}10mut:}$ n = 46). Left: two-tailed Kruskal-Wallis Rank Sum test yields significant differences in number of events per macro-cluster of wild-type cells and cells harboring one *parS* (chi-squared = 27.582, df = 1, p-value = 1.506e-07) or no *parS* site (chi-squared = 20.167, df = 1, p-value = 7.096e-06), as indicated by letters above data sets. Medians are indicated as solid lines and whiskers mark 1.5 IQR. Right: Quantity of ParB sub-clusters per macro-cluster depends on number of *parS* sites. Data sets differ significantly between wild-type and cells harboring a single *parS1* (two-tailed Kruskal-Wallis Rank Sum test: chi-squared = 12.284, df = 1, p-value = 0.0004569) or no *parS* site (two-tailed Kruskal-Wallis Rank Sum test: chi-squared = 4.6317, df = 1, p-value = 0.03139). **E)** Comparison of the two biggest ParB-PAmCherry clusters in wild-type and $parS_{2\text{-}9mut}$ ($n_{wildtype}$ = 178, $n_{parS2\text{-}9mut}$ = 205). Two-tailed Kruskal-Wallis Rank Sum test yielded no significant differences of macrocluster sizes (medians are indicated as solid lines and whiskers mark 1.5 IQR) (left, chi-squared = 1.7848, df = 1, p = 0.1816) or subcluster numbers (right, chi-squared = 0.38145, df = 1, p = 0.5368) amongst both strain backgrounds. Source data are provided as a Source Data file.

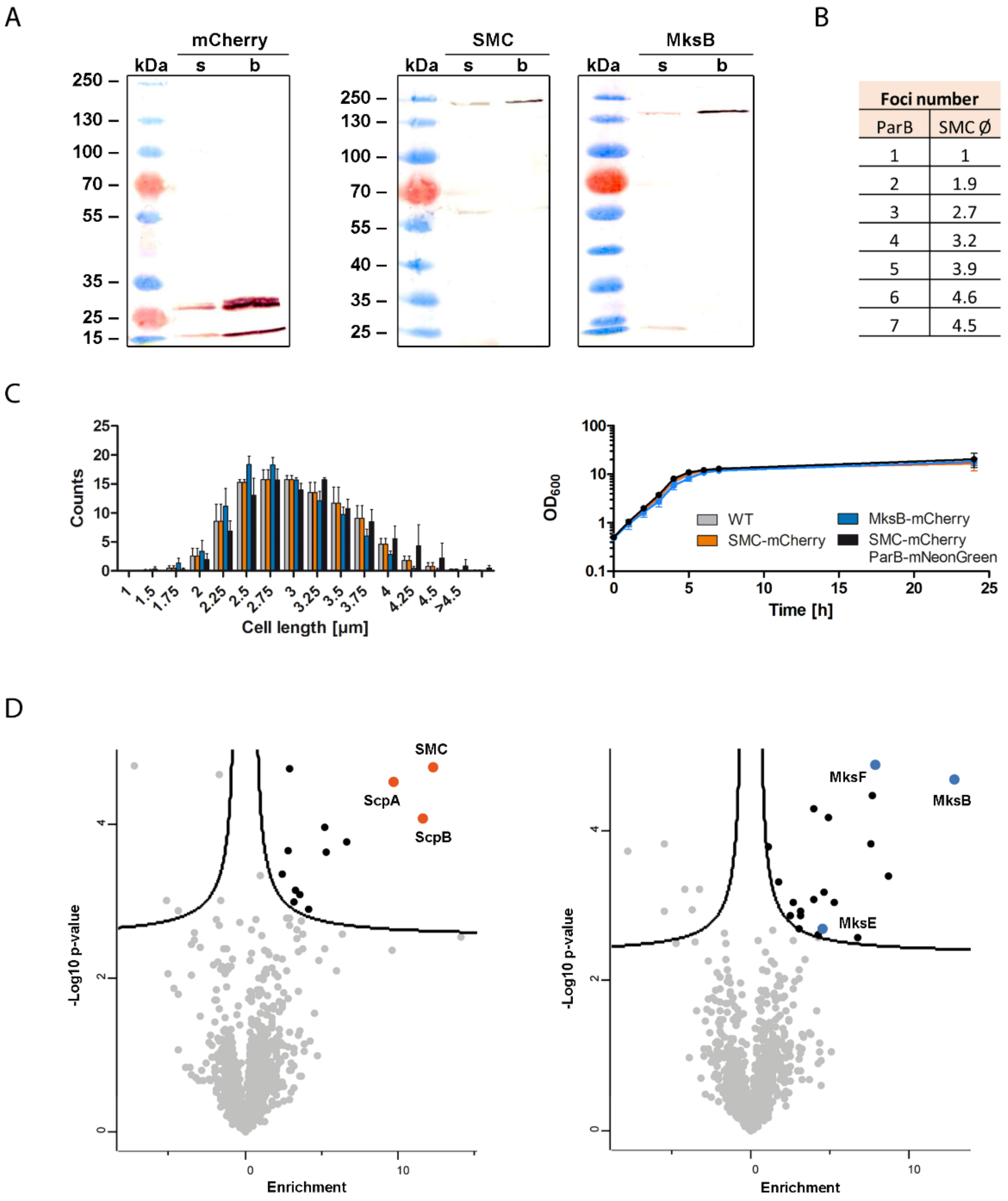

Supplementary Figure 8. Validation of full-length fusion proteins SMC-/ MksB-mCherry and co-immunoprecipitation of subunits.

A) Full length fluorescent fusion proteins and their enrichment during immunoprecipitation were validated via western blotting. Whole cell lysates of *C. glutamicum* strains CBK052 (pEKEx2-mCherry), CBK012 (*smc::smc-mCherry*) and CBK015 (*mksB::mksB-mCherry*) strains were used for pulldown experiments. Western blots show proteins SMC-mCherry (155 kDa), MksB-mCherry (151 kDa) and mCherry (26.7 kDa) trapped on 10 µl magnetic RFP-Trap® beads (b) and in the 10 µl

supernatant (s) detected via polyclonal α-mCherry antibody. **B)** Average SMC foci numbers increase in dependence of ParB- clusters per cell in strain *smc∷smc-mCherry parB∷parB-mNeonGreen* (CBK013), n>200. **C)** Growth curves (mean +/- s.d., n = 3) and cell length distributions (mean +/- s.d., n>1000) of *C. glutamicum* strains grown in BHI harboring allelic replacements of condensin subunits or ParB by fluorescent versions as indicated. μ_{WT}=0.64 h^{-1}, $\mu_{smc\text{-}mCherry}$=0.67 h^{-1} (CBK012), $\mu_{mksB\text{-}mCherry}$=0.60 h^{-1} (CBK015) $\mu_{smc\text{-}mCherry\ parB\text{-}mNeonGreen}$=0.68 h^{-1} (CBK013). Source data are provided as a Source Data file. **D)** Condensin subunit interactions identified by pull-down experiments and mass spectrometry. Cell lysates of *C. glutamicum smc∷smc-*mCherry, *mksB∷mksB-mCherry* and a negative control strain (CBK052) were used for co-immunoprecipitations via anti-mCherry agarose beads. Volcano plots show the difference in means (enrichment) plotted against the -log10 adjusted p value for each protein identified by mass spectrometry in three independent samples; condensin subunits are highlighted respectively. The cutoff curves indicate significant hits (two-tailed t-test, p<0.05, FC >0.1).

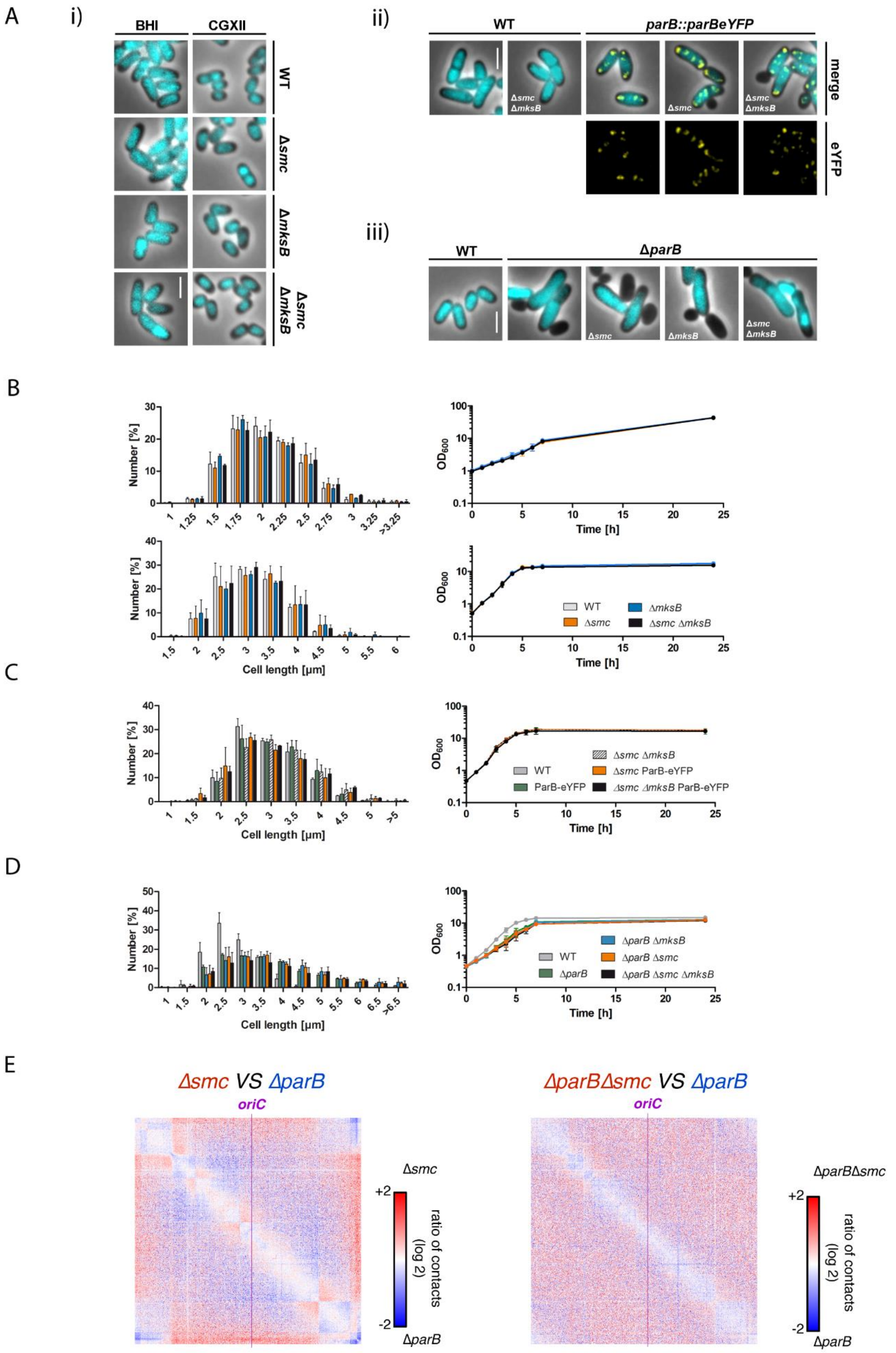
A
i)
BHI
CGXII
WT
Δsmc
ΔmksB
Δsmc ΔmksB
ii)
WT
parB::parBeYFP
merge
eYFP
iii)
WT
ΔparB
Δsmc
ΔmksB
B
Number [%]
OD600
Time [h]
Cell length [µm]
WT
Δsmc
ΔmksB
Δsmc ΔmksB
C
ParB-eYFP
Δsmc ParB-eYFP
Δsmc ΔmksB ParB-eYFP
D
ΔparB
ΔparB ΔmksB
ΔparB Δsmc
ΔparB Δsmc ΔmksB
E
Δsmc VS ΔparB
ΔparBΔsmc VS ΔparB
oriC
ratio of contacts (log 2)
+2
-2

Supplementary Figure 9. Characterization of growth and cell length phenotypes of condensin deletion strains.

A) Phenotypes of exponentially grown *C. glutamicum* mutant cells exemplified in overlays of ParB-eYFP fluorescence (yellow) with Hoechst-stained DNA (cyan) and the phase contrast image, respectively. The eYFP fluorescence channel is additionally depicted in separate images. Scale bar, 2 µm. i) Wild-type and mutant strains CDC026, CBK001, CBK004 grown in CGXII and BHI medium. ii) Wild-type and strains CBK004, CBK007, CBK010, CBK011 and iii) Wild-type, CDC003, CBK002, CBK003, CBK005 grown in BHI medium. **B)-D)** Growth curves (mean +/- s.d., n = 3) and cell length distributions (mean +/- s.d., n > 1000) of *C. glutamicum* mutant strains displayed above. **B)** Growth experiments performed in CGXII medium (top): μ_{WT}=0.26 h^{-1}, $\mu_{\Delta smc}$=0.22 h^{-1} (CDC026), $\mu_{\Delta mksB}$=0.27 h^{-1} (CBK001), $\mu_{\Delta smc\ \Delta mksB}$=0.25 h^{-1} (CBK004); in BHI medium (below): μ_{WT}=0.71 h^{-1}, $\mu_{\Delta smc}$=0.70 h^{-1}, $\mu_{\Delta mksB}$=0.72 h^{-1}, $\mu_{\Delta smc \Delta mksB}$=0.70 h^{-1}. **C)** Analysis in BHI medium: μ_{WT}=0.70 h^{-1}, $\mu_{ParB\text{-}eYFP}$=0.69 h^{-1} (CBK007), $\mu_{\Delta smc \Delta mksB}$=0.72 h^{-1} (CBK004), $\mu_{\Delta smc\ ParBeYFP}$=0.70 h^{-1} (CBK010), $\mu_{\Delta smc\ \Delta mksB\ ParBeYFP}$=0.71 h^{-1} (CBK011). **D)** Analysis in BHI medium: μ_{WT}=0.67 h^{-1}, $\mu_{\Delta parB}$=0.51 h^{-1} (CDC003), $\mu_{\Delta parB\ \Delta smc}$=0.46 h^{-1} (CBK002), $\mu_{\Delta parB\ \Delta mksB}$=0.49 h^{-1} (CBK003), $\mu_{\Delta parB\ \Delta smc\ \Delta mksB}$ =0.43 h^{-1} (CBK005). Source data of B-D are provided as a Source Data file. **E)** Differential maps between Δ*smc*-Δ*parB* (left) and Δ*parB*Δ*smc*-Δ*parB* (right) contact maps indicating the log of the ratio as in Fig. 2.

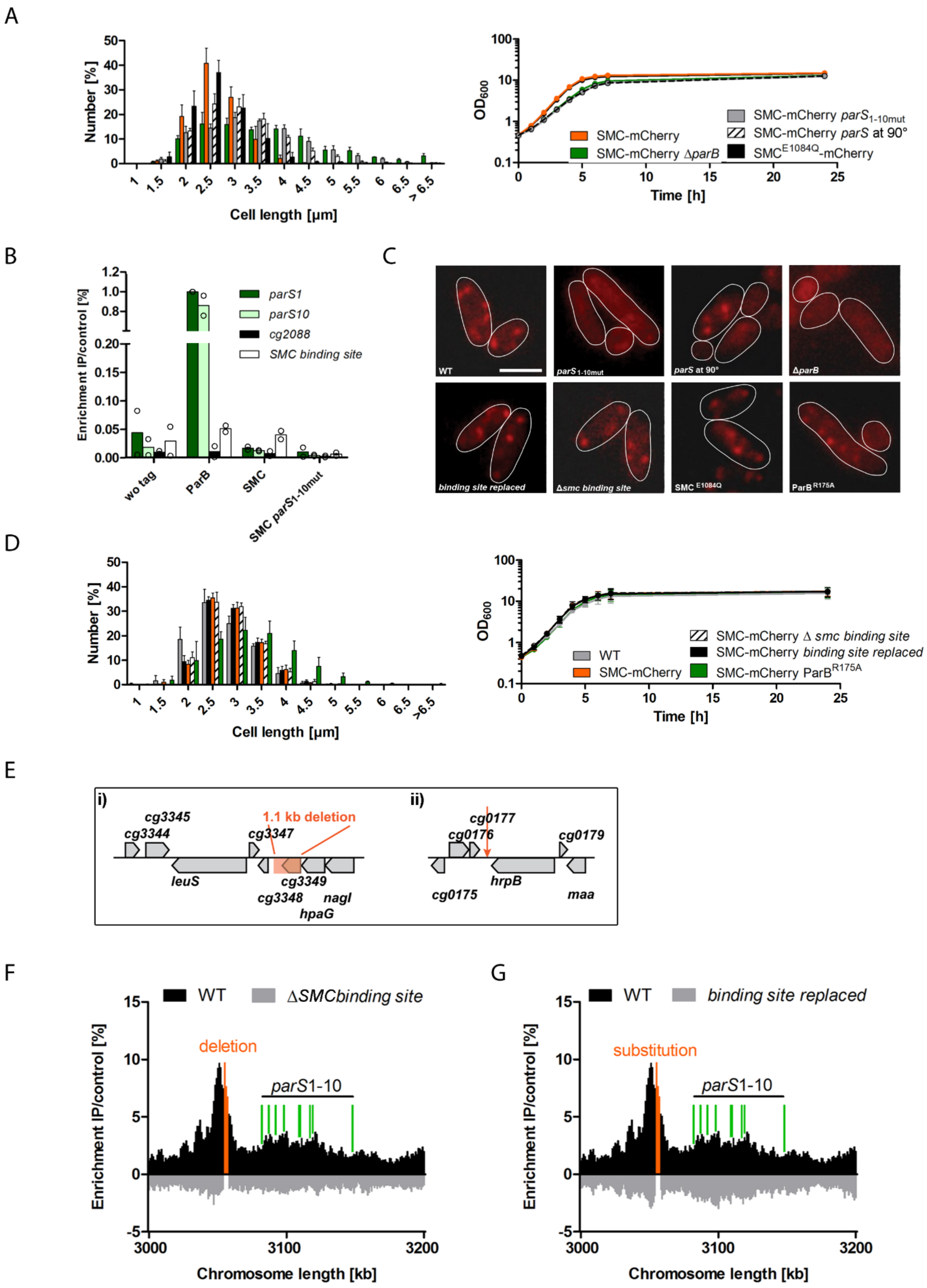
A
Number [%]
Cell length [µm]
OD600
Time [h]
SMC-mCherry
SMC-mCherry ΔparB
SMC-mCherry parS1-10mut
SMC-mCherry parS at 90°
SMCE1084Q-mCherry
B
Enrichment IP/control [%]
parS1
parS10
cg2088
SMC binding site
wo tag
ParB
SMC
SMC parS1-10mut
C
WT
parS 1-10mut
parS at 90°
ΔparB
binding site replaced
Δsmc binding site
SMC E1084Q
ParB R175A
D
Number [%]
Cell length [µm]
OD600
Time [h]
WT
SMC-mCherry
SMC-mCherry Δ smc binding site
SMC-mCherry binding site replaced
SMC-mCherry ParBR175A
E
i)
cg3345
cg3344
cg3347
1.1 kb deletion
leuS
cg3349
cg3348
nagI
hpaG
ii)
cg0177
cg0176
cg0179
cg0175
hrpB
maa
F
WT
ΔSMCbinding site
deletion
parS1-10
Enrichment IP/control [%]
Chromosome length [kb]
G
WT
binding site replaced
substitution
parS1-10
Enrichment IP/control [%]
Chromosome length [kb]

Supplementary Figure 10. Characterization of *C. glutamicum smc::smc-mCherry*

A) Growth (mean +/- s.d., n = 3) and cell length analysis (mean +/- s.d., n listed below) in BHI medium: $\mu_{SMC\text{-}mCherry}$=0.71 h^{-1} (CBK012) (n_1 = 493, n_2 = 380, n_3 = 434), $_{SMC\text{-}mCherry\ \Delta parB}$ =0.60 h^{-1} (CBK014) (n_1 = 445, n_2 = 404, n_3 = 401), $\mu_{SMC\text{-}mCherry\ parS1\text{-}10mut}$=0.56 h^{-1} (CBK032) (n_1 = 616, n_2 = 252, n_3 = 246), $\mu_{SMC\text{-}mCherry\ parS\ 3'\ cg904}$=0.55 h^{-1} (CBK045) (n_1 = 626, n_2 = 415, n_3 = 464), $\mu_{SMC\text{-}mCherryE1084Q}$=0.72 h^{-1} (CBK050) (n_1 = 367, n_2 = 531, n_3 = 339). **B)** ChIP-qPCR of the wild-type and cells harboring ParB- or SMC-mCherry tagged proteins in wild-type (SMC) or a *parS*-mutation (*parS*$_{1\text{-}10mut}$) background. **C)** Localization of SMC-mCherry foci in strain backgrounds described in A) and D). Images show mCherry fluorescence in representative cells; cell outlines are indicated (white lines). Scale bar, 2 µm. **D)** Analysis of cell lengths distributions(mean +/- s.d., n listed below) (left) and growth (mean +/- s.d., n = 3) in BHI medium (right) of *smc::smc-mCherry* mutant strain derivatives, growth rates: μ_{WT}=0.63 h^{-1} (n_1 = 199, n_2 = 508, n_3 = 284), $\mu_{SMC\text{-}mCherry}$=0.69 h^{-1} (CBK012) (n_1 = 306, n_2 = 362, n_3 = 550), $_{SMC\text{-}mCherry\ \Delta SMC\ binding\ site}$=0.68 h^{-1} (CBK034) (n_1 = 745, n_2 = 482, n_3 = 232), $\mu_{SMC\text{-}mCherry\ SMC\ binding\ site\ replaced}$=0.68 h^{-1} (CBK035) (n_1 = 612, n_2 = 452, n_3 = 268), $\mu_{SMC\text{-}mCherry\ ParBR175A}$=0.69 h^{-1} (CBK049) (n_1 = 379, n_2 = 339, n_3 = 379). Source data of A, B and D are provided in the Source Data file. **E)** Scheme showing mutation sites within the *C. glutamicum* CBK034 genome with a partially deleted SMC-enrichment region upstream of *parS* reinserted into intergenic region 3' of *cg0177*. i) ChIP-seq enrichment site of SMC-mCherry protein covers genes *cg3345* to *hpaG*. Deletion is indicated in orange (31.3 Mb). ii) Arrow points to intergenic insertion site of the 1.1 Kb fragment (0.15 Mb). **F)** Partial deletion of SMC-enrichment site collapses entire SMC-clustering nearby. *In vivo* ChIP-seq was performed using exponentially grown *C. glutamicum* SMC-mCherry expressing wild-type (upper y-axis, black) or CBK034 cells (lower y-axis, gray). The

deletion site and *parS* sequences are indicated in orange and by green lines, respectively. **G)** SMC-ChIP-seq analysis in CBK035 cells with a partial substitution of the SMC-binding site by a non-coding *B. subtilis* genome sequence of identical size abolishes SMC enrichment at this site.

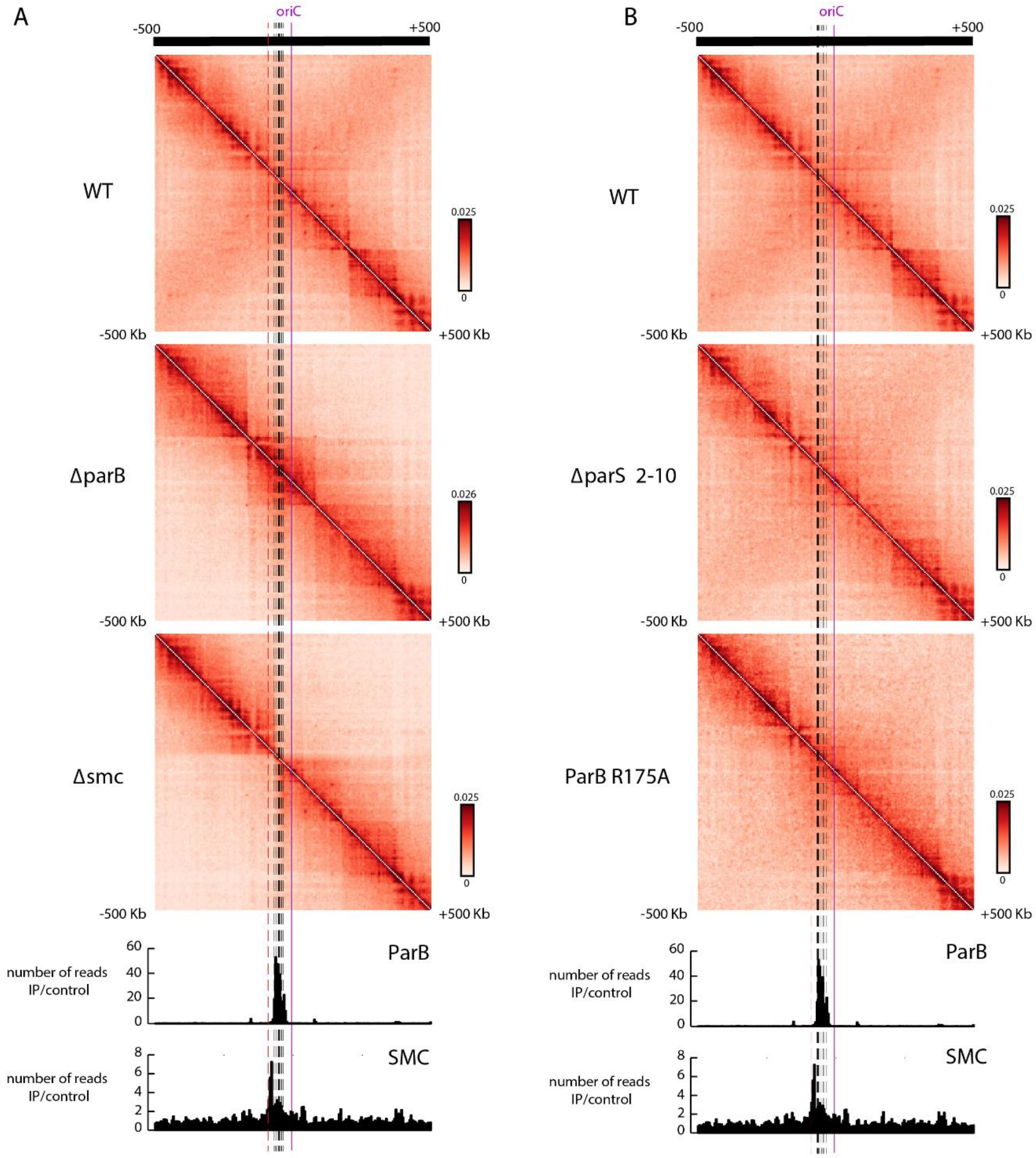

Supplementary Figure 11. Disorganization of *oriC*-regions in Par and SMC mutants.

A) Magnification of normalized genomic contact matrices of wild-type, Δ*parB* and Δ*smc*, (CDC003, CDC026) encompassing 500 Kb regions surrounding *oriC* (purple line). *parS* sites are indicated as dashed lines. Color codes as in Fig. 1 were applied. ParB and SMC enrichment zones are shown below the contact maps (ChIP signal

relative to the input in 5 Kb bins). **B)** Magnification of normalized genomic contact matrices of wild-type, *parS*$_{2\text{-}10mut}$ and *parB::parB*^{R175A} (CBK023, CBK047) as in A.

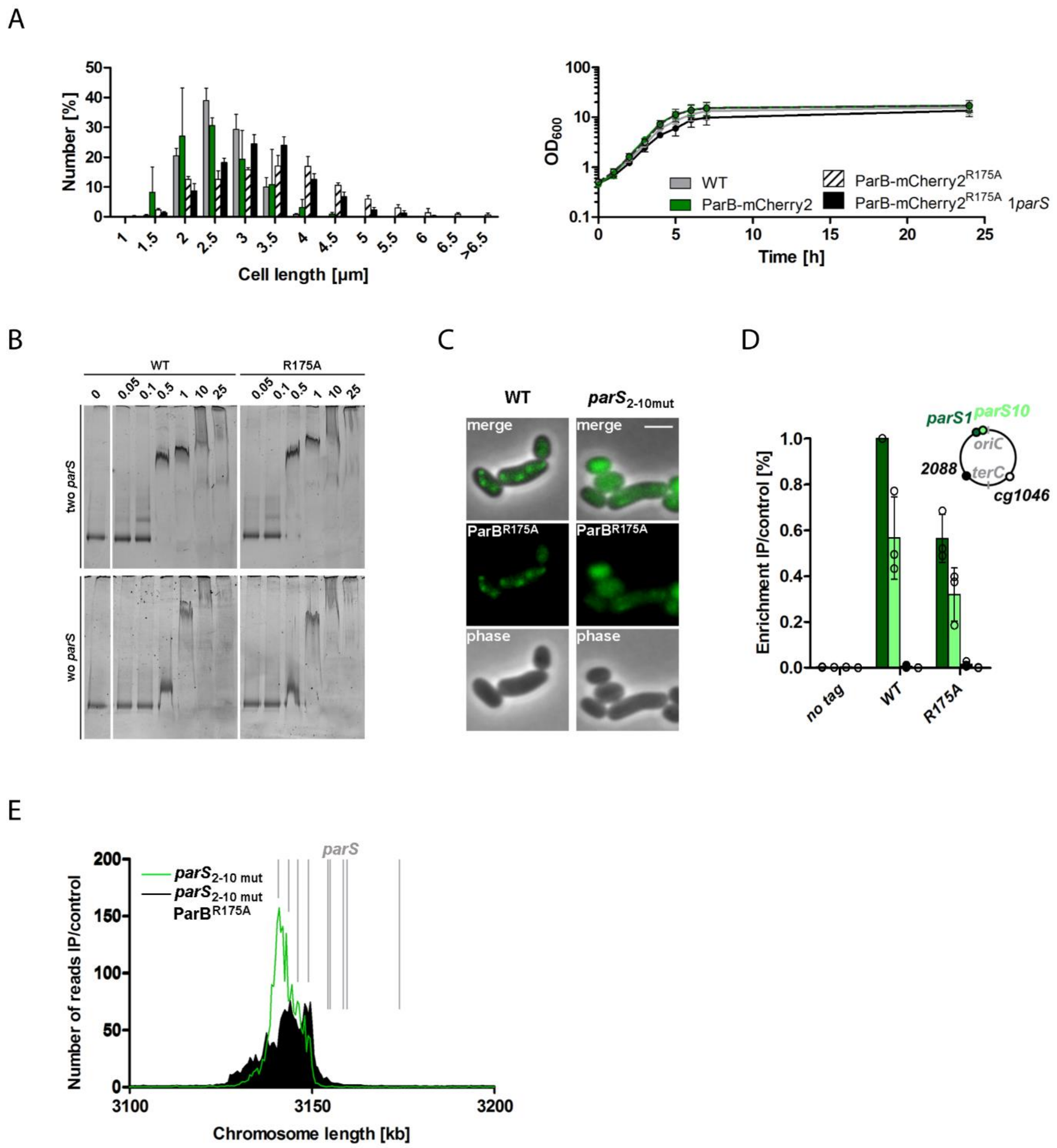

Supplementary Figure 12. ParBR175A compromises DNA binding *in vivo*.

A) Growth curves (mean +/- s.d., n = 3) and cell length distributions (mean +/- s.d., n listed below) of strain *parB::parB-mCherry* derivatives harboring $ParB^{R175A}$ mutant protein grown in BHI medium are shown; growth rates: μ_{WT}=0.63 h^{-1} (n_1 = 199, n_2 = 508, n_3 = 284), $\mu_{ParB\text{-}mCherry}$=0.69 h^{-1} (CBK006) (n_1 = 225, n_2 = 538, n_3 = 555), $\mu_{ParBR175A\text{-}mCherry}$=0.68 h^{-1} (CBK047) (n_1 = 339, n_2 = 564, n_3 = 297), $\mu_{ParBR175A\text{-}mCherry\ parS2\text{-}10mut}$=0.57 h^{-1} (CBK048) (n_1 = 403, n_2 = 243, n_3 = 272). **B)** Recombinant $ParB^{WT}$ and $ParB^{R175A}$

proteins bind *parS* sites and nonspecific DNA. Electrophoretic mobility shift assay of ParB proteins pre-incubated with 100 ng DNA sized 1084 bp with or without two *parS* sites. **C)** $ParB^{R175A}$-mCherry localization in wild-type and $parS_{2\text{-}10mut}$ mutant cells (CBK47, CBK048). Shown are phase contrast, mCherry fluorescence and an overlay of both channels. Scale bar, 2 µm. **D)** ParB-mCherry ChIP-qPCR at specified chromosomal markers performed with cells harboring ParB wild-type or R175A mutant. Standard deviations derive from biological triplicates; data normalization to wild-type *parS*1 signals. **E)** ChIP-seq enrichment at region of native *parS* cluster (gray); $ParB^{WT}$-(green, CBK027) or $ParB^{R175A}$-mCherry protein (black, CBK047) in strain background $parS_{2\text{-}10mut}$. Source data are provided as a Source Data file.

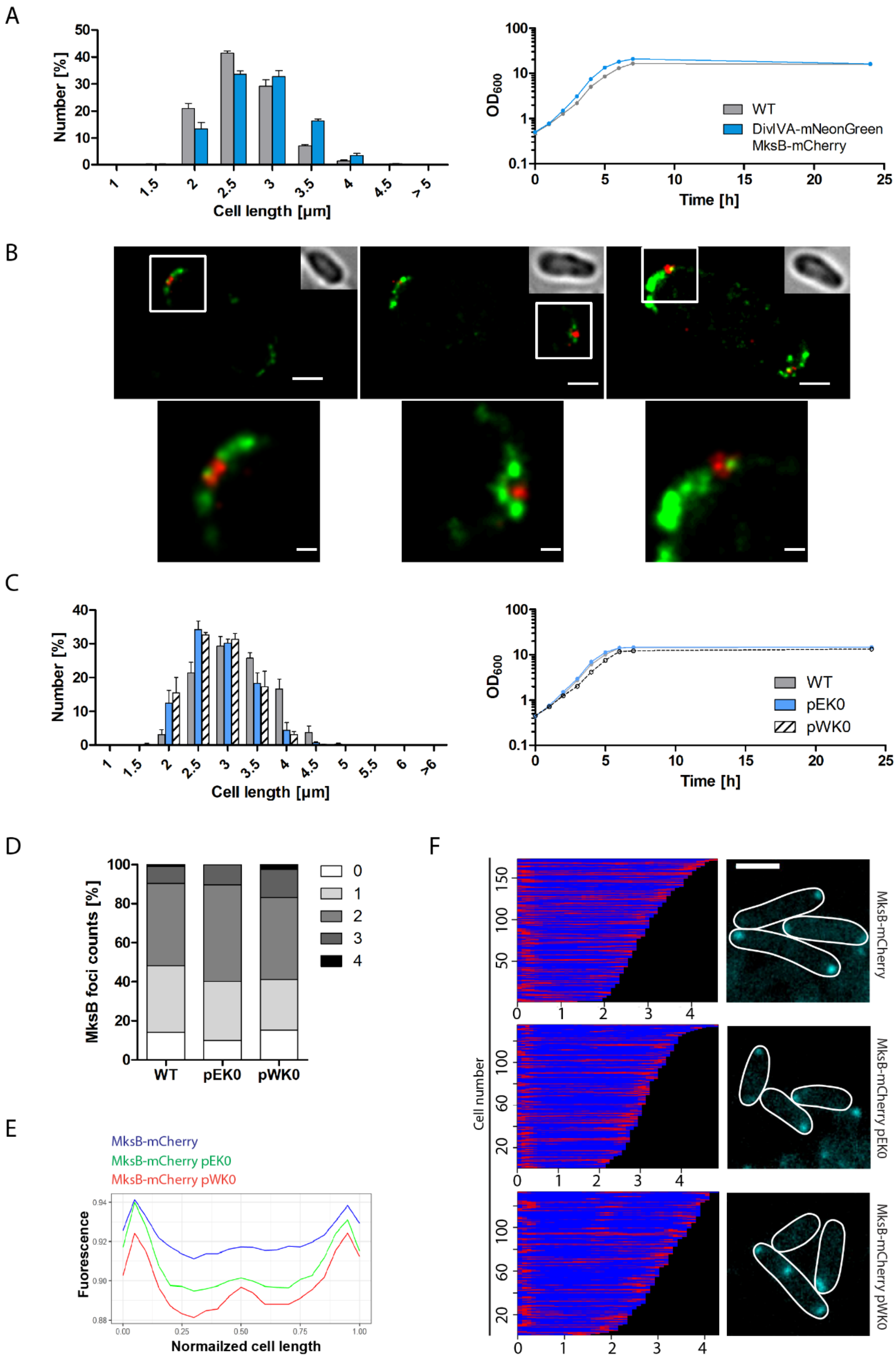
A
Number [%]
Cell length [µm]
OD600
Time [h]
WT
DivIVA-mNeonGreen
MksB-mCherry
B
C
Number [%]
Cell length [µm]
OD600
Time [h]
WT
pEK0
pWK0
D
MksB foci counts [%]
WT
pEK0
pWK0
0
1
2
3
4
E
MksB-mCherry
MksB-mCherry pEK0
MksB-mCherry pWK0
Fluorescence
Normailzed cell length
F
Cell number
MksB-mCherry
MksB-mCherry pEK0
MksB-mCherry pWK0

Supplementary Fig. 13 Polar MksB-mCherry localization in presence and absence of plasmids.

A) Cell length distribution (mean +/- s.d., n listed below) (left) and growth curves (mean +/- s.d., n = 3) (right) of wild-type and *divIVA::divIVA-mNeonGreen mksB::mksB-mCherry* cells (CBK092). Analyses were performed in biological triplicates using BHI medium; growth rates: μ_{WT}=0.68 h^{-1} (n_1 = 588, n_2 = 1646, n_3 = 820), $\mu_{DivIVA\text{-}mNeonGreen\ MksB\text{-}mCherry}$ =0.69 h^{-1} (n_1 = 1513, n_2 = 727, n_3 = 1096). **B)** Super resolution imaging of MksB (red) and DivIVA localization (green). Top: Gaussian rendering and bright field of three exemplary *C. glutamicum divIVA::divIVA-mNeonGreen mksB::mksB-PAmCherry* cells (scale bar: 0.5 µm). Below: Magnification of polar regions (scale bar, 0.1 µm). See “Material and Methods: PALM microscopy” for detailed description of imaging conditions and sample preparation. **C)** Cell length (mean +/- s.d., n listed below) and growth (mean +/- s.d., n = 3) in BHI medium of strains *mksB::mksB-mCherry* without plasmid (CBK015) or containing either pEK0 or pWK0 (CBK089, CBK088) were analyzed in biological triplicates; growth rates: μ_{WT} =0.68 (n_1 = 1292, n_2 = 1312, n_3 = 1289), μ_{pEK0}=0.69 (n_1 = 760, n_2 = 848, n_3 = 583), μ_{pWK0}=0.68 (n_1 = 824, n_2 = 585, n_3 = 1498). **D)** MksB-mCherry foci numbers per cell were determined in strains named above, n > 100. **E)** MksB-mCherry fluorescence profiles along longitudinal cell axes were extracted from above-named strains, normalized to cell length (n>100) and binned (bin = 0.05 µm). Fluorescence (a.u.) is plotted normalized to maximal intensity values. **F)** Right: Cellular MksB fluorescence profiles summarized in E) are depicted in demographs sorted by cell length with a color scheme ranging from blue (low intensity values) to red (high intensity values). Left: Microscopy images showing MksB-mCherry fluorescence (cyan) of exemplary cells with contours indicated by white lines; scale bar, 2 µm. Source data are provided as a Source Data file.

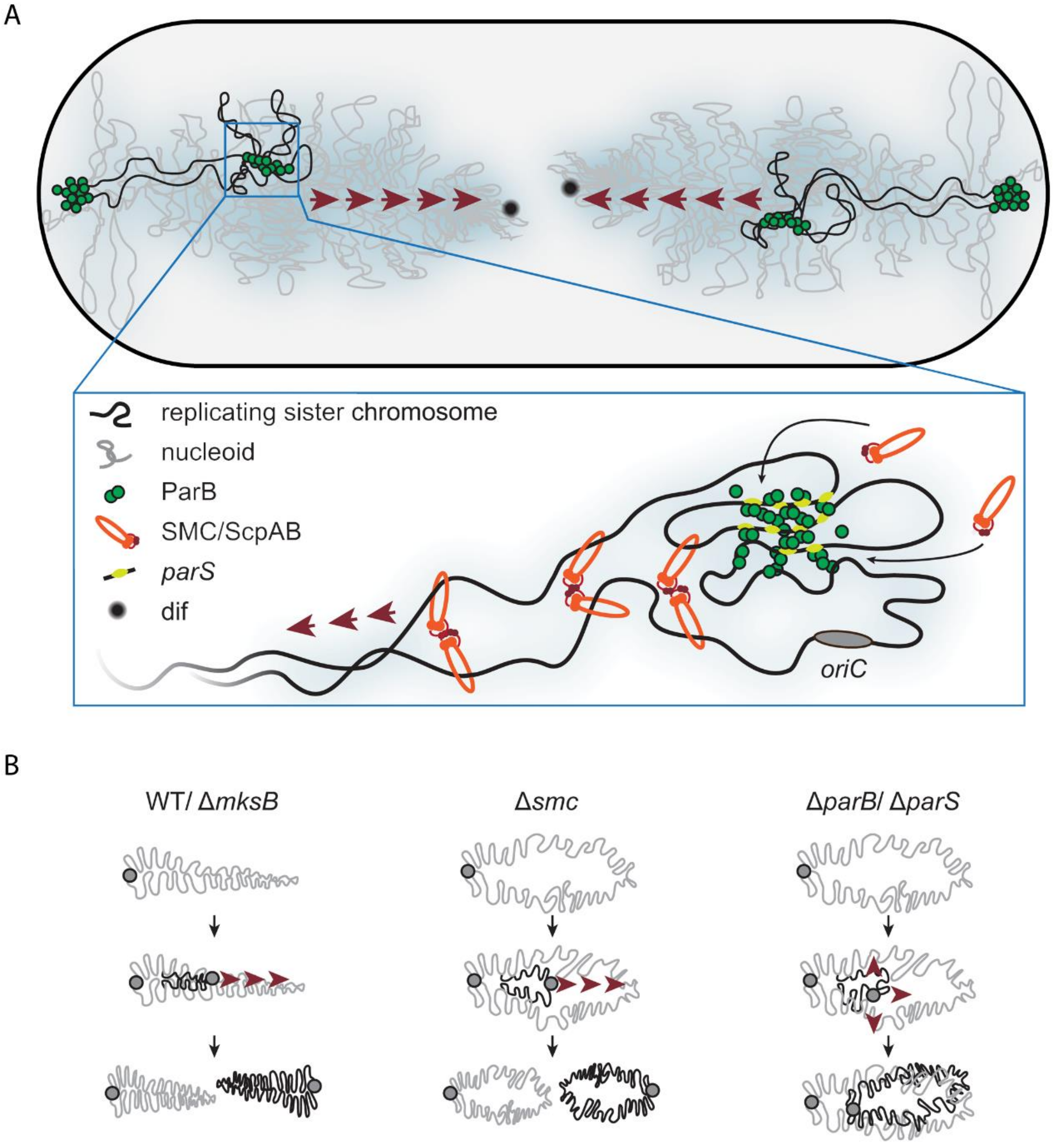

Supplementary Figure 14. Model of ParB- and SMC-mediated chromosome organization in *C. glutamicum*.

A) Top: Illustration of chromosome segregation in a diploid *C. glutamicum* cell. Newly replicated ParB-*oriC*s complexes translocate from cell poles towards *terC*s at septal positions via a ParABS system, where ParB nucleocomplexes use ParA-bound sister chromosomal loci as transient tethers for translocation across the nucleoid. Below: Condensins SMC/ScpAB are loaded ParB-dependently at *parS* sites and relocate to

distant chromosomal regions (red arrows) causing inter-linkage of chromosomal arms; here illustration is based on a two-translocator model. **B)** Cartoon showing mutant phenotypes of overall chromosome folding and segregation compared to wild-type (WT). Replication *oriC* (grey mark), chromosome (grey line) and replicating sister DNA (black line) are indicated; red arrows indicate direction of DNA segregation.

Supplementary Table 1. Percentage of DNA-free cells of relevant *C. glutamicum* RES167 strains used in this study.

Color code visualizes severity of nucleoid missegregation: virtually none (0*), up to 10 % (<10) and more than 20 % (>20) DNA-free cells, (n>1000).

Strain	Genotype	Anucleate cells [%]	Missegregation group
**RES 167**	wild-type	0	0*
**CDC003**	Δ*parB*	26.14	>20
**CDC026**	Δ*smc*	0.1	0*
**CBK001**	Δ*mksB*	0	0*
**CBK002**	Δ*parB* Δ*smc*	27.08	>20
**CBK003**	Δ*parB* Δ*mksB*	24.95	>20
**CBK004**	Δ*smc* Δ*mksB*	0	0*
**CBK005**	Δ*parB* Δ*smc* Δ*mksB*	25.62	>20
**CBK006**	*parB::parB-mCherry2*	0	0*
**CBK007**	*parB::parB-eYFP*	0.1	0*
**CBK010**	*Δsmc parB::parB-eYFP*	4.25	<10
**CBK011**	*Δsmc ΔmksB parB::parB-eYFP*	3.98	<10
**CBK012**	*smc::smc-mCherry*	0	0*
**CBK014**	*smc::smc-mCherry* Δ*parB*	24.18	>20
**CBK015**	*mksB::mksB-mCherry*	0	0*
**CBK023**	*parS*$_{2-10mut}$	0.19	0*
**CBK024**	*parS*$_{1-10mut}$	29.27	>20
**CBK025**	*parS*$_{2-10mut}$ *parB::parB-eYFP*	6.71	<10
**CBK026**	*parS*$_{1-10mut}$ *parB::parB-eYFP*	28.5	>20
**CBK032**	*parS*$_{1-10mut}$ *smc::smc-mCherry*	30.38	>20
**CBK034**	*smc::smc-mCherry* Δ *SMC loading site*	0	0*
**CBK035**	*smc::smc-mCherry SMC loading site replaced*	0	0*
**CBK040**	*parS*$_{1-10mut}$ *parS* 3‘ *cg0108* (9.5°) *parB::parB-eYFP*	25.33	>20
**CBK041**	*parS*$_{1-10mut}$ *parS* 3‘ *cg0904* (90°) *parB::parB-eYFP*	23.48	>20
**CBK043**	*parS*$_{1-10mut}$ *parS* 3‘ *cg2563* (270°) *parB::parB-eYFP*	24.28	>20
**CBK044**	*parS*$_{1-10mut}$ *int::parS* (180°) *parB::parB-eYFP*	24.98	>20
**CBK045**	*parS*$_{1-10mut}$ *parS* 3‘ *cg0904* (90°) *smc::smc-mCherry*	25.07	>20
**CBK047**	*parB::parB*^{R175A}*-mCherry2*	17.83	>20
**CBK048**	*parB::parB*^{R175A}*-mCherry2 parS*$_{2-10mut}$	31.74	>20
**CBK049**	*smc::smc-mCherry parB::parB*^{R175A}	19.09	>20

**CBK051**	*smc::smc*^{E1084Q}*-mCherry*	0	0*
**CBK090**	*parS*$_{1\text{-}9mut}$	0.18	0*
**CBK091**	*parS*$_{1\text{-}9mut}$ *parB::parB-mCherry2*	1.74	<10
**CBK092**	*divIVA::divIVA-mNeonGreen*, *mksB::mksB-mCherry*	0	0*

Supplementary Table 2. Average DNA amount per cell after replication runout determined by flow cytometry in triplicates, including standard deviation.

**Stain**	**Genotype**	**Chromosome numbers per cell**
**RES167**	Wild-type	5.99 ± 0.50
**CDC002**	Δ*parB*	6.47 ± 0.07
**CDC026**	Δ*smc*	6.00 ± 0.51
**CBK001**	Δ*mksB*	6.53 ± 0.10
**CBK002**	Δ*parB Δsmc*	6.38 ± 0.62
**CBK004**	Δ*smc* Δ*mksB*	5.90 ± 0.64
**CBK025**	*parS*$_{2\text{-}10mut}$	5.65 ± 0.16
**CBK026**	*parS*$_{1\text{-}10mut}$	5.87 ± 0.45
**CBK043**	*parS*$_{1\text{-}10mut}$ *parS* 3‘ *cg0904* (90°) *parB*∷*parB-eYFP*	6.32 ± 0.57
**CBK051**	*parB*∷*parB*^{R175A}*-mCherry2*	5.71 ± 0.35
**CBK054**	*smc*∷*smc*^{E1084Q}	5.67 ± 0.82

Supplementary References

1. Pfeifer-Sancar, K., Mentz, A., Rückert, C. & Kalinowski, J. Comprehensive analysis of the *Corynebacterium glutamicum* transcriptome using an improved RNAseq technique. *BMC Genomics* **14**, 888, doi:10.1186/1471-2164-14-888 (2013).
2. Lioy, V. S. *et al.* Multiscale structuring of the *E. coli* chromosome by nucleoid-associated and condensin proteins. *Cell* **172**, 771-783.e718, doi:10.1016/j.cell.2017.12.027 (2018).
3. Yang, T. *et al.* HiCRep: assessing the reproducibility of Hi-C data using a stratum-adjusted correlation coefficient. *Genome Res* **27**, 1939-1949, doi:10.1101/gr.220640.117 (2017).
4. Ducret, A., Quardokus, E. M. & Brun, Y. V. MicrobeJ, a tool for high throughput bacterial cell detection and quantitative analysis. *Nat Microbiol* **1**, 16077, doi:10.1038/nmicrobiol.2016.77 (2016).
