# Supplementary material for: Chromosome organization by a conserved condensin-ParB system in the actinobacterium Corynebacterium glutamicum
Source: Nat Commun. 2020 Mar 20;11:1485. doi: 10.1038/s41467-020-15238-4 (PMC7083940; doi:10.1038/s41467-020-15238-4)
Supplement: Supplementary file 2 — Description of Additional Supplementary Files [file 41467_2020_15238_MOESM2_ESM.pdf]

Description of Additional Supplementary Files

File Name: Supplementary Data 1
Description: List of oligonucleotides

File Name: Supplementary Data 2
Description: List of strains and plasmids
