# Supplementary material for: Chromosome organization by a conserved condensin-ParB system in the actinobacterium Corynebacterium glutamicum
Source: Nat Commun. 2020 Mar 20;11:1485. doi: 10.1038/s41467-020-15238-4 (PMC7083940; doi:10.1038/s41467-020-15238-4)
Supplement: Supplementary file 5 — Reporting Summary [file 41467_2020_15238_MOESM5_ESM.pdf]

nature research

Corresponding author(s): Marc Bramkamp
Martial Marbouty

Last updated by author(s): 15/02/2020

Reporting Summary

Nature Research wishes to improve the reproducibility of the work that we publish. This form provides structure for consistency and transparency in reporting. For further information on Nature Research policies, see Authors & Referees and the Editorial Policy Checklist .

Statistics

For all statistical analyses, confirm that the following items are present in the figure legend, table legend, main text, or Methods section.

n/a	Confirmed	
☐	☒	The exact sample size (*n*) for each experimental group/condition, given as a discrete number and unit of measurement
☐	☒	A statement on whether measurements were taken from distinct samples or whether the same sample was measured repeatedly
☐	☒	The statistical test(s) used AND whether they are one- or two-sided<br>*Only common tests should be described solely by name; describe more complex techniques in the Methods section.*
☒	☐	A description of all covariates tested
☐	☒	A description of any assumptions or corrections, such as tests of normality and adjustment for multiple comparisons
☐	☒	A full description of the statistical parameters including central tendency (e.g. means) or other basic estimates (e.g. regression coefficient) AND variation (e.g. standard deviation) or associated estimates of uncertainty (e.g. confidence intervals)
☐	☒	For null hypothesis testing, the test statistic (e.g. *F*, *t*, *r*) with confidence intervals, effect sizes, degrees of freedom and *P* value noted<br>*Give P values as exact values whenever suitable.*
☒	☐	For Bayesian analysis, information on the choice of priors and Markov chain Monte Carlo settings
☒	☐	For hierarchical and complex designs, identification of the appropriate level for tests and full reporting of outcomes
☒	☐	Estimates of effect sizes (e.g. Cohen's *d*, Pearson's *r*), indicating how they were calculated

Our web collection on statistics for biologists contains articles on many of the points above.

Software and code

Policy information about availability of computer code

Data collection	ZenBlack software v2.1 SP3 14.0.4.201 (Zeiss)
Data analysis	R-studio v1.1.453 (2009-2018 RStudio, Inc.)(open source)<br>R version v3.5.0 (2018-04-23)(open source)<br>Fiji (ImageJ) v2.0.0-rc-59/1.51k (open source)<br>MaxQuant software suite (open source)<br>BD Accuri C6 Plus software (BD Biosciences)<br>HiCRep (1.10.0; open source)<br>CLC Genomics Workbench (Qiagen)<br>Bio Rad-IQ™5 software version 2.1. (BioRad)<br>The custom computer algorithm used in the analysis of PALM data has been deposited in Github (https://github.com/GiacomoGiacomelli/ParB-clustering-protein-profiling)

For manuscripts utilizing custom algorithms or software that are central to the research but not yet described in published literature, software must be made available to editors/reviewers. We strongly encourage code deposition in a community repository (e.g. GitHub). See the Nature Research guidelines for submitting code & software for further information.

Data

Policy information about availability of data

All manuscripts must include a data availability statement. This statement should provide the following information, where applicable:
- Accession codes, unique identifiers, or web links for publicly available datasets
- A list of figures that have associated raw data
- A description of any restrictions on data availability

Hi-C, flow cytometry and ChIP-Seq data have been deposited in public databases. Accession numbers are given in the main text. Whole genome data of C.

glutamicum were accessed from GeneBank (GeneBankID: BX927147.1). RNA-seq data for C. glutamicum were recovered from ENA (Project PRJEB4788). The authors declare that the data supporting the findings of this study are available within the paper and raw data file of all plots and gel images have been submitted with this paper. Data can also be obtained from the corresponding authors upon request.

Field-specific reporting

Please select the one below that is the best fit for your research. If you are not sure, read the appropriate sections before making your selection.

☒ Life sciences ☐ Behavioural & social sciences ☐ Ecological, evolutionary & environmental sciences

For a reference copy of the document with all sections, see nature.com/documents/nr-reporting-summary-flat.pdf

Life sciences study design

All studies must disclose on these points even when the disclosure is negative.

Sample size	Experiments were at least done in biological triplicates. Sample sizes for single cell experiments image analyses were always above n=200. For flow cytometry more than 10.000 single cells were counted. Sample sizes were chosen based on standards in the field.
Data exclusions	We did not exclude datapoints. A Source Data file with all raw data is submitted along with this paper.
Replication	Number of experimental replications are indicated in the text for every experiment. We did not observe severe differences between experimental replicates. Covariates were controlled by using identical experimental conditions, buffers and solutions as well as identically treated samples. Identical phenotypes of strains were oberserved in independent experiments.
Randomization	Samples were not randomized. Single cell data (images and flow cytometry) rely on large numbers.
Blinding	We did not use blinding. This was unecessary since we rely on automated image analyses and statistical data evaluation.

Reporting for specific materials, systems and methods

We require information from authors about some types of materials, experimental systems and methods used in many studies. Here, indicate whether each material, system or method listed is relevant to your study. If you are not sure if a list item applies to your research, read the appropriate section before selecting a response.

Materials & experimental systems

n/a	Involved in the study	
☐	☒	Antibodies
☒	☐	Eukaryotic cell lines
☒	☐	Palaeontology
☒	☐	Animals and other organisms
☒	☐	Human research participants
☒	☐	Clinical data

Methods

n/a	Involved in the study	
☐	☒	ChIP-seq
☐	☒	Flow cytometry
☒	☐	MRI-based neuroimaging

Antibodies

Antibodies used	α-mCherry antibody (BioVision Inc.), Catalog #: 5993, 10 µg of antibody were used per reaction for ChIP. α-mCherry antibody (BioVision Inc.), Catalog #: 5993 was used for western blot in 1:2000 dilution. Secondary antibody was an anti-Rabbit IgG (whole molecule)–Alkaline Phosphatase antibody produced in goat from Sigma (Catalog #A0418) used in 1:10.000 dilutions.
Validation	Specificity of antibodies was tested in western blots against cell lysate of strains expressing mCherry fusion proteins.

ChIP-seq

Data deposition

☒ Confirm that both raw and final processed data have been deposited in a public database such as GEO.

☐ Confirm that you have deposited or provided access to graph files (e.g. BED files) for the called peaks.

Data access links<br>*May remain private before publication.*	https://www.ncbi.nlm.nih.gov/sra/?term=prjna529385<br>Accession codes prjna529385<br>Final graphs are given in the manuscript and can be requested from the corresponding authors upon request. All raw data are given in the supplemental material file.

Files in database submission

Files for accession number PRJNA529385:
1.
Anti-mCherry ChIP-seq of C. glutamicum: ParB-mCherry CBK091 strain, immunoprecipitate
1 ILLUMINA (Illumina MiSeq) run: 1.6M spots, 784.2M bases, 451.4Mb downloads
Accession:
SRX6979399
Anti-mCherry ChIP-seq of C. glutamicum: ParB-mCherry CBK091 strain, extract sample
1 ILLUMINA (Illumina MiSeq) run: 2.4M spots, 1.2G bases, 626.2Mb downloads
Accession:
SRX6979398
Anti-mCherry ChIP-seq of C. glutamicum: SMC-mCherry CBK049 strain, extract sample replicate 1
1 ILLUMINA (Illumina MiSeq) run: 1.6M spots, 543M bases, 343.4Mb downloads
Accession:
SRX5581481
Anti-mCherry ChIP-seq of C. glutamicum: SMC-mCherry CBK049 strain, immunoprecipitate replicate 1
1 ILLUMINA (Illumina MiSeq) run: 268 spots, 80,163 bases, 136,899b downloads
Accession:
SRX5581480
Anti-mCherry ChIP-seq of C. glutamicum: SMC-mCherry CBK051 strain, extract sample
1 ILLUMINA (Illumina MiSeq) run: 2.3M spots, 1.2G bases, 636Mb downloads
Accession:
SRX5581479
Anti-mCherry ChIP-seq of C. glutamicum: SMC-mCherry CBK051 strain, immunoprecipitate
1 ILLUMINA (Illumina MiSeq) run: 1.9M spots, 834.1M bases, 449.5Mb downloads
Accession:
SRX5581478
Anti-mCherry ChIP-seq of C. glutamicum: SMC-mCherry CBK014 strain, extract sample
1 ILLUMINA (Illumina MiSeq) run: 2.8M spots, 1.4G bases, 818.2Mb downloads
Accession:
SRX5581477
Anti-mCherry ChIP-seq of C. glutamicum: SMC-mCherry CBK014 strain, immunoprecipitate
1 ILLUMINA (Illumina MiSeq) run: 1.3M spots, 647.8M bases, 334.1Mb downloads
Accession:
SRX5581476
Anti-mCherry ChIP-seq of C. glutamicum: SMC-mCherry CBK034 strain, extract sample replicate 2
1 ILLUMINA (Illumina MiSeq) run: 1.5M spots, 771.5M bases, 451.4Mb downloads
Accession:
SRX5581475
Anti-mCherry ChIP-seq of C. glutamicum: SMC-mCherry CBK034 strain, immunoprecipitate replicate 2
1 ILLUMINA (Illumina MiSeq) run: 1.2M spots, 598.8M bases, 354.5Mb downloads
Accession:
SRX5581474
Anti-mCherry ChIP-seq of C. glutamicum: SMC-mCherry CBK034 strain, extract sample replicate 1
1 ILLUMINA (Illumina MiSeq) run: 1.3M spots, 645.8M bases, 390.7Mb downloads
Accession:
SRX5581473
Anti-mCherry ChIP-seq of C. glutamicum: SMC-mCherry CBK034 strain, immunoprecipitate replicate 1
1 ILLUMINA (Illumina MiSeq) run: 1.4M spots, 705.8M bases, 360.9Mb downloads
Accession:
SRX5581472
Anti-mCherry ChIP-seq of C. glutamicum: ParB-mCherry CBK030 strain, extract sample
1 ILLUMINA (Illumina MiSeq) run: 1.1M spots, 490.9M bases, 279.9Mb downloads
Accession:
SRX5581471
Anti-mCherry ChIP-seq of C. glutamicum: ParB-mCherry CBK030 strain, immunoprecipitate
1 ILLUMINA (Illumina MiSeq) run: 2.3M spots, 903.4M bases, 519.4Mb downloads
Accession:
SRX5581470
Anti-mCherry ChIP-seq of C. glutamicum: ParB-mCherry CBK027 strain, extract sample replicate 1
1 ILLUMINA (Illumina MiSeq) run: 83,580 spots, 30.8M bases, 20.7Mb downloads
Accession:
SRX5581469
Anti-mCherry ChIP-seq of C. glutamicum: ParB-mCherry CBK027 strain, immunoprecipitate replicate 1
1 ILLUMINA (Illumina MiSeq) run: 7,705 spots, 2.6M bases, 1.8Mb downloads
Accession:
SRX5581468
Anti-mCherry ChIP-seq of C. glutamicum: ParB-mCherry CBK027 strain, extract sample replicate 2

1 ILLUMINA (Illumina MiSeq) run: 1.9M spots, 946.3M bases, 544.2Mb downloads
Accession:
SRX5581467
Anti-mCherry ChIP-seq of C. glutamicum: ParB-mCherry CBK027 strain, immunoprecipitate replicate 2
1 ILLUMINA (Illumina MiSeq) run: 1.2M spots, 580.2M bases, 317.5Mb downloads
Accession:
SRX5581466
Anti-mCherry ChIP-seq of C. glutamicum: ParB-mCherry CBK047 strain, extract sample replicate 1
1 ILLUMINA (Illumina MiSeq) run: 975,293 spots, 315.9M bases, 201Mb downloads
Accession:
SRX5581465
Anti-mCherry ChIP-seq of C. glutamicum: ParB-mCherry CBK047 strain, immunoprecipitate replicate 1
1 ILLUMINA (Illumina MiSeq) run: 3,122 spots, 1.1M bases, 838,959b downloads

Files for accession number PRJNA525583:

3C:Cg:WT
1 ILLUMINA (NextSeq 550) run: 171.4M spots, 12.5G bases, 4.9Gb downloads
Accession:
SRX5464577
3C:Cg:smc
1 ILLUMINA (NextSeq 550) run: 75.9M spots, 5.6G bases, 2.2Gb downloads
Accession:
SRX5464576
3C:Cg:smcmksB
1 ILLUMINA (NextSeq 550) run: 94.7M spots, 6.9G bases, 2.7Gb downloads
Accession:
SRX5464575
3C:Cg:parB_mCherry
1 ILLUMINA (NextSeq 550) run: 55.1M spots, 4G bases, 1.6Gb downloads
Accession:
SRX5464574
3C:Cg:ParBR175A
1 ILLUMINA (NextSeq 550) run: 28.4M spots, 2G bases, 778Mb downloads
Accession:
SRX5464573
3C:Cg:parS1-10
1 ILLUMINA (NextSeq 550) run: 36.6M spots, 2.7G bases, 1.1Gb downloads
Accession:
SRX5464572
3C:Cg:parS2-10
1 ILLUMINA (NextSeq 550) run: 82M spots, 6G bases, 2.4Gb downloads
Accession:
SRX5464571
3C:Cg:0904smc
1 ILLUMINA (NextSeq 550) run: 40.5M spots, 2.9G bases, 1.1Gb downloads
Accession:
SRX5464570
3C:Cg:0904WT
1 ILLUMINA (NextSeq 550) run: 78.1M spots, 5.6G bases, 2.1Gb downloads
Accession:
SRX5464569
3C:Cg:mksB
1 ILLUMINA (NextSeq 550) run: 94.8M spots, 6.9G bases, 2.8Gb downloads
Accession:
SRX5464568
3C:Cg:parB
1 ILLUMINA (NextSeq 550) run: 167.9M spots, 12.2G bases, 4.7Gb downloads
Accession:
SRX5464567

Genome browser session
(e.g. UCSC)

no longer applicable

Methodology

Replicates	Experiments were done in biological triplicates or doublicates as stated in the final manuscript.
Sequencing depth	Data were generated by illumina sequencing with 2x250 bp paired-end sequencing. Around 2 million reads were obtained and less than 1% reads were not mapped.
Antibodies	α-mCherry antibody (BioVision Inc.), Catalog #: 5993. 10 µg of antibody were used per reaction for ChIP.
Peak calling parameters	The ratio of number of reads for relevant peaks taken into account >two-fold in ChIP-seq, no peak calling algorithm used, enrichments further confirmed by replicates.
Data quality	We used qPCR to verify peaks identified by ChIP-Seq.
Software	Alignments were performed in CLC Main workbench (algorithm), Normalization and binning of data were performed using the online platform https://usegalaxy.org, deep tool "bam compare"<br>number of reads counted per bin, Scaling : samples with different sequencing depth are scaled according to total read count, Comparison of two samples: by ratio of number of reads per bin

Flow Cytometry

Plots

Confirm that:

☒ The axis labels state the marker and fluorochrome used (e.g. CD4-FITC).

☒ The axis scales are clearly visible. Include numbers along axes only for bottom left plot of group (a 'group' is an analysis of identical markers).

☒ All plots are contour plots with outliers or pseudocolor plots.

☒ A numerical value for number of cells or percentage (with statistics) is provided.

Methodology

Sample preparation	Exponentially growing cultures were treated with 25 µg/ml chloramphenicol for more than 4 h in order to induce replication runouts. Cells were fixated in 70% ethanol (1:9 v/v) and washed once in PBS. Cells were than stained with cybergreen
Instrument	BD Accuri C6 (BD Biosciences) equipped with a 488 nm laser.
Software	Data analysis was performed using the BD Accuri C6 Plus software (BD Biosciences). Filter (SybrGreenI) FL1 533/30
Cell population abundance	Does not apply since we count all cells, except cell cluster (indicated by side scatter).
Gating strategy	(1) 200000 events in R2 (threshold to distinguish stained cells from dust particles/ non-stained debris), (2) exclude cell aggregates derived from EtOH fixation: gating SSC/height of signal, 2 populations included due to taking also long cells into account that are about to divide (3) histogram SybrGreen(Fl1)channel/counts . Sample images of gating strategy are shown in the supplementary raw data file.

☒ Tick this box to confirm that a figure exemplifying the gating strategy is provided in the Supplementary Information.
